# Judicious Molecular Design of 5*H*‑Dithieno[3,2‑b:2′,3′‑d]Pyran‐based Hole‐Transporting Materials for Highly Efficient and Stable Perovskite Solar Cells

**DOI:** 10.1002/advs.202410666

**Published:** 2024-11-28

**Authors:** Kun‐Mu Lee, Chia‐Hui Lin, Chia‐Chi Chang, Ting‐Yu Yang, Wei‐Hao Chiu, Wei‐Chen Chu, Ya‐Ho Chang, Sie‐Rong Li, Shih‐I Lu, Hsiao‐Chi Hsieh, Kang‐Ling Liau, Chia Hui Hu, Chih‐Hung Chen, Yun‐Shuo Liu, Wei‐Chun Chou, Mandy M. Lee, Shih‐Sheng Sun, Yu‐Tai Tao, Yan‐Duo Lin

**Affiliations:** ^1^ Department of Chemical and Materials Engineering Chang Gung University Taoyuan 33302 Taiwan; ^2^ Center for Sustainability and Energy Technologies Chang Gung University Taoyuan 33302 Taiwan; ^3^ Division of Neonatology Department of Pediatrics Chang Gung Memorial Hospital Linkou Taoyuan 33305 Taiwan; ^4^ College of Environment and Resources Ming Chi University of Technology New Taipei City 24301 Taiwan; ^5^ Department of Chemistry Soochow University Taipei 11102 Taiwan; ^6^ Institute of Chemistry Academia Sinica Taipei 115024 Taiwan; ^7^ Department of Chemical and Materials Engineering Tamkang University New Taipei City 24301 Taiwan; ^8^ Department of Chemistry National Central University Taoyuan 32001 Taiwan; ^9^ Department of Materials Science and Engineering National Taiwan University of Science and Technology Taipei 106335 Taiwan

**Keywords:** dithienopyran‐based derivatives, fluorine‐substituted small molecule, hole‐transporting materials, long‐term stability, perovskite solar cells, photo‐energy conversion

## Abstract

The structural modification of hole‐transporting materials (HTMs) is an effective strategy for enhancing photovoltaic performance in perovskite solar cells (PSCs). Herein, a series of dithienopyran (DTP)‐based HTMs (**Me‐H**, **Ph‐H**, **CF3‐H**, **CF3‐mF**, and **CF3‐oF**) is designed and synthesized by substituting different functional groups on the DTP unit and are used fabricating PSCs. In comparison with **Me‐H** having two methyl substituents on the dithienopyrano ring, the **Ph‐H** having two phenyl substituents on the ring exhibits higher PCEs. Notably, the incorporation of trifluoromethyl groups in **CF3‐H** endows the molecule with a larger dipole moment, deeper HOMO energy level, better film morphology, closer molecular stacking, more efficient defect‐passivation, enhanced hydrophobicity, and better photovoltaic performance when compared with the **Ph‐H** counterpart. Furthermore, the HTMs of **CF3‐mF** and **CF3‐oF**, which feature fluorine‐substituted triphenylamine, demonstrated excellent film‐forming properties, more suitable energy levels, enhanced charge mobility, and improved passivation of the buried interface between HTMs and perovskite. As a result, PSCs employing **CF3‐mF** and **CF3‐oF** gave impressive PCEs of 23.41 and 24.13%, respectively. In addition, the large‐area (1.00 cm^2^) PSCs based on **CF3‐oF** achieved a PCE of 22.31%. Moreover, the PSCs devices with **CF3** series HTMs exhibited excellent long‐term stability under different conditions.

## Graphical abstract

A series of dithienopyran (DTP)‐based HTMs are reported by substituting different functional groups, which are applied for high‐performance perovskite solar cells (PSCs). The trifluoromethyl‐substituted **CF3‐oF** exhibits remarkable PCEs in both small‐area (0.09 cm^2^, 24.13%), and large‐area (1.00 cm^2^, 22.31%) devices, with good stability owing to its superior hole transport, film formation properties, and hydrophobicity.

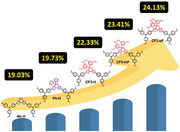

## 1 Introduction

Perovskite solar cells (PSCs) have aroused extensive attention in the field of new renewable energy technology owing to their progress in terms of photoelectric performance, thermal stability, cost‐effective fabrication process, and power conversion efficiency (PCE) in recent years.^[^ ^1^ ^]^ In particular, the PCE of perovskite‐based devices has reached from the initial 3.8% in 2009 to a certified 26.1% in 2024, which is almost comparable to that of commercial silicon‐based solar cells.^[^ ^2^ ^]^ Such progress in PSCs can be attributed to efforts in architectural designs, compositional modulations, and modifications in charge‐carrier transport layers.^[^ ^3^ ^]^ Among them, the hole‐transport material (HTM) plays a vital role in the performance and stability of PSCs, as it not only extracts and transports the photogenerated electrons but also protects the perovskite layer from moisture/oxygen.^[^ ^4^ ^]^ Up to now, most n‐i‐p‐structured PSCs with relatively high PCE are based on 2,2′,7,7′‐tetrakis‐(*N*,*N*‐di‐*p*‐methoxyphenylamine)‐9,9′‐spirobifluorene (spiro‐OMeTAD) as the HTM. Unfortunately, the large‐scale application and commercialization of spiro‐OMeTAD are limited by its tedious synthesis process, complex purification procedures, high cost, and inadequate hole mobility and conductivity.^[^ ^5^ ^]^

To address the above issues in using spiro‐OMeTAD, alternative small‐organic molecules have been actively developed as HTMs in PSC fabrication, including thiophene‐based molecules,^[^ ^6^ ^]^ spiro‐type materials,^[^ ^7^ ^]^ carbocyclic derivatives,^[^ ^8^ ^]^ and so forth. To serve as an effective HTM, several requirements must be met, such as proper energy level, high hole‐transport property, uniform film morphology on top of perovskite, and the ability to passivate defects on the perovskite surface. To enhance hole mobility and passivate defects in perovskite films, incorporating heteroatoms (such as oxygen, fluorine, and sulfur) into the structure of HTM is an effective strategy.^[^ ^9^ ^]^ In particular, thiophene‐fused molecules have been widely explored as the promising HTMs to replace spiro‐OMeTAD for their superior charge‐transporting ability as well as their potential to passivate the defects in perovskite and reduce radiative recombination via S‐Pd/I interaction. For example, benzo[1,2‐b:4,5‐b′]dithiophene (BDT), dibenzo[b,d]thiophene (DBT), and dithieno[3,2‐b:2′,3′‐d]pyrrole (DTP)^[^ ^10^ ^]^ cores have been employed to construct high‐performance PSCs. Very recently, our groups developed a series of HTMs based on the cyclopenta[2,1‐b:3,4‐b’]dithiophene (CPDT) central core featuring multiple triphenylamine (TPA) arms for PSCs and achieved high photovoltaic performance, with PCE from 17.59% to 23.31%.^[^ ^11^ ^]^ These results indicated the potential of sulfur‐rich planar cores as one factor to become high‐performance HTMs.

**Figure 1:**
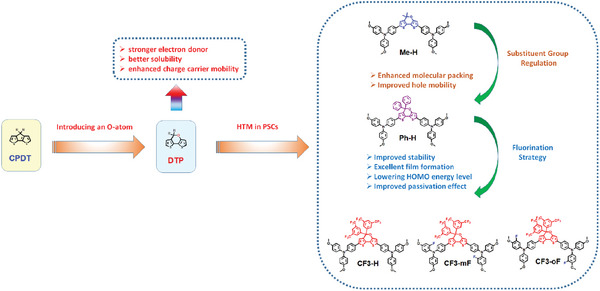
Schematic of molecular design strategy and chemical structures of **Me‐H**, **Ph‐H**, and **CF3‐** series HTMs.

Compared to the CPDT unit, the coplanar heterocyclic ring DTP features an electron‐donating oxygen atom in its pyran ring, which makes DTP a stronger electron donor (**Figure** **1**). In addition, some DTP‐based molecules were shown to exhibit higher hole mobility, better solubility, and more favorable thin film morphology compared to their CPDT counterpart, as demonstrated by Yang's group.^[^ ^12^ ^]^ As a result, the DTP unit has been widely considered a valuable building block in various optoelectronic devices including organic photovoltaics (OPVs) and dye‐sensitized solar cells (DSSCs).^[^ ^13^ ^]^ Thus, DTP‐based HTMs are anticipated to synergistically realize high hole mobility, uniform morphology, and enhanced interfacial passivation in PSCs. However, there are very limited examples of DTP‐containing HTM in PSC applications so far,^[^ ^14^ ^]^ its potential in this regard needs to be explored. On the other hand, functional groups containing fluorine atoms have been proven useful in constructing high‐performance HTMs for PSC devices, as the fluorinated HTM molecules provide additional coordination sites for Pb^2+^ via Lewis acid‐base interaction, thereby passivating defect sites at the perovskite surface and suppressing nonradiative recombination at the perovskite/HTM interface. Meanwhile, the molecular framework with highly electronegative fluorine atoms exhibits a deeper highest occupied molecular orbital (HOMO) energy level compared with the non‐fluorinated analog. This is expected to enhance the open‐circuit voltage (*V*_oc_) value. Furthermore, the superior hydrophobicity of fluorine atoms is beneficial to protecting the perovskite layer from moisture for the long‐term stability of PSCs. Therefore, incorporating fluorine atoms into π‐conjugated backbones is an effective design strategy for enhancing both the photovoltaic performance and stability of PSC devices.

Herein, we designed and synthesized five novel organic HTMs with a DTP backbone featuring triarylamine moieties as electron donors. Various substituents, with or without fluorine atoms, were introduced at specific positions to test their effect on their properties as HTM. These molecules include a methyl‐substituted HTM (**Me‐H**), a phenyl‐substituted HTM **(Ph‐H**), and three trifluoromethyl‐substituted HTMs (**CF3‐H**, **CF3‐mF**, and **CF3‐oF**), as illustrated in Figure 1. As it turns out, the change of the substituent group from methyl group in **Me‐H** to phenyl group in **Ph‐H** effectively and finely tunes molecular stacking behavior, leading to smoother thin film morphology, higher hole mobility, and higher PCE value when utilized as HTM in n‐i‐p PSC fabrication. As a result, the **Ph‐H**‐based device delivered a PCE of 19.73%, which is higher than that of the **Me‐H**‐based device (PCE = 19.03%). With four trifluoromethyl groups on the two phenyl substituents on the pyran unit in **Ph‐H** to give **CF3‐H**, more suitable energy level, better hole‐extraction ability, higher hole mobility, and smoother film morphology were observed compared with the one without trifluoromethyl groups (**Ph‐H**), thereby leading to a dramatic enhancement in PCE up to 22.33% and with negligible hysteresis for device with **CF3‐H** as the HTM. Furthermore, with additional fluorine atoms introduced at different positions on the triphenylamine groups on the phenyl rings (**CF3‐mF** and **CF3‐oF)**, the electronic structure, energy level, film‐forming property, passivation effect, and hydrophobicity were further tuned. As a result, the device with **CF3‐oF** delivered an excellent PCE of 24.13%, higher than that of the isomer **CF3‐mF** (23.41%). Meanwhile, a large‐area (1.0 cm^2^) device with **CF3‐oF** was also fabricated and a decent PCE of 22.31% was obtained. Moreover, PSCs with the series of trifluoromethyl‐substituted HTMs exhibited excellent stability under different conditions compared to other analogs. These results show that modifying the core unit and peripheral groups with fluorine atoms is advantageous in achieving highly efficient and stable PSCs.

## 2 Results and Discussion

**Scheme 1:**
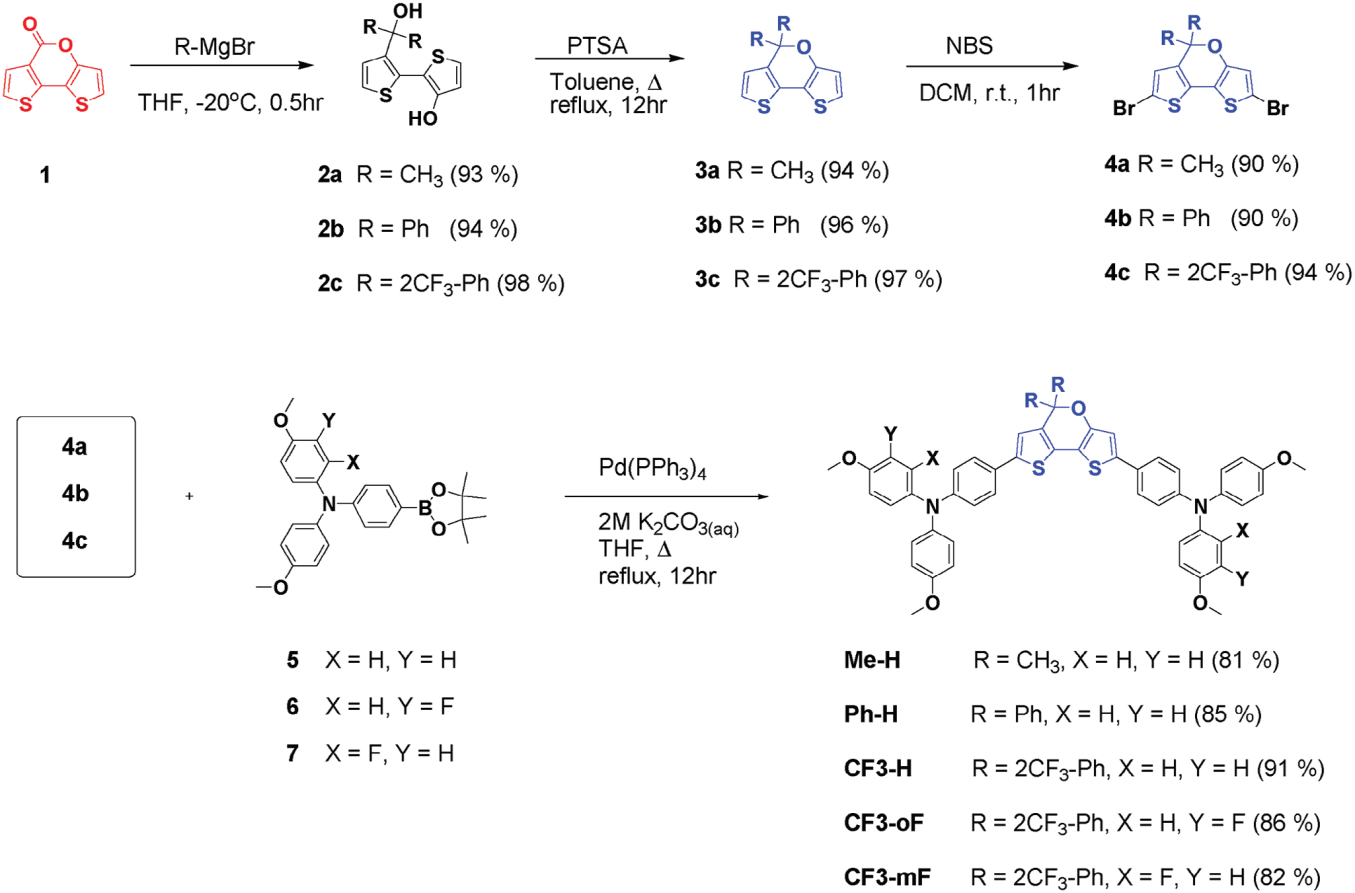
Synthetic procedures for **Me‐H**, **Ph‐H**, and **CF3‐**series HTMs.

The details of synthetic routes of **Me‐H**, **Ph‐H**, and **CF3‐**series are shown in **Scheme** **1**. Compounds **2** were obtained using different Grignard reagents. Subsequently, the ring‐closure reaction was carried out through dehydration in acidic conditions to afford the compounds **3**. Then the bromination reaction of the compounds **3** with *N*‐bromosuccinimide (NBS) afforded the intermediates **4**. Finally, the five target compounds **Me‐H**, **Ph‐H**, **CF3‐H**, **CF3‐mF**, and **CF3‐oF** were obtained via two‐fold Suzuki cross‐coupling reactions in high yields. The details of their characterizations using NMR and high‐resolution mass analysis are provided in the Supporting Information (Figures S1–S48, Supporting Information). As shown in Tables S1–S15 (Supporting Information), the synthetic cost analysis of the new HTMs was estimated to be 47 to 75 USD/g, which is lower than that of spiro‐OMeTAD (170 to 475 USD/g).^[^ ^15^ ^]^ To gain insight into the thermal properties of **Me‐H**, **Ph‐H**, and **CF3‐**series, thermogravimetric analysis (TGA) and differential scanning calorimetry (DSC) measurements were carried out and the corresponding data are summarized in **Table** **1**. As displayed in Figure S49a (Supporting Information), all the new HTMs present excellent thermal stability, with high decomposition temperatures (T*_d_*, the temperature with 5% weight‐loss) in the range of 346—407 °C, which is essential for device operation. During the second scan of DSC investigations (10 °C min^−1^), the DSC cycles revealed that all the HTMs are amorphous with a glass transition temperature at 118, 116, 129, 128, and 128 °C for **Me‐H**, **Ph‐H**, **CF3‐H**, **CF3‐mF**, and **CF3‐oF**, respectively. However, the DSC of **Me‐H**, **Ph‐H**, and the **CF3**‐series do not show any melting temperature (Tm). The T_g_ values for the **CF3** series are slightly higher than that of spiro‐OMeTAD (125 °C),^[^ ^16^ ^]^ suggesting improved morphological stability for these materials. The amorphous nature of **Me‐H**, **Ph‐H**, and **CF3‐**series films was verified by powder X‐ray diffraction (PXRD) measurements. As shown in Figure S50 (Supporting Information), the films of all the new HTMs were essentially amorphous, suggesting that they may give morphologically more stable films.

**Table 1:** Photophysical, electrochemical properties and thermal data of **Me‐H**, **Ph‐H**, and **CF3‐**series and spiro‐OMeTAD.

HTM	*λ*_abs_ [nm] [*ε *× 10^−4^ M^−1^ cm^−1^] ^a^ ^)^	*λ*_f_ [nm]	*E*_HOMO_ [eV] ^b^ ^)^	*E*_0‐0_ [eV] ^c^ ^)^	*E*_LUMO_ (eV) ^d^ ^)^	*E*_HOMO_ [eV] ^e^ ^)^	*E*_0‐0_ [eV] ^e^ ^)^	*E*_LUMO_ [eV] ^e^ ^)^	T*_g_* [^o^C] ^f^ ^)^	T*_d_* [^o^C] ^f^ ^)^
Me‐H	449 (5.9)	512	−5.23	2.51	−2.72	−4.25	2.90	−1.35	118	384
Ph‐H	453 (6.1)	517	−5.16	2.50	−2.66	−4.21	2.80	−1.41	116	388
CF3‐H	462 (5.8)	530	−5.36	2.46	−2.90	−4.52	2.77	−1.75	129	346
CF3‐mF	452 (5.9)	516	−5.40	2.48	−2.92	−4.54	2.76	−1.78	128	351
CF3‐oF	457 (5.9)	523	−5.42	2.52	−2.90	−4.63	2.80	−1.83	128	345
spiro‐OMeTAD	389 (18.4)	428	−5.15	3.02	−2.13	−4.21	3.60	−0.61	125^g^ ^)^	422^g^ ^)^

^a)^ Maxima of the absorption bands in THF solution;

^b)^ Determined by differential pulse voltammetry (DPV);

^c)^ The value of *E*_0‐0_ obtained from the onset of absorption spectra;

^d)^ Energy of the LUMO of the compounds estimated by *E*_HOMO_+*E*_0‐0_;

^e)^ Values calculated at DFT/B3LYP/6‐31G(d,p) level;

^f)^ Glass transition (T*_g_*) and decomposition (T*_d_*) temperatures observed from TGA and DSC, respectively;

^g)^ These dates have been reported in reference.^[^ ^16^ ^]^

**Figure 2:**
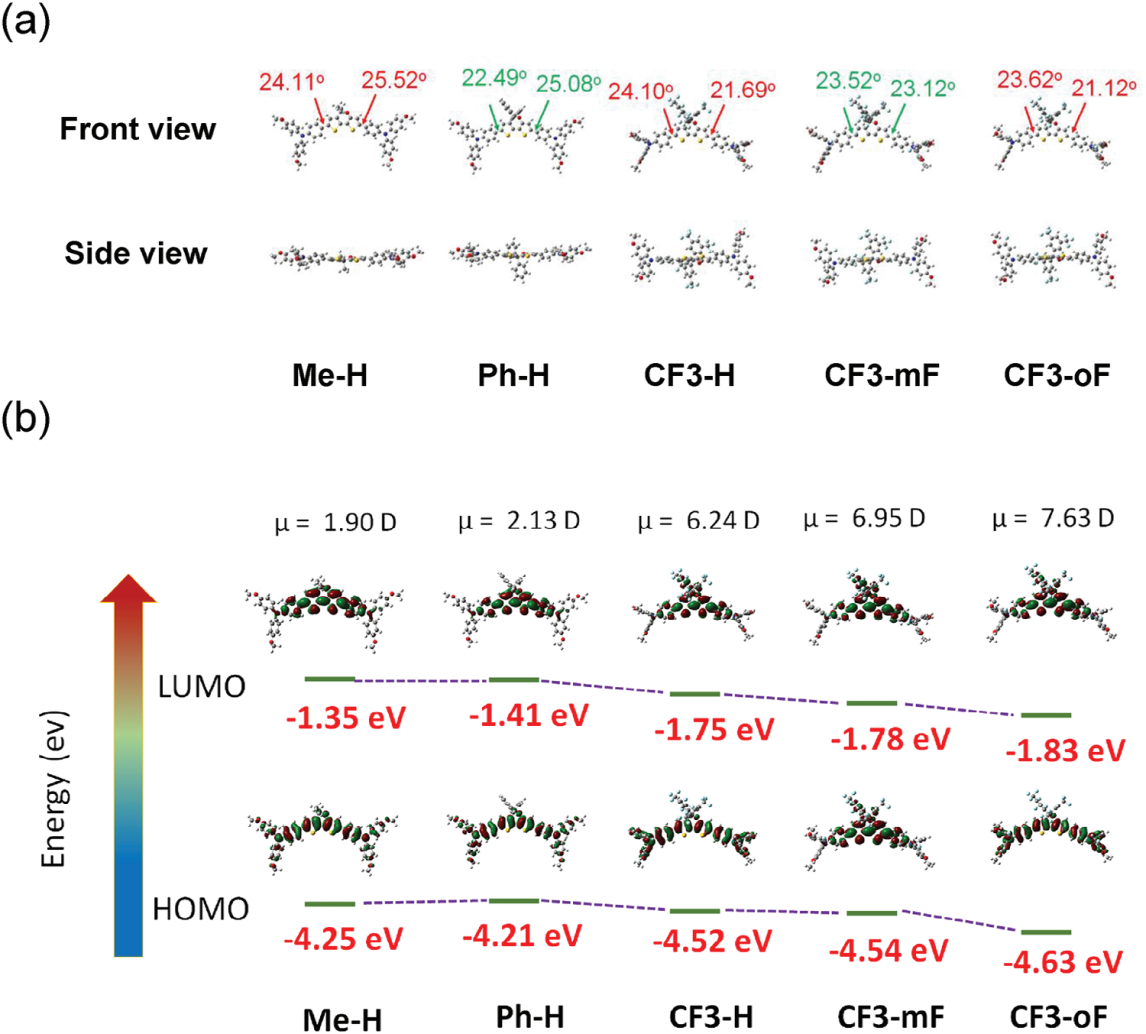
a) Top view and side view of the optimized structures for **Me‐H**, **Ph‐H**, and **CF3‐**series obtained from DFT calculations. b) The electron densities of HOMO and LUMO levels are calculated by DFT at the B3LYP/6‐31(d,p) level.

Density functional theory (DFT) calculations at B3LYP/6‐31G (d,p) level were performed to obtain optimized molecular geometries, energy levels of frontier molecular orbitals, and dipole moments of **Me‐H**, **Ph‐H**, and **CF3‐**series. As shown in **Figure** **2**a, the planar 5*H*‑dithieno[3,2‑*b*:2′,3′‑*d*]pyran (DTP) are introduced as the central core with the propeller‐like TPA units as the electron donors. In addition, it can be seen that there are no significant differences in the electronic distribution of the highest‐occupied molecular orbitals (HOMOs) and the lowest‐unoccupied molecular orbitals (LUMOs) for **Me‐H**, **Ph‐H**, and **CF3**‐series (Figure 2b). The HOMOs of all the new HTMs are mainly distributed over its π‐conjugated backbone, while their LUMOs are primarily located on the central DTP moiety and their adjacent phenyl rings. Furthermore, the calculated HOMOs/LUMOs energy levels are ‐4.25/‐1.35, ‐4.21/‐1.41, ‐4.52/‐1.75, ‐4.54/‐1.78 and ‐4.63/‐1.83 eV for **Me‐H**, **Ph‐H**, **CF3‐H**, **CF3‐mF**, and **CF3‐oF**, respectively. As anticipated, the introduction of electron‐withdrawing trifluoromethyl functionality can greatly down‐shift both HOMO/LUMO energy levels, which is beneficial to reducing current leakage and open‐circuit voltage (*V*_oc_) loss owning to the lower offset between the valence band of perovskite and HOMO energy levels of HTMs. Moreover, trifluoromethyl‐substituted **CF3**‐series exhibited an increased dipole moment (6.24–7.63 D) compared with that of **Me‐H** (1.90 D) and **Ph‐H** (2.13 D), presumably due to the strong donor‐acceptor (D‐A) character. The larger dipole moment of the **CF3**‐series may strengthen the intermolecular interaction, which is also beneficial for stronger packing and facilitates hole extraction and transport.

**Figure 3:**
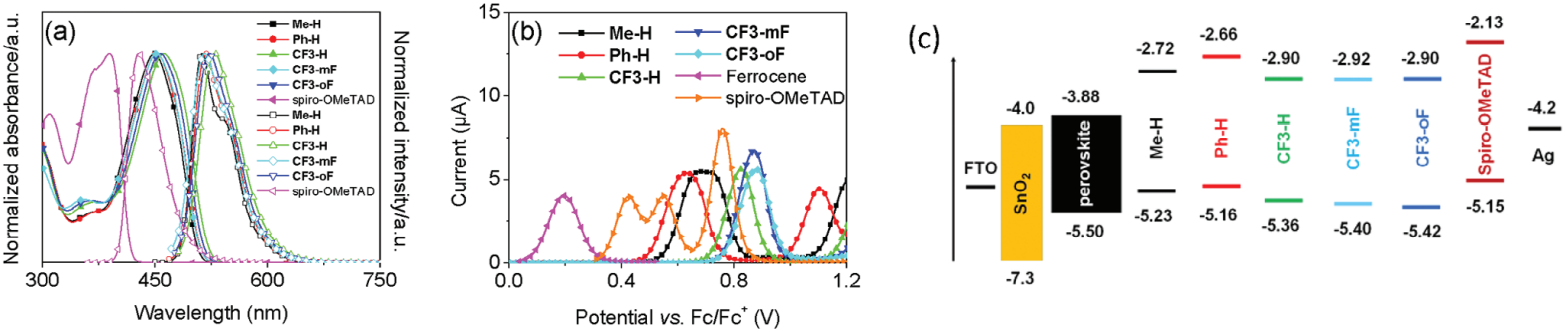
a) Normalized absorption and photoluminescence spectra of spiro‐OMeTAD, **Me‐H**, **Ph‐H**, **CF3‐**series in THF solution. b) Differential pulse voltammetry (DPV) of ferrocene, spiro‐OMeTAD, **Me‐H**, **Ph‐H**, and **CF3‐**series in THF solution. c) Band energy alignment of **Me‐H**, **Ph‐H**, and **CF3‐**series.

The optical properties of **Me‐H**, **Ph‐H**, and **CF3**‐series were examined by UV–vis and photoluminescence (PL) spectra in THF solution. As shown in **Figure** **3**a, all five molecules have similar absorption spectra, with two peaks located at ≈290 nm, assignable to the 𝜋–𝜋^*^ transition of the conjugated system while the other strong absorption bands in the range of 425—520 nm can be attributed to the intramolecular charge transfer (ICT) transitions from the triphenylamine (TPA) moieties to the central DTP moiety. **CF3‐H** shows a larger red shift of the absorption than its congeners (**Me‐H** and **Ph‐H**), indicating that the former with trifluoromethyl substituents on the phenyl ring has a stronger ICT character. Moreover, it can be seen that the absorption maxima of **CF3‐oF** and **CF3‐mF** are slightly blue‐shifted by 5 and 10 nm, respectively, compared with that of **CF3‐H**, presumably due to the strong electronegativity of fluorine atoms on the aromatic rings of TPA groups lower the HOMO level more than the LUMO level. The absorption spectra of **Me‐H**, **Ph‐H**, and **CF3**‐series were simulated by the time‐dependent DFT (TD‐DFT) calculation using the Gaussian 16 program. As shown in Figure S51 (Supporting Information), all the new HTMs exhibit weak absorption bands at ≈280—310 nm, arising from the 𝜋–𝜋^*^ transition between the conjugated units, and strong absorption bands at ≈450—600 nm, arising from the ICT absorption, which is in good agreement with the experimental results. In addition, the maximum emission bands of **Me‐H**, **Ph‐H**, and **CF3**‐series are found at 505—520 nm. Furthermore, the energy gaps (*E*_g_) of these molecules are calculated based on the intersection of UV absorption and PL spectra and are summarized in Table 1.

Cyclic voltammetry (CV) and differential pulse voltammetry (DPV) measurements were conducted to investigate the redox behaviors of **Me‐H**, **Ph‐H**, and **CF3‐**series in THF solution (Figures 3b and S52, Supporting Information), and the detailed electrochemical data are shown in Table 1. As shown in Figure 3b, the calculated HOMO levels of **Me‐H**, **Ph‐H**, **CF3‐H**, **CF3‐mF**, and **CF3‐oF** are found to be −5.23, −5.16, −5.36, −5.40, and −5.42 eV, respectively. These DPV results agree well with the DFT calculation results (Figure 2b). Moreover, the two trifluoromethyl substituents introduced on the pyran ring effectively lower the HOMO energy levels of **CF3‐H**, **CF3‐mF**, and **CF3‐oF** compared to that without trifluoromethyl substituent (**Ph‐H**). This would help to increase the *V*_oc_ of the PSCs. In addition, the LUMO energy levels of **Me‐H**, **Ph‐H**, and **CF3‐**series were calculated to be in the range of −2.72 to −2.92 eV based on the optical band gaps and HOMO energy levels. They are higher than the conduction band (CB) of the perovskite and thus could inhibit the electron transfer from perovskite to metal counter electrode. The schematic band energy level alignment of PSCs is depicted in Figure 3c.

**Figure 4:**
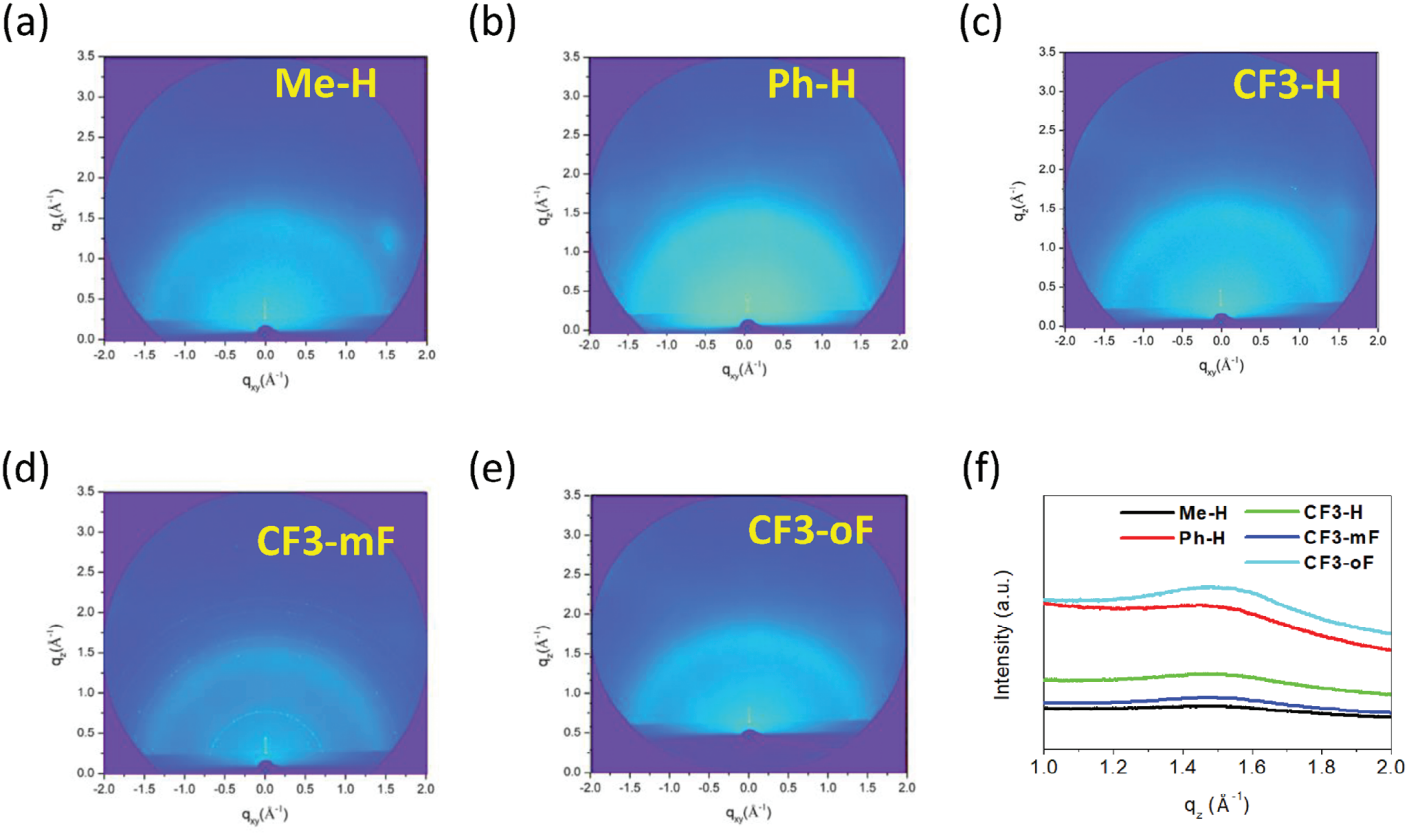
a) 2D GIWAXS patterns of (a) **Me‐H** film, b) **Ph‐H** film, c) **CF3‐H** film, d) **CF3‐mF** film, e) **CF3‐oF** film, and f) Out‐of‐plane line cuts of the of **Me‐H**, **Ph‐H**, **CF3‐H**, **CF3‐mF**, and **CF3‐oF**.

The hole‐only device with a configuration of ITO/PEDOT:PSS/HTM/Al was used to measure the hole mobilities of these molecules by the space‐charge‐limited current (SCLC) method, and the results are shown in Figure S53 (Supporting Information). The hole mobilities of the doped **Me‐H**, **Ph‐H**, **CF3‐H**, **CF3‐mF**, and **CF3‐oF**‐based devices were calculated to be 2.47 × 10^−4^, 2.77 × 10^−4^, 3.85 × 10^−4^, 4.75 × 10^−4^, and 6.11 × 10^−4^ cm^2^V^−1^ s^−1^, respectively. It is noteworthy that the **CF3**‐series show higher hole mobilities compared with the others and the doped spiro‐OMeTAD (3.56 × 10^−4^ cm^2^V^−1^ s^−1^). This can be ascribed to the effective charge transfer process through the D‐A structure, which strengthens the intermolecular interaction, and thus has the potential to give better photovoltaic performance. The results are consistent with the dipole moment calculation above. Furthermore, grazing‐incidence wide‐angle X‐ray scattering (GIWAXS) was employed to investigate the molecular stacking of the **Me‐H**, **Ph‐H**, and **CF3‐**series. As illustrated in **Figure** **4**, all the HTMs exhibit stronger (010) diffraction peaks in the out‐of‐plane direction with preferred face‐on orientation, which is the favored orientation for charge transport in the device. The (010) peaks of **Me‐H**, **Ph‐H**, **CF3‐H**, **CF3‐mF**, and **CF3‐oF** films appear at q_z_ = 1.42, 1.42, 1.46, 1.47, and 1.48 Å^−1^, corresponding to a π–π stacking distance of 4.32, 4.32, 4.30, 4.29, and 4.26 Å respectively. Therefore, trifluoromethyl substituent on the DTP core can result in larger dipole moments and effectively strengthen the molecular packing, benefitting the charge transport for higher short‐circuit current density (*J*_sc_) and fill factor (FF) in PSCs.

**Figure 5:**
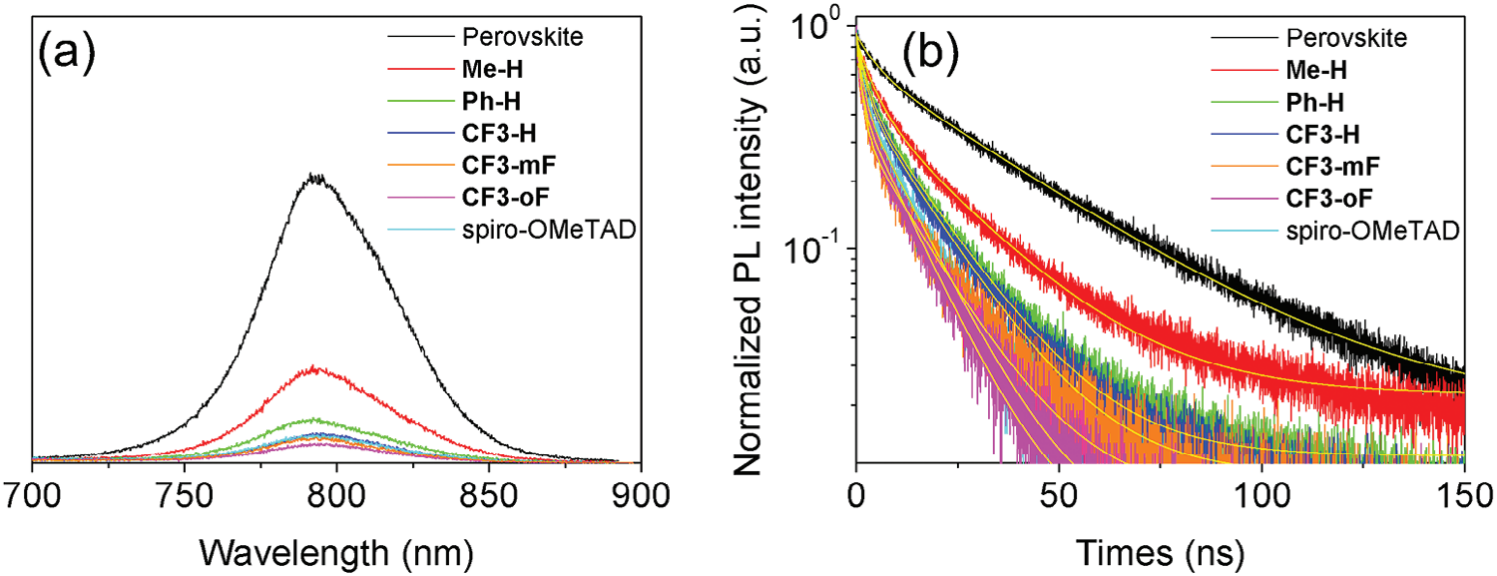
a) Steady‐state photoluminescence spectra and b) Time‐resolved photoluminescence (TRPL) curves film with/without HTMs.

To analyze the hole transfer dynamics at the interface between the perovskite and the HTM, steady‐state photoluminescence (PL) spectra and time‐resolved photoluminescence (TRPL) decay measurements were performed and the results are shown in **Figure** **5**. Compared with the pristine perovskite, the newly‐developed HTM films on perovskite show a significantly decreased PL intensity, and the order of the intensity quenching was **CF3‐oF** > **CF3‐mF** > **CF3‐H** > **Ph‐H** > **Me‐H**, which is consistent with the trend of hole mobility. The strongest PL quenching was observed for the perovskite film covered with **CF3‐oF** film, indicating that **CF3‐oF** exhibits a better hole extraction ability at the perovskite/**CF3‐oF** interface. As shown in Figure 5b, the perovskite layers covered with **Me‐H**, **Ph‐H**, and **CF3‐**series layers all present an obvious decay, and the detailed decay parameters are shown in Table S21 (Supporting Information). It is found that the PL lifetimes (τ) decrease from 24.21 ns for the pristine perovskite layer to 11.65, 8.76, 8.15, 5.49, and 5.41 ns for **Me‐H**, **Ph‐H**, **CF3‐H**, **CF3‐mF**, and **CF3‐oF**, respectively by fitting with a bi‐exponential mode. This demonstrates that fluorinated HTMs improved hole extraction and transport between the perovskite/HTM interface. Furthermore, the **CF3‐oF**‐coated perovskite film has the shortest PL lifetime, indicating the most efficient charge carrier extraction owing to favorable energy level alignment.

**Figure 6:**
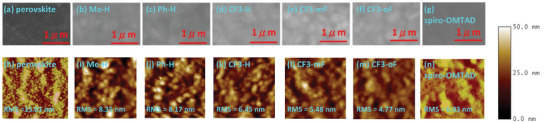
The top‐view SEM images a–g) and AFM images h–n) of perovskite film and perovskite/HTMs.

A uniform film morphology is of great significance for the collection and transport of charge carriers and even the stability of the PSC device. According to scanning electron microscopy (SEM) images of the perovskite films with/without an HTM layer, all of the **Me‐H**, **Ph‐H**, and **CF3‐**series films exhibited uniform and pinhole‐free coverage on the perovskite film (**Figures** **6**a–g). In addition, an atomic force microscope (AFM) was used to evaluate the surface morphology of perovskite film coated with various HTM films. As shown in Figure 6h–n, all of them present a smooth and homogenous surface with a root‐mean‐square (RMS) roughness of 4.77–8.35 nm, which is smaller than that (RMS = 15.01 nm) of pristine perovskite film. Meanwhile, trifluoromethyl‐substituted **CF3**‐series exhibit smaller RMS values as compared with the **Me‐H** and **Ph‐H** films, suggesting better film quality of **CF3**‐series. The RMS roughness of perovskite films covered with **CF3‐H**, **CF3‐mF**, and **CF3‐oF** are 6.45, 5.48, and 4.77 nm, respectively, suggesting that **CF3‐oF** exhibits better film‐forming ability. It is beneficial to efficient interfacial contact and carrier transportation.

**Figure 7:**
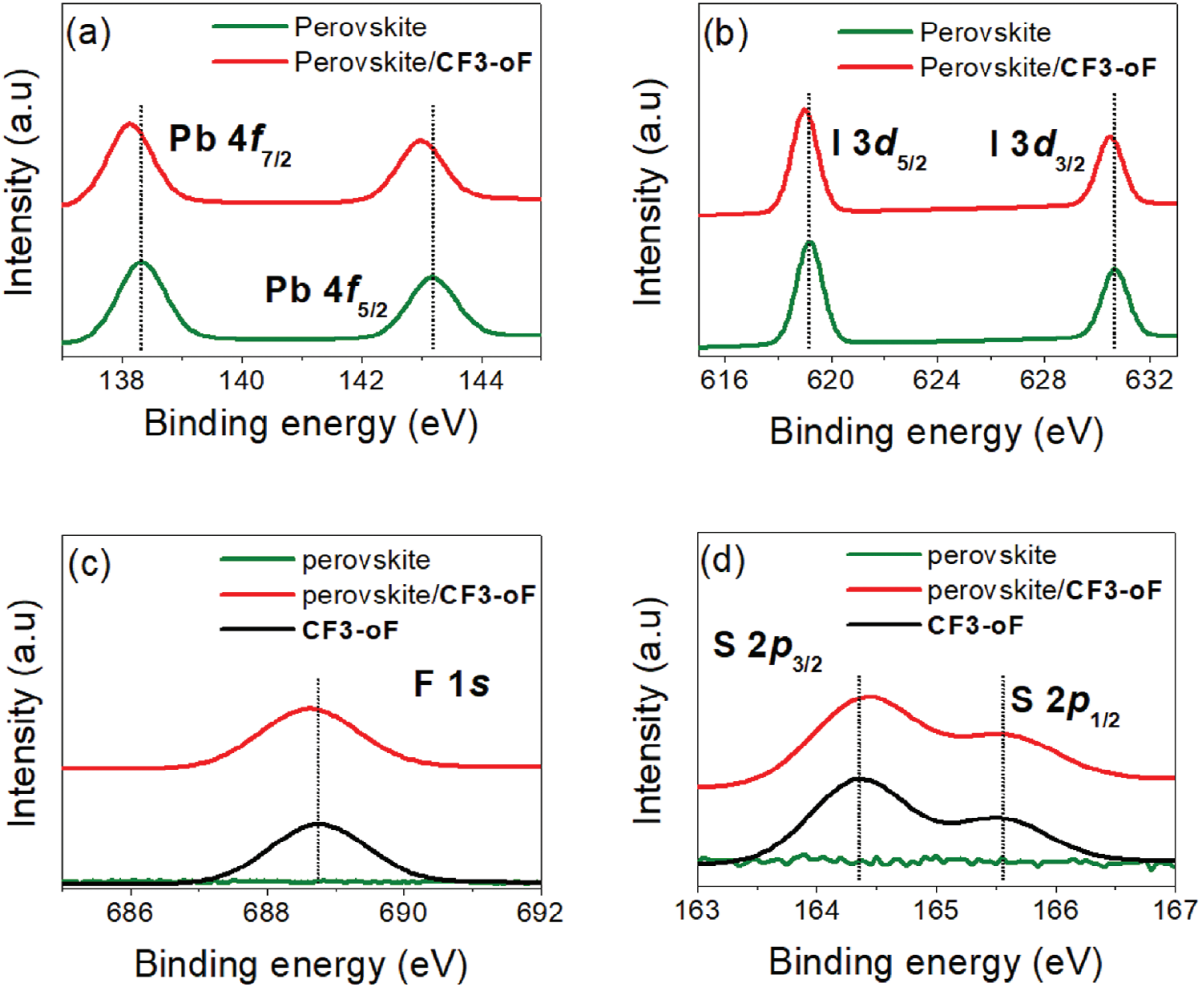
XPS signals of Pb 4f, I 3d, F 1s, and S 2p from a pristine **CF3‐oF** film and a **CF3‐oF** coated perovskite film.

X‐ray photoelectron spectra (XPS) measurements were carried out to elucidate the interaction between the perovskite films and various HTMs (**Figure** **7**). The XPS spectra of Pb 4f and I 3d core level showed two main peaks at 143.20, 138.3 eV and 630.7, 619.2 eV for pristine perovskite film, which are assigned to the binding energies of Pb 4f_5/2_, Pd 4f_7/2_ and I 3d _3/2_, I 3d _5/2_, respectively. Compared with pristine perovskite film, the characteristic peaks of Pb 4f and I 3d for perovskite/**Me‐H**, perovskite/**Ph‐H**, and perovskite/**CF3‐H** bilayer shifted to higher binding energies (Figures S54–S56, Supporting Information), while that for perovskite/**CF3‐mF** and perovskite/**CF3‐oF** bilayer shifted to lower binding energies, suggesting a strong interaction between the **Me‐H**, **Ph‐H**, and **CF3‐**series layers and perovskite film and thus effective passivation effect of **Me‐H**, **Ph‐H**, and **CF3‐**series HTMs on the perovskite film (Figures 7a,b and S56a,b, Supporting Information). However, the fact that the two typical peaks of Pb 4f and I 3d shift to lower binding energies for **CF3‐mF** and **CF3‐oF‐**covered perovskite compared with other HTM might be attributed to two reasons, 1) coordination of Pb dangling bonds with **CF3‐mF** and **CF3‐oF** released Pb─I bonding on the surface;^[^ ^17^ ^]^ 2) the non‐covalent interaction between the F atoms on the TPA groups and I atom. Overall, these results imply that **CF3‐mF** and **CF3‐oF** can further inhibit ion migration and enable long‐term stability. Moreover, it is found that the F 1s peaks of **CF3‐**series‐covered perovskite films shifted toward lower binding energies in comparison with pristine perovskite film (Figure 7c), indicating **CF3**‐series has a stronger binding on the perovskite surface to passivate the trap states and improve the crystallinity of perovskite films.^[^ ^18^ ^]^ These results indicate that the fluorination strategy could enhance defects passivation between HTM and the perovskite surface. In particular, the XPS experiments of F 1s revealed a larger shift in the perovskite/**CF3‐mF** and perovskite/**CF3‐oF** layers compared to the perovskite/**CF3‐H** layer, suggesting a stronger interaction between **CF3‐mF** and **CF3‐oF** and perovskite, presumably because of the additional F atoms on the TPA moieties. Furthermore, we found that the S 2p XPS core spectra of pure **Me‐H**, **Ph‐H**, and **CF3‐**series films consist of two main peaks. These two peaks shift toward higher binding energy after coating on the perovskite films, indicating a strong interaction between **Me‐H**, **Ph‐H**, and **CF3‐**series layers and perovskite. These results implied that all the **Me‐H**, **Ph‐H**, and **CF3‐**series molecules can provide more efficient passivation.

**Figure 8:**
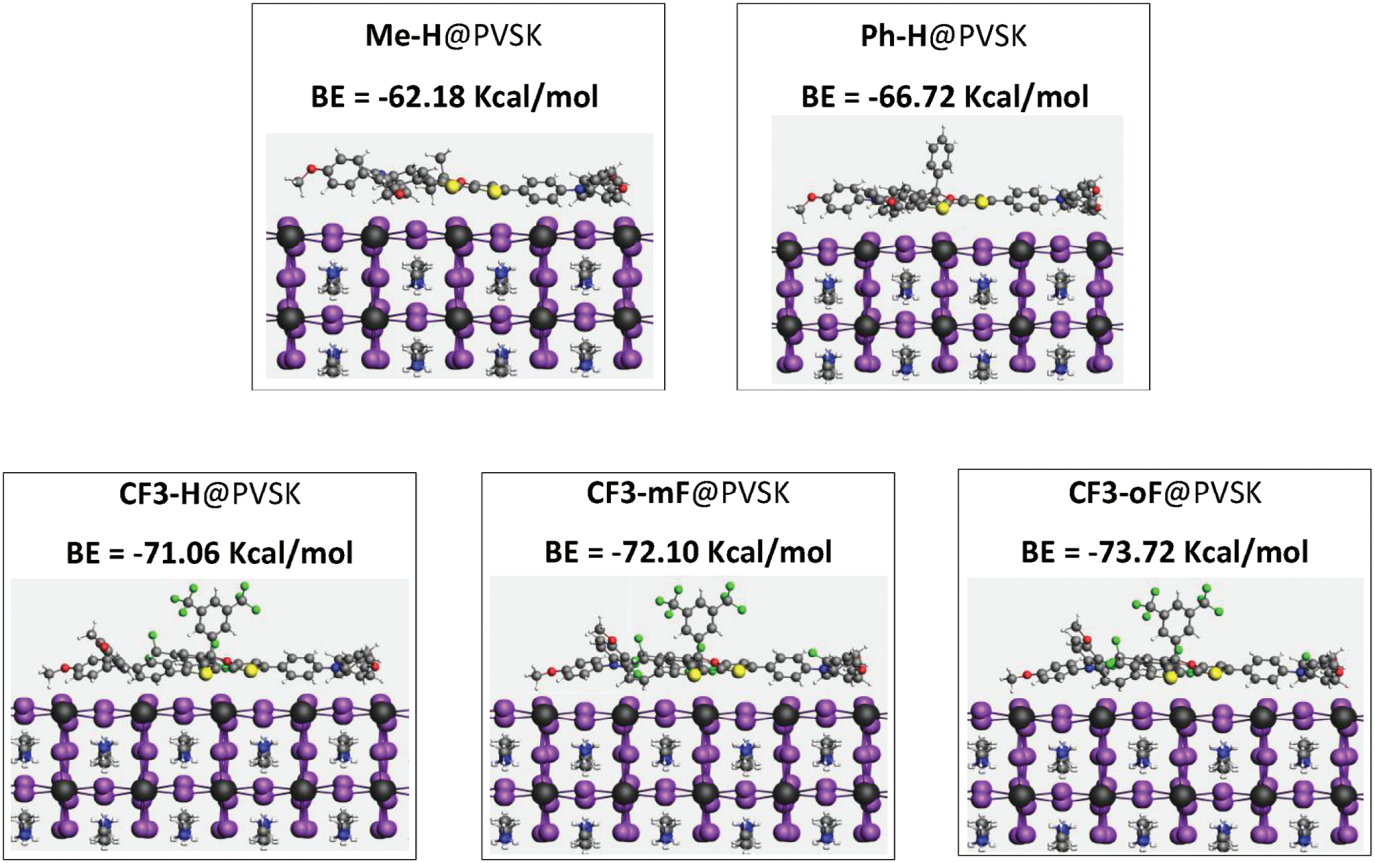
DFT‐simulated stacking patterns and binding energy (BE) of different HTMs on top of a perovskite surface.

To investigate the interaction between different HTM molecules and perovskite surfaces, the binding energy was calculated by DFT calculations. One molecule was placed on top of the perovskite surface and the system was allowed to reach equilibrium. The energy‐minimized structures were obtained. As illustrated in **Figure** **8**, **Me‐H**, **Ph‐H**, and **CF3‐**series molecules exhibited almost flatness on the perovskite surface, different from the free molecule presumably due to the binding interaction of sulfur atoms with the Pd sites of perovskite. In addition, the binding energies between perovskite and HTMs were −62.18, −66.72, −71.06, −72.10 and −73.72 kcal mol^−1^, respectively for **Me‐H**, **Ph‐H**, **CF3‐H**, **CF3‐mF**, and **CF3‐oF**, suggesting the stronger interaction of the fluorinated HTMs with perovskite, indicating the more effective passivation and hole extraction ability of **CF3**‐series HTMs.

**Figure 9:**
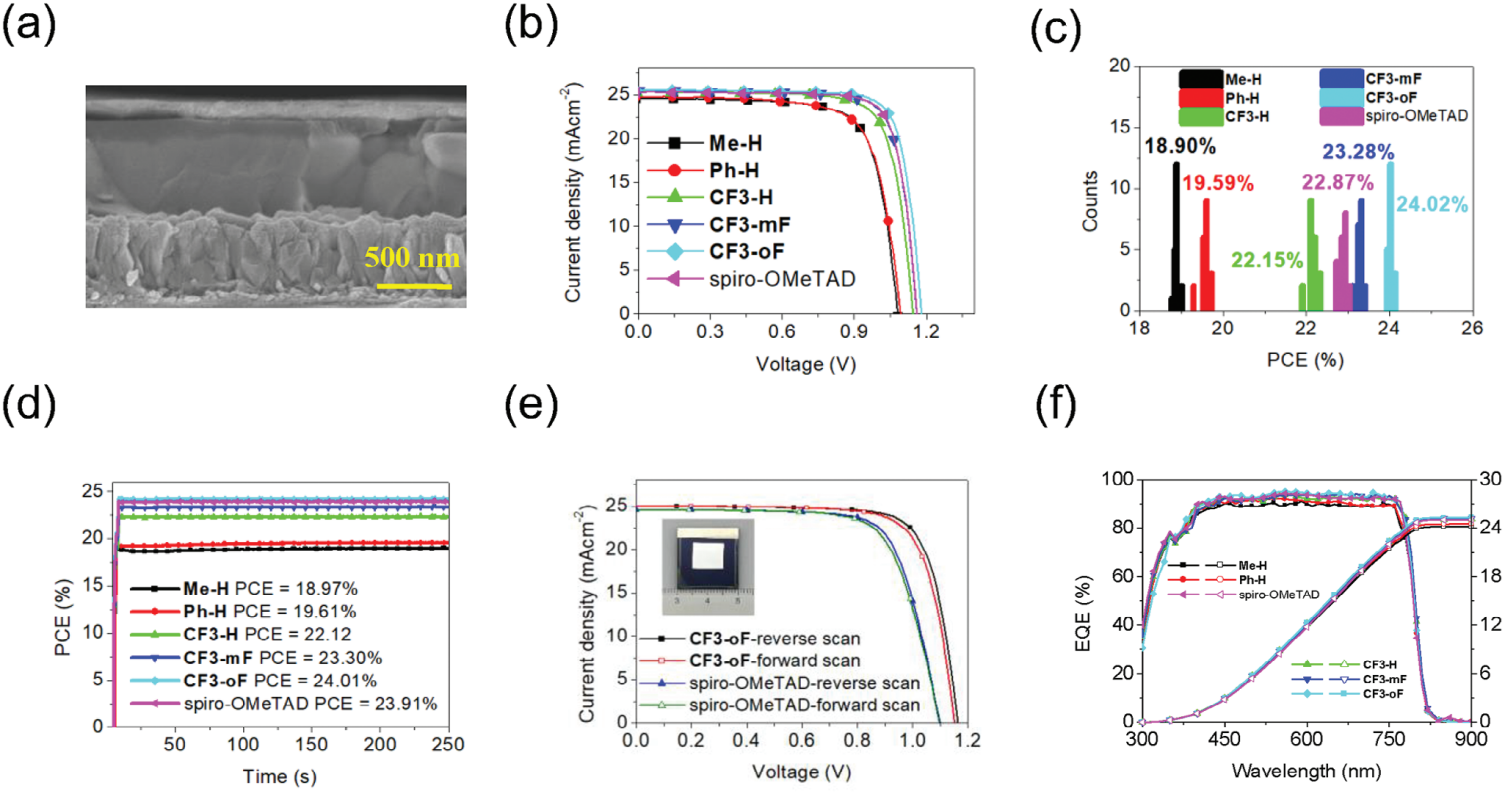
a) Cross‐sectional SEM image of the PSC device with **CF3‐oF** as HTM. b) *J*–*V* characteristics of the optimal PSC devices with **Me‐H**, **Ph‐H**, and **CF3‐**series and spiro‐OMeTAD as HTMs. c) the statistical distribution of PCEs from 20 independent devices. d) Stable output power for **Me‐H**, **Ph‐H**, and **CF3‐**series as well as spiro‐OMeTAD‐based PSCs. e) *J*–*V* curves of large‐area (aperture area of 1.00 cm^2^) PSCs based on **CF3‐oF** and spiro‐OMeTAD. The inset is a picture of the large‐area PSC. f) The EQE spectra and the integrated current of the PSCs with **Me‐H**, **Ph‐H**, **CF3‐**series, and spiro‐OMeTAD.

Based on the encouraging properties observed above, the PSCs with a conventional configuration of FTO/SnO_2_/Cs_0.05_MA_0.2_FA_0.75_Pb(Br_0.05_I_0.95_)_3_/HTM/Ag were fabricated to evaluate the device performance of **Me‐H**, **Ph‐H**, and **CF3‐**series as HTMs. Detailed information on device fabrication is described in the Supporting information. The device with spiro‐OMeTAD was fabricated as a reference for comparison. All of the HTMs were doped with 4‐tert‐butylpyridine (*t*‐BP) and lithium bis(trifluoromethanesulfonyl)imide (Li‐TFSI) as additives. **Figure** **9**a shows a representative cross‐sectional SEM image of the PSC with **CF3‐oF** as HTM. The thickness of the **CF3‐oF** and the perovskite film are ≈130 and 500 nm, respectively. Figure 9b shows the current density–voltage (*J*–*V*) characteristic curves for the PSCs with the five new HTMs as well as spiro‐OMeTAD under standard AM 1.5G irradiation at 100 mW cm^−2^ and the corresponding photovoltaic parameters are listed in **Table** **2**. The **Me‐H**‐based devices delivered a champion PCE of 19.03% under reverse scan with a *V*_oc_ of 1.08 V, a *J*_sc_ of 24.54 mA cm^−2^ and an *FF* of 71.81%, while the device with **Ph‐H** featuring two phenyl rings on the central core exhibited a higher PCE of 19.73%, with a *V*_oc_ of 1.09 V, a *J*_sc_ of 24.77 mA cm^−2^, and an *FF* of 73.07%. As compared with the **Me‐H**‐based device, the enhancement of PCE for the **Ph‐H**‐based device is mainly due to the improvement of *J*_sc_ and FF, which might be attributed to the more efficient hole extraction capacity (Figure 5), higher hole mobility (Figure S53, Supporting Information), and the better film morphology (Figure 6). It is clear that the substituent group on the 5*H*‑dithieno[3,2‑b:2′,3′‑d]pyran moity exerts great influence on the photovoltaic performance of PSCs. The results suggest that the additional two phenyl rings on **Ph‐H** reinforce intermolecular π–π stacking. Meanwhile, the device based on two trifluoromethyl‐substituted **CF3‐H** displayed a PCE of 22.33%, with a *V*_oc_ of 1.14 V, a *J*_sc_ of 25.31 mA cm^−2^, and an *FF* of 77.39%, remarkably higher than that of the counterpart **Ph‐H**‐based device. The remarkably improved PCE of the **CF3‐H** device mainly arises from the improved *V*_oc_, *J*_sc_, and *FF* owing to more matched energy level (Figure 3), higher hole mobility (Figure S53, Supporting Information), better morphology (Figure 6), and more efficient defect passivation (Figure 7). Furthermore, it was found that the introduction of fluorine atoms on the triarylamine moieties (**CF3‐mF** and **CF3‐oF**) further raised the photovoltaic performance. Thus the **CF3‐mF** cell exhibited a *V*_oc_ of 1.16 V, a *J*_sc_ of 25.49 mA cm^−2^, an *FF* of 79.18%, and a PCE of 23.41%, which are even higher than that of the reference cell with spiro‐OMeTAD (PCE of 23.03%, *V*_oc_ of 1.16, *J*_sc_ of 25.28 and *FF* of 78.54%). In comparison, the device using isomeric analog **CF3‐oF** presented the highest PCE of 24.13%, with a *V*_oc_ of 1.18 V, a *J*_sc_ of 25.51 mA cm^−2^, and an *FF* of 80.16%. The increase in *V*_oc_, *J*_sc_, *FF*, and PCE values may result from a better energy alignment between the perovskite and HTMs, higher hole mobility, hole extraction efficiency, smoother film morphology, and better interfacial contact. A fluorine atom *ortho* to the electron‐donating *p*‐methoxy group on the phenyl group (**CF3‐oF**) can improve the performance of PSCs, as also demonstrated by previous work.^[^ ^11^, ^19^ ^]^ Moreover, as shown in Figure S58 (Supporting Information) and Table 2, all of the devices exhibit negligible hysteresis with hysteresis index (HI) of 1.10, 0.51, 1.03, 1.41, 0.79, 0.87% for **Me‐H**, **Ph‐H**, **CF3‐H**, **CF3‐mF**, **CF3‐oF**, and spiro‐OMeTAD, respectively based on HI = (PCE_reverse_−PCE_forward_)/PCE_reverse_. The statistic histogram of the PSCs based on 20 devices is shown in **Figure** **9**c. Average PCEs of 18.90, 19.59, 22.15, 23.28, 24.02 and 22.87% were achieved for **Me‐H**‐, **Ph‐H**‐, **CF3‐H**‐, **CF3‐mF**‐, **CF3‐oF**‐, and spiro‐OMeTAD‐based devices, respectively, with a narrow distribution, demonstrating a good reproducibility. As shown in Figure 9d, the steady‐state power output at the maximum power point (MPP) was conducted to explore the operational stability under one sun illumination for 250s. The stabilized PCEs with **Me‐H**, **Ph‐H**, **CF3‐H**, **CF3‐mF**, **CF3‐oF**, and spiro‐OMeTAD are 18.97, 19.61, 22.12, 23.30, 24.01 and 23.91%, respectively, which confirms the efficiencies of these PSCs. In addition, large‐scale PSCs with an area of 1.0 cm^2^ containing **CF3‐oF** and spiro‐OMeTAD as HTM were fabricated. As shown in Figure 9e, the **CF3‐oF**‐based large‐area device exhibited a PCE of 22.31% with a *V*_oc_ of 1.16 V, a *J*_sc_ of 25.03 mA cm^−2^, and an *FF* of 76.94%, higher than that of a spiro‐OMeTAD‐based device (20.15%). Considering the suitable properties of these new HTMs, we further evaluate their application in devices without using dopants. The dopant‐free **Me‐H**‐, **Ph‐H**‐, **CF3‐H**‐, **CF3‐mF**‐, **CF3‐oF**‐, and spiro‐OMeTAD‐based PSCs exhibited poorer photovoltaic performance, in the range of 10.64–13.47%, under reverse scan (Figure S59, Table S22, Supporting Information), indicating that the dopants are still necessary to enhance the photovoltaic performance for these series of HTM. Furthermore, the external quantum efficiency (EQE) spectra and integrated *J*_sc_ curves were collected for the **Me‐H**‐, **Ph‐H**‐, **CF3‐H**‐, **CF3‐mF**‐, **CF3‐oF**‐, and spiro‐OMeTAD‐based devices (Figure 9f). The integrated *J*_sc_ values estimated from the corresponding spectra are 24.21, 24.52, 25.10, 25.22, 25.31, and 25.06 mA cm^−2^, respectively, which are in good agreement with the results obtained from the *J*‐*V* curves.

**Table 2:** Photovoltaic parameters extracted from *J*‐*V* measurements of PSCs based on the various HTMs.

HTM	Scan direction		*V*_oc_ [V]	*J*_sc_ [mA cm^−2^]	FF [%]	PCE_max_ [%]
Me‐H	Reverse	Best	1.08	24.54	71.81	19.03
		Average	1.08±0.005	24.52±0.02	71.71±0.10	18.90±0.13
	Forward		1.08	24.55	70.99	18.82
Ph‐H	Reverse	Best	1.09	24.77	73.07	19.73
		Average	1.08±0.003	24.76±0.005	73.06±0.01	19.59±0.14
	Forward		1.09	24.84	72.49	19.63
CF3‐H	Reverse	Best	1.14	25.31	77.39	22.33
		Average	1.13±0.009	25.30±0.005	77.35±0.005	22.15±0.18
	Forward		1.13	25.25	77.47	22.10
CF3‐mF	Reverse	Best	1.16	25.49	79.18	23.41
		Average	1.15±0.007	25.48±0.01	79.17±0.001	23.28±0.13
	Forward		1.15	25.43	78.93	23.08
CF3‐oF	Reverse	Best	1.18	25.51	80.16	24.13
		Average	1.17±0.006	25.50±0.01	80.15±0.001	24.02±0.11
	Forward		1.18	25.50	79.55	23.94
Spiro‐OMeTAD	Reverse	Best	1.16	25.28	78.54	23.03
		Average	1.15±0.008	25.27±0.007	78.53±0.005	22.87±0.16
	Forward		1.15	25.32	78.40	22.83

**Figure 10:**
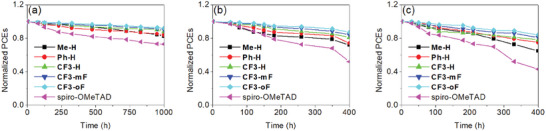
Normalized PCE of unencapsulated devices with different HTMs a) under ambient conditions (20–25 °C); b) under heating conditions (85 °C); and c) continuous 1 sun illumination at 40 °C in N_2_.

The HTM in a PSC with n‐i‐p configuration plays a key role in the device's stability. To identify the effect of the substituent of the current series of HTM on the long‐term stability of PSCs device, the un‐encapsulated **Me‐H**, **Ph‐H**, **CF3‐**series, and spiro‐OMeTAD‐based devices were examined under the atmospheric environment with a relative humidity of 40% in a glove box. As shown in **Figure** **10**a, the PSCs with **Me‐H** and **Ph‐H** maintained 82 and 84% respectively of their initial PCE after 1000 h storage in the dark. In contrast, the spiro‐OMeTAD‐based device retained 73% of its initial value after 1000 h. Compared with the **Ph‐H** counterpart, the **CF3‐H**‐based device preserved 88% of its initial PCE after 1000 h, demonstrating that the PSCs with trifluoromethyl‐substituted **CF3**‐series afford better operation stability. Furthermore, devices containing **CF3‐mF** and **CF3‐oF** as HTM both achieved over 90% of the original efficiency. The improvement in humidity stability could be attributed to the improved hydrophobicity of the perovskite films covered with **CF3**‐series layers. As illustrated in Figure S60 (Supporting Information), the surface of **Me‐H**, **Ph‐H**, and spiro‐OMeTAD films exhibited a hydrophobic characteristic with a water contact angle of 93°, 94° and 73°, respectively. The angles further increase to 95°, 97° and 97° on **CF3‐H**, **CF3‐mF**, and **CF3‐oF** films respectively, indicating that **CF3‐**series molecules further improve the resistance to humidity and should be conducive to the long‐term stability of PSCs devices. Moreover, **CF3‐**series‐based devices also showed better thermal stability in ambient air (Figure 10b). The **CF3‐H**‐, **CF3‐mF**‐, and **CF3‐oF**‐based devices retained 81, 84, and 87% of their original PCEs at 85 °C after 400 h, in contrast to 72, 74, and 52% for the devices with **Me‐H**, **Ph‐H** and spiro‐OMeTAD, respectively. Furthermore, the light‐soaking stability of all devices was evaluated under continuous illumination in a glove box. As shown in Figure 10c, compared to the **Me‐H**‐, **Ph‐H**‐ and spiro‐OMeTAD‐based devices which retained 65, 75, and 43% of initial PCE after 400 h, the devices with **CF3‐**series as HTM were more stable, with over 78% of their initial PCE retained after 400 h. The enhanced light stability of **CF3‐**series‐based devices can be attributed to a more stable amorphous film, in agreement with the DSC analysis (Figure S49b, Supporting Information). These results proved that high‐performance PSCs with excellent device stability were obtained based on the newly designed trifluoromethyl‐substituted HTMs.

## 3 Conclusion

The performance of PSCs can be improved through molecular engineering of the HTM. In this work, a series of 5*H*‑Dithieno[3,2‑b:2′,3′‑d]pyran (DTP)‐based HTMs, named **Me‐H**, **Ph‐H**, **CF3‐H**, **CF3‐mF**, and **CF3‐oF** were designed and synthesized for application in PSC fabrication. We systematically investigate the influence of substituent groups on energy levels, film‐formation properties, hole extraction, and defect passivation effect at the interface of the DTP‐based HTMs with perovskite. The HTM **Ph‐H** with two phenyl rings on DTP moiety was shown to have strengthened intermolecular stacking, thus enhanced charge extraction and transport, improved film formation, and achieved higher PCE compared with HTM **Me‐H**, where two methyl groups were the substituents. As a result, the PCE of **Ph‐H**‐based devices reached 19.73%, which is higher than that of **Me‐H‐**based devices (19.03%). By introducing two trifluoromethyl groups on each of the phenyl rings and additional fluorine atoms on the triphenylamine units (the **CF3** series), further down‐shift of the HOMO energy levels and thus better match with the valence band of the perovskite layer was achieved. Better film‐forming properties, higher hole mobilities, and charge transport properties, as well as better defects‐passivation effects at the interface of the perovskite film, were observed. As a consequence, the PCEs of the PSC devices based on **CF3‐H**, **CF3‐mF**, and **CF3‐oF** were 22.33, 23.41, and 24.13%, respectively. The substitution group on the DTP unit has a great influence on the photovoltaic performance and fluorination of the molecule in general will benefit the overall device performance through different factors. The **CF3‐oF**‐based device achieved the highest PCE, which could be attributed to the contribution of its better energy level alignment, better hole‐extraction efficiency, and excellent film morphology properties. Moreover, we showed that large‐area (1.00 cm^2^) PSCs containing **CF3‐oF** can yield a descent PCE of 22.31%. In addition, the **CF3‐oF**‐based device exhibited better air, thermal, and light soaking stability, as compared with the other DTP‐based HTMs. The low‐cost preparation also makes it a promising candidate for practical application. This work also provides an effective strategy for the rational molecular design of HTMs in PSC fabrication to improve efficiency and stability.

## Conflict of Interest

The authors declare no conflict of interest.

## Supporting information



Supporting Information

Supplemental Table 1

## Data Availability

The data that support the findings of this study are available in the supplementary material of this article.

## References

[1] a) Q. Cheng , H. Chen , F. Yang , Z. Chen , W. Chen , H. Yang , Y. Shen , X. M. Ou , Y. Wu , Y. Li , Y. Li , Angew. Chem., Int. Ed. 2022, 61, e202210613; 10.1002/anie.202210613 36064999

[2] a) A. Kojima , K. Teshima , Y. Shirai , T. Miyasaka , J. Am. Chem. Soc. 2009, 131, 6050; 19366264 10.1021/ja809598r

[3] a) X. Lin , D. Cui , X. Luo , C. Zhang , Q. Han , Y. Wang , L. Han , Energy Environ. Sci. 2020, 13, 3823;

[4] a) Y. Dong , J. Zhang , Y. Yang , L. Qiu , D. Xia , K. Lin , J. Wang , X. Fan , R. Fan , Angew. Chem., Int. Ed. 2019, 58, 17610; 10.1002/anie.201909291 31591794

[5] a) J. Wang , X. Wu , Y. Liu , T. Qin , K. Zhang , N. Li , J. Zhao , R. Ye , Z. Fan , Z. Chi , Z. Zhu , Adv. Energy Mater. 2021, 11, 2100967;

[6] a) J. Yang , J. Huang , C. Zhang , H. Sun , B. Li , Y. Wang , K. Feng , Q. Liao , Q. Bai , L. Niu , H. Wang , X. Guo , Adv. Funct. Mater. 2022, 32, 2206311;

[7] a) J. Zhang , B. Xu , L. Yang , C. Ruan , L. Wang , P. Liu , W. Zhang , N. Vlachopoulos , L. Kloo , G. Boschloo , L. Sun , A. Hagfeldt , E. M. J. Johansson , Adv. Energy Mater. 2018, 8, 1701209;

[8] a) H. D. Pham , T. T. Do , J. Kim , C. Charbonneau , S. Manzhos , K. Feron , W. C. Tsoi , J. R. Durrant , S. M. Jain , P. Sonar , Adv. Energy Mater. 2018, 8, 1703007;

[9] a) L. Tao , B. Wang , H. Wang , C. Chen , X. Ding , Y. Tian , H. Lu , X. Yang , M. Cheng , ACS Appl. Mater. Interfaces 2021, 13, 13470; 33705094 10.1021/acsami.0c21975

[10] a) A. Molina‐Ontoria , I. Zimmermann , I. Garcia‐Benito , P. Gratia , C. Roldan‐Carmona , S. Aghazada , M. Grätzel , M. K. Nazeeruddin , N. Martin , Angew. Chem., Int. Ed. 2016, 128, 6378; 10.1002/anie.201511877 27061436

[11] a) Y.‐D. Lin , K.‐M. Lee , B.‐Y. Ke , K.‐S. Chen , H.‐C. Cheng , W.‐J. Lin , S. H. Chang , C.‐G. Wu , M.‐C. Kuo , H.‐C. Chung , C.‐C. Chou , H.‐Y. Chen , K.‐L. Liau , T. J. Chow , S.‐S. Sun , Energy Technol. 2019, 7, 307;

[12] L. Dou , C.‐C. Chen , K. Yoshimura , K. Ohya , W.‐H Chang , J. Gao , Y. Liu , E. Richard , Y. Yang , Macromolecules 2013, 46, 3384.

[13] a) X. Wang , A. Tang , F. Chen , E. Zhou , Polym. Chem. 2017, 8, 5396;

[14] M. Caicedo‐Reina , M. Pérez‐Escribano , J. Urieta‐Mora , I. García‐Benito , J. Calbo , A. Ortiz , B. Insuasty , A. Molina‐Ontoria , E. Ortí , N. Martín , J. Mater. Chem. C 2023, 11, 8223.

[15] Y. He , N. Li , T. Heumüller , J. Wortmann , B. Hanisch , A. Aubele , S. Lucas , G. Feng , X. Jiang , W. Li , P. B¨auerle , C. J. Brabec , Joule 2022, 6, 1160.

[16] J. Johansson , L. Kloo , A. Hagfeldt , A.‐Y. Jen , L. Sun , Chem 2017, 2, 676.

[17] a) H. Wang , F. Zhang , Z. Li , J. Zhang , J. Lian , J. Song , J. Qu , W.‐Y. Wong , Chem. Eng. J. 2020, 395, 125062,

[18] X. Ji , T. Zhou , Q. Fu , W. Wang , Z. Wu , M. Zhang , X. Guo , D. Liu , H. Y. Woo , Y. Liu , Adv. Energy Mater. 2023, 2203756.

[19] M. Jeong , I. W. Choi , E. M. Go , Y. Cho , M. Kim , B. Lee , S. Jeong , Y. Jo , H. W. Choi , J. Lee , J.‐H. Bae , S. K. Kwak , D. S. Kim , C. Yang , Science 2020, 369, 1615. 32973026 10.1126/science.abb7167

