# Supplementary material for: Judicious Molecular Design of 5H‑Dithieno[3,2‑b:2′,3′‑d]Pyran‐based Hole‐Transporting Materials for Highly Efficient and Stable Perovskite Solar Cells
Source: Adv Sci (Weinh). 2024 Nov 28;12(3):2410666. doi: 10.1002/advs.202410666 (PMC11744647; doi:10.1002/advs.202410666)
Supplement: Supplementary file 1 — Supporting Information [file ADVS-12-2410666-s002.docx]

Supporting Information

Judicious Molecular Design of 5*H*‑Dithieno[3,2‑b:2′,3′‑d]pyran-based Hole-Transporting Materials for Highly Efficient and Stable Perovskite Solar Cells

Kun-Mu Lee,*^a,b,c,d^ Chia-Hui Lin,^e^ Chia-Chi Chang,^e^ Ting-Yu Yang,^e^ Wei-Hao Chiu,^b^ Wei-Chen Chu, ^a^ Ya-Ho Chang,^b^ Sie-Rong Li,^f^ Shih- I Lu,*^e^ Hsiao-Chi Hsieh,*^g^ Kang-Ling Liau,^h^ Chia Hui Hu,^e^ Chih-Hung Chen,^i^ Yun-Shuo Liu,^e^ Wei-Chun Chou,^e^ Mandy M. Lee,^f^ Shih-Sheng Sun,*^f^ Yu-Tai Tao^f^ and Yan-Duo Lin*^e^

^a^ Department of Chemical and Materials Engineering, Chang Gung University, Taoyuan 33302, Taiwan

^b^ Center for Sustainability and Energy Technologies, Chang Gung University, Taoyuan 33302, Taiwan

^c^ Division of Neonatology, Department of Pediatrics, Chang Gung Memorial Hospital, Linkou, Taoyuan 33305, Taiwan

^d^ College of Environment and Resources, Ming Chi University of Technology, New Taipei City 24301, Taiwan

^e^ Department of Chemistry, Soochow University, Taipei 11102, Taiwan

^f^ Institute of Chemistry, Academia Sinica, Taipei 115024, Taiwan

^g^ Department of Chemical and Materials Engineering, Tamkang University, Tamsui District, New Taipei City, 25137, Taiwan

^h^ Department of Chemistry, National Central University, Taoyuan 32001, Taiwan

^i^ Department of Materials Science and Engineering, National Taiwan University of Science and Technology, Taipei 106335, Taiwan

† Supplementary information (SI) available. Other electronic format see DOI: XXX

Email: ydlin@scu.edu.tw

**Materials and Reagents**

All solvents and chemicals were purchased from Aldrich, with the purity more than 98%. The thin-layer chromatography (TLC) was conducted with Merck KGaA precoated TLC Silica gel 60F254 on aluminum sheets. Flash column chromatography was performed on glass columns packed with silica gel (Silicycle UltraPure SilicaFlash P60, 40−63 mm (230−400 mesh)). Unless otherwise specified, all reactions and manipulations were carried out under nitrogen atmosphere. Solvents of reagent grade were used for syntheses and those of spectroscopy grade for spectra measurements. Solvents were dried by standard procedures.

**Characterization**

The ^1^H and ^13^C NMR spectra were recorded on a Bruker 400 or 500 MHz spectrometer. Fast atom bombardment (FAB) mass spectra were recorded on a Jeol JMS 700 double-focusing spectrometer. The UV spectra were measured on a Jasco V-530 double beam spectrophotometer. Fluorescence spectra were recorded on a Hitachi F-4500 fluorescence spectrophotometer. Cyclic voltammetry experiments were performed with a CHI-621A electrochemical analyzer. All measurements were carried out at room temperature with a conventional three-electrode configuration that consisted of a platinum working electrode, an auxiliary electrode, and a non-aqueous Ag/AgNO_3_ reference electrode. The SEM images were obtained by using a field-emission scanning electron microscope (JEOL-7401). A Nano-Scope NS3A system (Digital Instrument) was used to obtain the AFM images of the surface morphologies and thicknesses of various thin films. X-ray photoelectron spectroscopy (XPS) was collected with a VG ESCA Scientific Theta Probe system with a monochromatic Al Kα source (1486.6 eV) operated under ultra-high vacuum (< 2.5 × 10^−10^ Torr) and analyzed by XPSPEAKS41 software. In addition, charge neutralization was used during all measurements. All binding energy were calibrated by shifting the C 1s peak to 284.8 eV.

**Solar cell fabrication.**

The compact TiO_2_ layer (~30 nm in thickness) was deposited by spin-coating a titanium isopropyl alcohol solution onto a F:SnO_2_ (FTO) substrate. A 150 nm-thick mesoporous TiO_2_ film (particle size: ~20 nm, crystalline phase: anatase) was screen-printed onto the compact-TiO­_2_/FTO substrate using home-made pastes and heated at 500 °C for 30 min. After sintering the TiO_2_ layer, the electrode was cooled to room temperature and immersed in 0.04 M aqueous TiCl_4_ at 70 °C for 30 min. The substrate was then rinsed by deionized water and then annealed at 500 °C for 30 min again. After cooling to room temperature the substrate was transferred to a nitrogen-filled glove box. Then, the perovskite (Cs_0.05_MA_0.2_FA_0.75_Pb(Br_0.05_I_0.95_)_3_) layer was prepared using a single-step method. The mixture of 576.3 mg of PbI_2_ (99.9985%, Alfa Aesar, Harverhill, MA, USA), 161.2 mg of formamidinium iodide (FAI, 99.99%, FMPV^®^, FrontMaterials Co. Ltd., Taipei, Taiwan), 14 mg of methylammonium bromide (MABr, ECHO chemical, Miaoli, Taiwan), 16.2 mg of Cesium iodide (CsI, Aldrich, Burlington, VT, USA) and 19.8 mg of methylammonium iodide (CH_3_NH_3_I, MAI, > 98%, STAREK^®^, Starek Scientific Co. Ltd., Taipei, Taiwan) dissolved in 0.8 mL dimethylformamide (DMF, Tedia, OH, USA) and 0.2 mL Dimethyl sulfoxide (DMSO, 99%, Echo Chemical Co., Ltd., Miaoli, Taiwan) was stirred at 60 ^°^C for 8 h and was coated onto the TiO­_2_ substrate by a two-step spin-coating process at 1000 and 5000 rpm for 10 and 20 sec, respectively. At the second spin-coating step, the substrate/films were treated with 75 μL toluene by drop-casting. The substrate was dried at 100 °C on a hot plate for 10 min. The **Me-H**, **Ph-H**, and **CF3-**series studied in this work were dissolved in chlorobenzene (40 mg/mL) and spin-coated on the substrate at 2000 rpm for 30 s. The solution of spiro-OMeTAD was prepared in chlorobenzene (40 mg/mL) and mixed with 17.5 μL solution of lithium bis-trifluoromethanesulfonimide (Li-TSFI, 520 mg in 1 mL acetonitrile) and 28.5 μL 4-*tert*-butylpyridine. The solution was spin-coated on substrate at 2000 rpm for 30 s. Finally, the Ag counter electrode (~100 nm) was deposited by thermal evaporation. The active area of the electrode was fixed at 0.16 cm^2^. *J*-*V* curves were recorded with a Keithley 2400 source meter under simulated AM 1.5G sunlight, calibrated to 100 mW/cm^2^. The reported device characteristics were estimated from the measured *J*-*V* curves.

**Solar cell performance measurement**

Solar cell efficiencies were evaluated under simulated one sun irradiation from a Xe arc lamp with an AM 1.5 global filter. Irradiance was characterized using a calibrated spectrometer and illumination intensity was set using an NREL-certified silicon diode with an integrated KG1 optical filter. Spectral mismatch factors were calculated for each device in this report to be less than 5%. IPCE spectra were measured in air using a commercial IPCE set-up (Enlitech, QE-R).

**Mobility measurements**

In order to assess the potential of **Me-H**, **Ph-H**, **CF3-H**, **CF3-mF**, **CF3-oF** and spiro-OMeTAD as hole-transport materials, hole-only devices were fabricated with the device structure of ITO/PEDOT:PSS/HTM/Al. Hole mobilities were calculated by the space-charge-limited current (SCLC) method using the Mott–Gurney law, by fitting experimental data to equation (1) in the voltage range where the obtained slope in the double log plot is equal to 2.

(1)

In equation (1), *J* is the current density, ε_0_ is the permittivity of free space (8.85 x10^-12^ F m^-1^), ε_r_ is the relative permittivity of the material (approaching 3 for organic semiconductors), μ_h_ is the hole mobility, *V* is the applied voltage and *d* is the thickness of the active layer.

The hole-only devices were fabricated by spin-coating PEDOT:PSS (Clevios P, VP Al4083) onto pre-cleaned, patterned indium tin oxide (ITO) substrates (15 Ω/square) (Kintec). A film of the HTM was spin-coated on top from chloroform solution with a concentration of 40 mg mL^-1^. The film thickness was varied by using different spin-coating speeds. As a counter electrode, Al was deposited on top by vacuum evaporation. The current density–voltage curves of the devices were recorded with a Keithley 2400 Source meter.

**GIWAXS** **measurements**

Grazing-Incidence Wide-Angle X-ray Scattering (GIWAXS) was measured at the beamline 01C, 17A, and 13A of the Taiwan Light Source in the National Synchrotron Radiation Research Center (NSRRC) at a distance of ∼240 nm from the sample to the detector. The incident angle was 0.2°. The crystalline correlation length (Lc) was calculated from the Scherrer equation^[1]^

Lc = 0.9 × 2π/fwhm (2)

where fwhm is the full width at half-maximum of a peak. Near-edge X-ray absorption fine structure (NEXAFS) spectroscopy was performed in a wide-range beamline (BL24A) of the Taiwan Light Source of NSRRC based on the total electron yield detection mode.

**Computation method**

Ground-state geometries and the corresponding vertical excitation energies of the **Me-H**, **Ph-H**, **CF3-H**, **CF3-mF**, **CF3-oF** molecules were studied computationally by performing B3LYP and TD-B3LYP calculations, respectively, with the 6-31G(d,p) basis set via Gaussian 16 software package.^[2]^ To further study the interaction between perovskite and **Me-H**, **Ph-H**, and **CF3-**series, we conducted geometry optimization within the context of periodic boundary conditions (PBC) in which one molecule of **Me-H**, **Ph-H**, **CF3-H**, **CF3-mF**, **CF3-oF** was placed over the perovskite surface of 4x4x2 cell. The density functional based tight binding method using the GFN1-xTB ^[3]^ parametrization of the extended tight-binding (xTB) model Hamiltonian was employed. We used the DFTB engine ^[4]^ implemented in the Amsterdam Modeling Suite 2022 ^[5]^ to perform the calculations.

**Scheme S1.** Synthetic procedures for **Me-H**, **Ph-H**, and **CF3**-series HTMs.

*Synthesis of* ***2a****:* A heterogeneous mixture of anhydrous THF (12 mL), **1** (0.5 g, 2.4 mmol), and methylmagnesium bromide, 1 M to 1.5 M solution in THF (6 mL, 6 mmol) was stirred under argon at -20 °C for 0.5 h. The mixture was extracted with ethyl acetate. The organic layer was separated and dried over anhydrous MgSO_4_. Evaporation of the solvent gave a crude product, which was purified by silica gel column chromatography using ethyl acetate/hexane (1/10) as eluent to afford the desired product as a white solid in 93% yield. mp 108-109 °C; ^1^H NMR (500 MHz, CDCl_3_): δ 7.23 (d, J = 5.0 Hz, 1H), 7.16 (d, J = 5.5 Hz, 1H), 7.06 (d, J = 5.5 Hz, 1H), 6.71 (d, J = 5.0 Hz, 1H), 1.64 (s, 6H) ppm; ^13^C NMR (125 MHz, CDCl_3_): δ 151.12, 144.04, 130.71, 127.56, 125.42, 124.91, 121.67, 110.41, 72.10, 30.45 ppm.; HRMS (FAB) m/z [M^+^] calcd for C_11_H_12_O_2_S_2_: 240.0279; found: 240.0279.

*Synthesis of* ***2b****:* A heterogeneous mixture of anhydrous THF (12 mL), **1** (0.5 g, 2.4 mmol), and 1.0 M phenylmagnesium bromide solution in THF (6 mL, 6 mmol) under argon was stirred at -20 °C for 0.5 h. The mixture was extracted with ethyl acetate. The organic layer was separated and dried over anhydrous MgSO_4_. Evaporation of the solvent gave a crude product, which was purified by silica gel column chromatography using ethyl acetate/hexane (1/12) as eluent to afford the desired product as a white solid in 94% yield. mp 118-119 °C; ^1^H NMR (500 MHz, CDCl_3_): δ 7.60 (s, 1H), 7.30 (d, J = 7.5 Hz, 4H), 7.24 (t, J = 7.0 Hz, 4H), 7.20 (d, J = 7.5 Hz, 2H), 7.18 (d, J = 5.5 Hz, 1H), 6.95 (d, J = 5.5 Hz, 1H), 6.48 (d, J = 5.5 Hz, 1H), 6.42 (d, J = 5.5 Hz, 1H) ppm; ^13^C NMR (125 MHz, CDCl_3_): δ 151.17, 145.45, 144.92, 131.88, 131.29, 128.17, 127.65, 127.00, 125.38, 125.10, 120.63, 109.67, 80.69 ppm.; HRMS (ESIneg) m/z [M-H^-^] calcd for C_21_H_15_O_2_S_2_: 363.05189; found:363.0515.

*Synthesis of* ***2c****:* A heterogeneous mixture of anhydrous THF (12 mL), **1** (0.5 g, 2.4 mmol), and 0.5 M 3,5-bis(trifluoromethyl)phenylmagnesium bromide solution in THF (12 mL, 6 mmol) was stirred under argon at -20 °C for 0.5 h. The mixture was extracted with ethyl acetate. The organic layer was separated and dried over anhydrous MgSO_4_. Evaporation of the solvent gave a crude product, which was purified by silica gel column chromatography using ethyl acetate/hexane (1/15) as eluent to afford the desired product as yellow liquid in 98% yield. ^1^H NMR (500 MHz, CDCl_3_): δ 7.93 (s, 4H), 7.68 (s, 2H), 7.35 (d, J = 5.5 Hz, 1H), 6.93 (d, J = 5.5 Hz, 1H), 6.61 (s, 1H), 6.49 (d, J = 5.5 Hz, 1H), 6.26 (d, J = 5.5 Hz, 1H), 5.15 (s, 1H) ppm; ^13^C NMR (125 MHz, CDCl_3_): δ 149.40, 147.59, 142.84, 132.06, 131.83, 131.56, 130.05, 126.99, 126.73, 126.58, 124.41, 121.80, 119.39, 110.02 ppm.; HRMS (FAB) m/z [M+H^+^] calcd for C_25_H_12_F_12_O_2_S_2_: 636.0087; found: 637.0163.

*Synthesis of* ***3a****:* A heterogeneous mixture of anhydrous toluene (15 mL), **2a** (0.5 g, 2.08 mmol), and 4-methylbenzene-1-sulfonic acid (0.035 g, 0.208 mmol) under argon was stirred under reflux for 12 h. The mixture was cooled and extracted with ethyl acetate. The organic layer was separated and dried over anhydrous MgSO_4_. Evaporation of the solvent gave a crude product, which was purified by silica gel column chromatography using ethyl acetate/hexane (1/60) as eluent to afford the desired product as yellow liquid in 94% yield. ^1^H NMR (500 MHz, CDCl_3_): δ 7.04 (d, J = 5.0 Hz, 1H), 6.98 (d, J = 5.0 Hz, 1H), 6.79 (d, J = 5.0 Hz, 1H), 6.69 (d, J = 5.0 Hz, 1H), 1.63(s, 6H) ppm; ^13^C NMR (125 MHz, CDCl_3_): δ 150.73, 135.23, 128.24, 123.73, 121.62, 121.10, 119.02, 111.44, 80.85, 27.98 ppm.; HRMS (FAB) m/z [M^+^] calcd for C_11_H_10_OS_2_: 222.0173; found: 222.0170.

*Synthesis of* ***3b****:* A heterogeneous mixture of anhydrous toluene (15 mL), **2b** (0.5 g, 1.37 mmol), and 4-methylbenzene-1-sulfonic acid (0.024 g, 0.137 mmol) under argon was stirred under reflux for 12 h. The mixture was cooled and extracted with ethyl acetate. The organic layer was separated and dried over anhydrous MgSO_4_. Evaporation of the solvent gave a crude product, which was purified by silica gel column chromatography using ethyl acetate/hexane (1/60) as eluent to afford the desired product as a white solid in 96% yield. mp 125-126 °C; ^1^H NMR (500 MHz, DMSO-d6): δ 7.39 (d, J = 5.0 Hz, 1H), 7.36-7.31 (m, 7H), 7.21-7.19 (m, 4H), 6.94 (d, J = 5.0 Hz, 1H), 6.50 (d, J = 5.0 Hz, 1H) ppm; ^13^C NMR (125 MHz, DMSO-d6): δ 150.25, 143.19, 132.41, 128.57, 128.05, 128.02, 127.42, 127.14, 123.03, 122.21, 119.06, 111.63, 87.38 ppm.; HRMS (MALDI) m/z [M+H^+^] calcd for C_21_H_14_OS_2_: 347.0564; found: 347.0569.

*Synthesis of* ***3c****:* A heterogeneous mixture of anhydrous toluene (15 mL), **2c** (0.5 g, 0.786 mmol), and 4-methylbenzene-1-sulfonic acid (0.014 g, 0.079 mmol) under argon was stirred under reflux for 12 h. The mixture was extracted with ethyl acetate. The organic layer was separated and dried over anhydrous MgSO_4_. Evaporation of the solvent gave a crude product, which was purified by silica gel column chromatography using ethyl acetate/hexane (1/60) as eluent to afford the desired product as a white solid in 97% yield. mp 108-109 °C; ^1^H NMR (500 MHz, CDCl_3_): δ 7.86 (s, 2H), 7.68 (s, 4H), 7.17 (d, J = 5.5 Hz, 1H), 7.06 (d, J = 5.5 Hz, 1H), 6.79 (d, J = 5.5 Hz, 1H), 6.39 (d, J = 5.0 Hz, 1H) ppm; ^13^C NMR (125 MHz, CDCl_3_): δ 149.41, 145.36, 132.27, 132.00, 129.26, 127.99, 126.02, 123.43, 123.12, 122.10, 119.93, 118.59, 113.39, 86.29 ppm.; HRMS (MALDI) m/z [M+H^+^] calcd for C_25_H_10_F_12_OS_2_: 619.0059; found: 619.0051.

*Synthesis of* ***4a***: A heterogeneous mixture of CH_2_Cl_2_ (20 mL), **3a** (0.5 g, 2.25 mmol), and 1-bromo-2,5-pyrolidinedione (0.84 g, 4.72 mmol) under room temperature was stirred for 1 h. The mixture was extracted with CH_2_Cl_2_. The organic layer was separated and dried over anhydrous MgSO_4_. Evaporation of the solvent gave a crude product, which was purified by silica gel column chromatography using hexane as eluent to afford the desired product as a white solid in 90% yield. mp 75-76 °C; ^1^H NMR (500 MHz, CDCl_3_): δ 6.73 (s, 1H), 6.66 (s, 1H), 1.57 (s, 6H) ppm; ^13^C NMR (125 MHz, CDCl_3_): δ 149.77, 134.87, 128.54, 126.63, 122.24, 112.05, 109.84, 108.94, 80.56, 27.73 ppm.; HRMS (FAB) *m*/*z* [M^+^] calcd for C_11_H_8_Br_2_OS_2_: 377.8383; found: 377.8380.

*Synthesis of* ***4b***: A heterogeneous mixture of CH_2_Cl_2_ (20 mL), **3b** (0.5 g, 1.44 mmol), and 1-bromo-2,5-pyrolidinedione (0.54 g, 3.03 mmol) under room temperature was stirred for 1 h. The mixture was extracted with CH_2_Cl_2_. The organic layer was separated and dried over anhydrous MgSO_4_. Evaporation of the solvent gave a crude product, which was purified by silica gel column chromatography using hexane as eluent to afford the desired product as a white solid in 90% yield. mp 201-202 °C; ^1^H NMR (500 MHz, CDCl_3_): δ 7.30 (s, 6H), 7.23 (s, 4H), 6.75 (s, 1H), 6.38 (s, 1H) ppm; ^13^C NMR (125 MHz, CDCl_3_): δ 149.87, 142.98, 132.59, 130.11, 130.05, 128.51, 128.24, 128.12, 122.22, 113.45, 110.63, 108.40, 87.75 ppm.; HRMS (MALDI) *m*/*z* [M+H^+^] calcd for C_21_H_12_Br_2_OS_2_: 502.8774; found: 502.8769.

*Synthesis of* ***4c***: A heterogeneous mixture of CH_2_Cl_2_ (20 mL), **3c** (0.5 g, 0.808 mmol), and 1-bromo-2,5-pyrolidinedione (0.3 g, 1.7 mmol) under room temperature was stirred for 1 h. The mixture was extracted with CH_2_Cl_2_. The organic layer was separated and dried over anhydrous MgSO_4_. Evaporation of the solvent gave a crude product, which was purified by silica gel column chromatography using hexane as eluent to afford the desired product as a white solid in 94% yield. mp 222-223 °C; ^1^H NMR (500 MHz, CDCl_3_): δ 7.90 (s, 2H), 7.64 (s, 4H), 6.78 (s, 1H), 6.28 (s, 1H) ppm; ^13^C NMR (125 MHz, CDCl_3_): δ 148.39, 144.51, 132.55, 132.28, 128.40, 127.88, 123.64, 123.61, 121.99, 121.68, 113.92, 112.61, 110.90, 85.81 ppm.; HRMS (MALDI) *m*/*z* [M^+^] calcd for C_25_H_8_Br_2_F_12_OS_2_: 773.8191; found: 773.8197.

*Synthesis of* ***Me-H***: A heterogeneous mixture of 2 M K_2_CO_3_ (6.9 mL), THF (13.15 mL), **4a** (0.5 g, 1.315 mmol), **5** (1.418 g, 3.288 mmol), and Pd(PPh_3_)_4_ (0.105 g, 8 mol %) under argon was heated at 80 °C for 12 h. The mixture was extracted with ethyl acetate. The organic layer was separated and dried over anhydrous MgSO_4_. Evaporation of the solvent gave a crude product, which was purified by silica gel column chromatography using ethyl acetate/hexane (1/10) as eluent to afford the desired product as a orange red solid in 81% yield. mp 129-130 °C; ^1^H NMR (500 MHz, CDCl_3_): δ 7.32 (d, J = 8.5, 4H), 7.05-7.03 (m, 8H), 6.89-6.86 (m, 5H), 6.83 (t, J = 8.5, 9H) , 3.78 (s, 12H), 1.65 (s, 6H) ppm; ^13^C NMR (125 MHz, CDCl_3_): δ 156.25, 156.16, 151.02, 148.54, 148.20, 140.80, 140.74, 139.73, 135.90, 126.93, 126.79, 126.44, 126.38, 126.05, 125.87, 120.86, 120.52, 118.34, 114.95, 114.93, 113.41, 110.05, 80.54, 55.70, 28.01 ppm.; HRMS (MALDI) *m*/*z* [M^+^] calcd for C_51_H_44_N_2_O_5_S_2_: 828.2692; found: 828.2693.

*Synthesis of* ***Ph-H***: A heterogeneous mixture of 2 M K_2_CO_3_ (5.2 mL), THF (9.9 mL), **4b** (0.5 g, 0.992 mmol), **5** (1.07 g, 2.48 mmol), and Pd(PPh_3_)_4_ (0.092 g, 8 mol %) under argon was heated at 80 °C for 12 h. The mixture was extracted with ethyl acetate. The organic layer was separated and dried over anhydrous MgSO_4_. Evaporation of the solvent gave a crude product, which was purified by silica gel column chromatography using ethyl acetate/hexane (1/10) as eluent to afford the desired product as a orange red solid in 85% yield. mp 162-163 °C; ^1^H NMR (500 MHz, CDCl_3_): δ 7.32-7.25 (m, 14H), 7.03 (t, J = 7.0, 8H), 6.86-6.79 (m, 13H), 6.52 (s, 9H) , 3.77 (s, 12H) ppm; ^13^C NMR (125 MHz, CDCl_3_): δ 156.24, 156.14, 151.18, 148.59, 148.24, 143.84, 140.80, 140.70, 140.35, 139.76, 133.33, 128.30, 128.13, 128.06, 126.90, 126.70, 126.40, 126.22, 126.11, 125.90, 122.00, 120.88, 120.48, 114.93, 114.90, 113.41, 111.29, 87.85, 55.69 ppm.; HRMS (MALDI) *m*/*z* [M^+^] calcd for C_61_H_48_N_2_O_5_S2: 952.3005; found: 952.3008.

*Synthesis of* ***CF3-H***: A heterogeneous mixture of 2 M K_2_CO_3_ (3.4 mL), THF (6.45 mL), **4c** (0.5 g, 0.644 mmol), **5** (0.695 g, 1.61 mmol), and Pd(PPh_3_)_4_ (0.06 g, 8 mol %) under argon was heated at 80 °C for 12 h. The mixture was extracted with ethyl acetate. The organic layer was separated and dried over anhydrous MgSO_4_. Evaporation of the solvent gave a crude product, which was purified by silica gel column chromatography using ethyl acetate/hexane (1/15) as eluent to afford the desired product as a orange solid in 91% yield. mp 191-192 °C; ^1^H NMR (500 MHz, CDCl_3_): δ 7.88 (s, 2H), 7.75 (s, 4H), 7.28 (d, J = 8.5, 2H), 7.25 (d, J = 8.5, 2H), 7.04 (d, J = 7.5, 8H), 6.86-6.80 (m, 13H), 6.38 (s, 1H), 3.78 (s, 12H) ppm; ^13^C NMR (125 MHz, CDCl_3_): δ 156.46, 156.41, 149.58, 149.16, 148.98, 145.45, 142.22, 140.51, 132.50, 132.24, 131.97, 131.70, 129.27, 128.90, 128.10, 127.12, 127.01, 126.34, 126.09, 125.34, 125.15, 124.30, 123.16, 122.13, 120.40, 120.14, 115.00, 114.87, 112.58, 111.52, 85.94, 55.70 ppm.; HRMS (MALDI) *m*/*z* [M^+^] calcd for C_65_H_44_F_12_N_2_O_5_S_2_: 1224.2499; found: 1224.2497.

*Synthesis of* ***CF3-oF***: A heterogeneous mixture of 2 M K_2_CO_3_ (3.4 mL), THF (6.45 mL), **4c** (0.5 g, 0.644 mmol), **6** (0.724 g, 1.61 mmol), and Pd(PPh_3_)_4_ (0.06 g, 8 mol %) under argon was heated at 80 °C for 12 h. The mixture was extracted with ethyl acetate. The organic layer was separated and dried over anhydrous MgSO_4_. Evaporation of the solvent gave a crude product, which was purified by silica gel column chromatography using ethyl acetate/hexane (1/20) as eluent to afford the desired product as a dark orange solid in 86% yield. mp 138-139 °C; ^1^H NMR (500 MHz, CDCl_3_): δ 7.88 (s, 2H), 7.74 (s, 4H), 7.32-7.27 (m, 4H), 7.03-7.01 (m, 4H), 6.90-6.88 (m, 5H), 6.85-6.77 (m, 10H), 6.39(s, 1H), 3.84 (d, J = 2.0, 6H), 3.78 (d, J = 2.0, 6H) ppm; ^13^C NMR (125 MHz, CDCl_3_): δ 156.61, 153.68, 151.75, 149.46, 148.31, 148.14, 143.88, 141.83, 140.90, 139.85, 132.34, 132.07, 131.80, 131.54, 129.22, 129.19, 128.96, 128.88, 128.57, 127.81, 127.26, 126.90, 126.65, 125.63, 125.36, 124.09, 121.89, 121.63, 120.55, 120.33, 119.75, 119.52, 119.39, 119.24, 115.60, 114.83, 114.32, 113.55, 113.37, 112.01, 111.59, 58.38, 57.24, 56.05, 54.95, 53.80 ppm.; HRMS (MALDI) *m*/*z* [M^+^] calcd for C_65_H_42_F_14_N_2_O_5_S_2_: 1260.2311; found: 1260.2326.

*Synthesis of* ***CF3-mF***: A heterogeneous mixture of 2 M K_2_CO_3_ (3.4 mL), THF (6.45 mL), **4c** (0.5 g, 0.644 mmol), **7** (0.724 g, 1.61 mmol), and Pd(PPh_3_)_4_ (0.06 g, 8 mol %) under argon was heated at 80 °C for 12 h. The mixture was extracted with ethyl acetate. The organic layer was separated and dried over anhydrous MgSO_4_. Evaporation of the solvent gave a crude product, which was purified by silica gel column chromatography using ethyl acetate/hexane (1/20) as eluent to afford the desired product as a dark orange solid in 82% yield. mp 138-139 °C; ^1^H NMR (500 MHz, CDCl_3_): δ 7.88 (s, 2H), 7.75 (s, 4H), 7.30-7.25 (m, 4H), 7.13-7.07 (m, 6H), 6.86-6.82 (m, 5H), 6.76-6.73 (m, 4H), 6.69-6.66 (m, 4H), 6.37 (s, 1H), 3.78 (s, 12H) ppm; ^13^C NMR (125 MHz, CDCl_3_): δ 160.38, 160.35, 158.84, 158.81, 158.76, 158.73, 158.39, 158.36, 156.71, 156.66, 149.53, 148.75, 148.56, 145.45, 142.24, 142.19, 139.49, 139.44, 132.48, 132.22, 131.95, 131.68, 130.55, 129.23, 128.86, 128.07, 126.77, 126.14, 125.14, 124.94, 124.28, 123.13, 122.11, 119.76, 118.27, 118.05, 114.95, 112.55, 111.48, 110.96, 110.64, 103.38, 103.19, 85.92, 55.93, 55.67 ppm.; HRMS (MALDI) *m*/*z* [M^+^] calcd for C_65_H_42_F_14_N_2_O_5_S_2_: 1260.2311; found: 1260.2285.


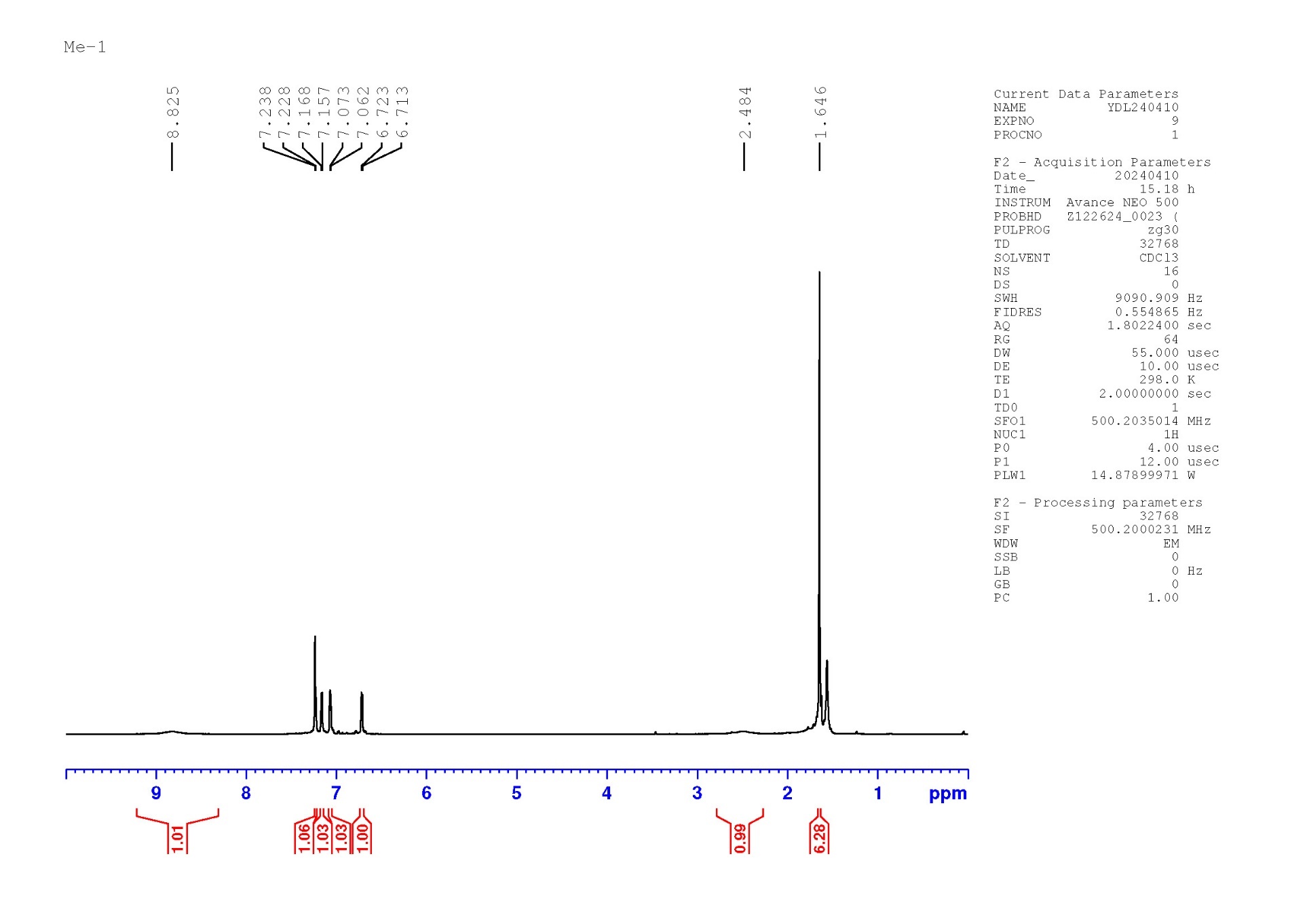


**Figure S1.** ^1^H NMR spectrum of compound **2a**.


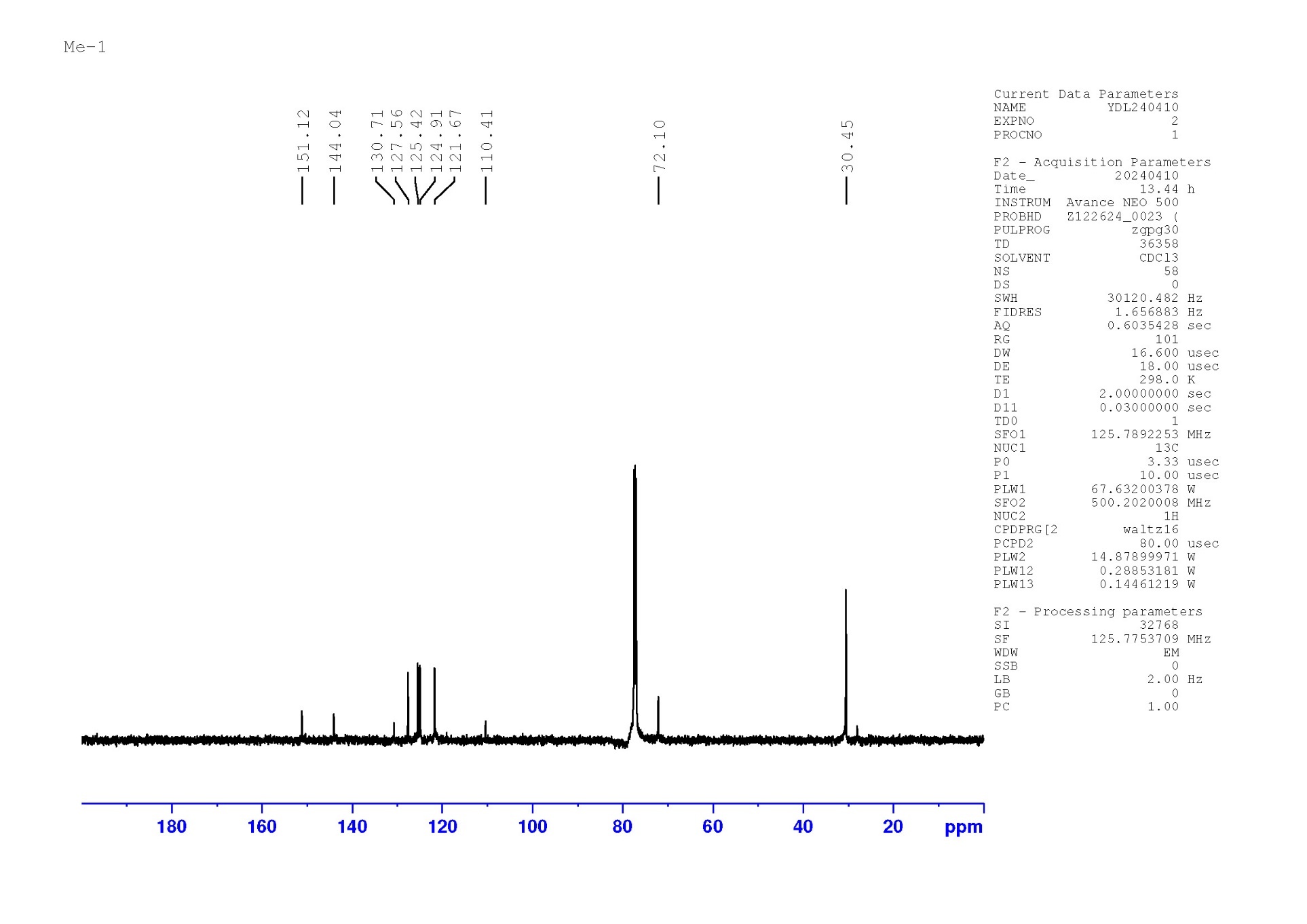


**Figure S2.** ^13^C NMR spectrum of compound **2a**.


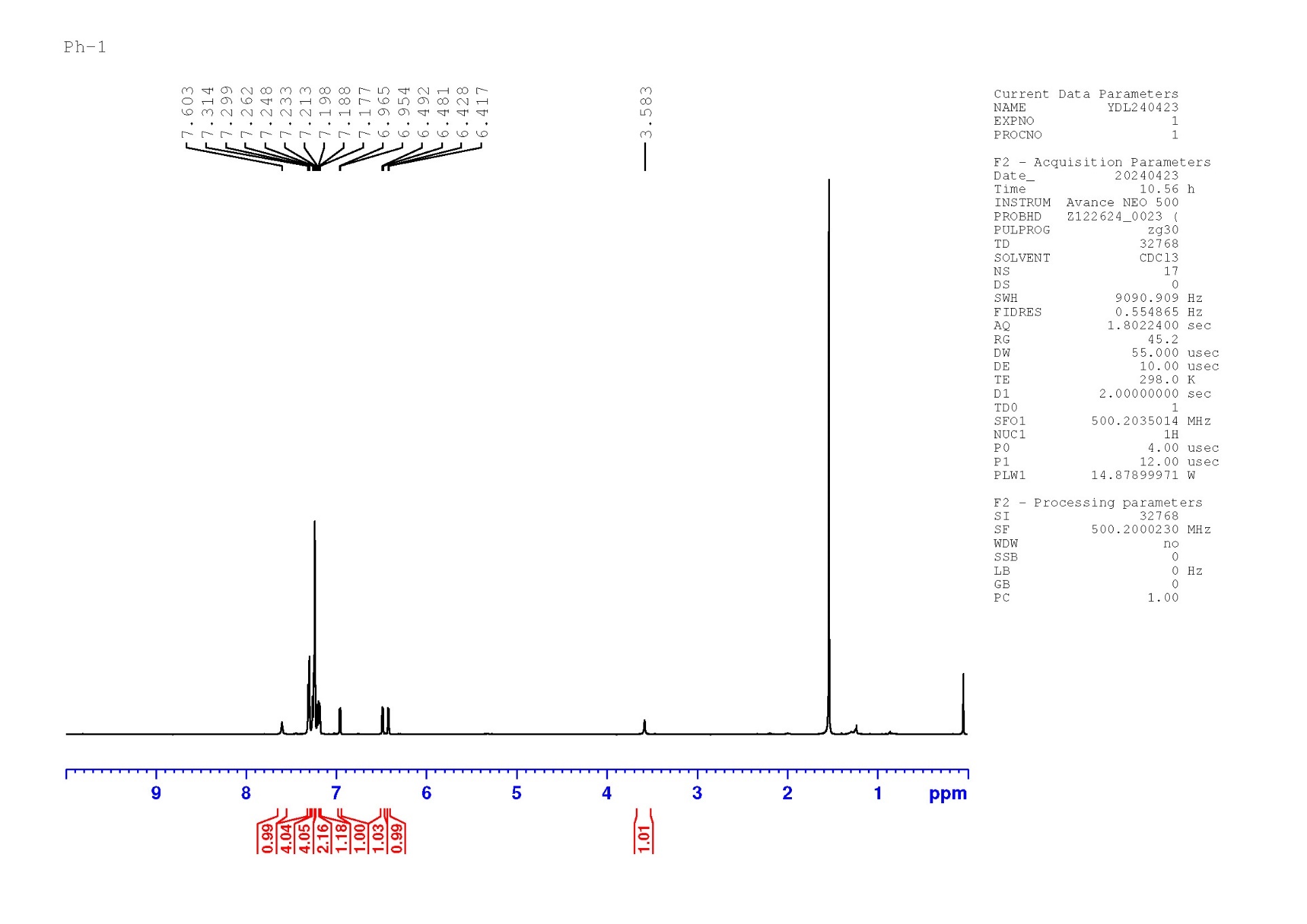


**Figure S3.** ^1^H NMR spectrum of compound **2b**.


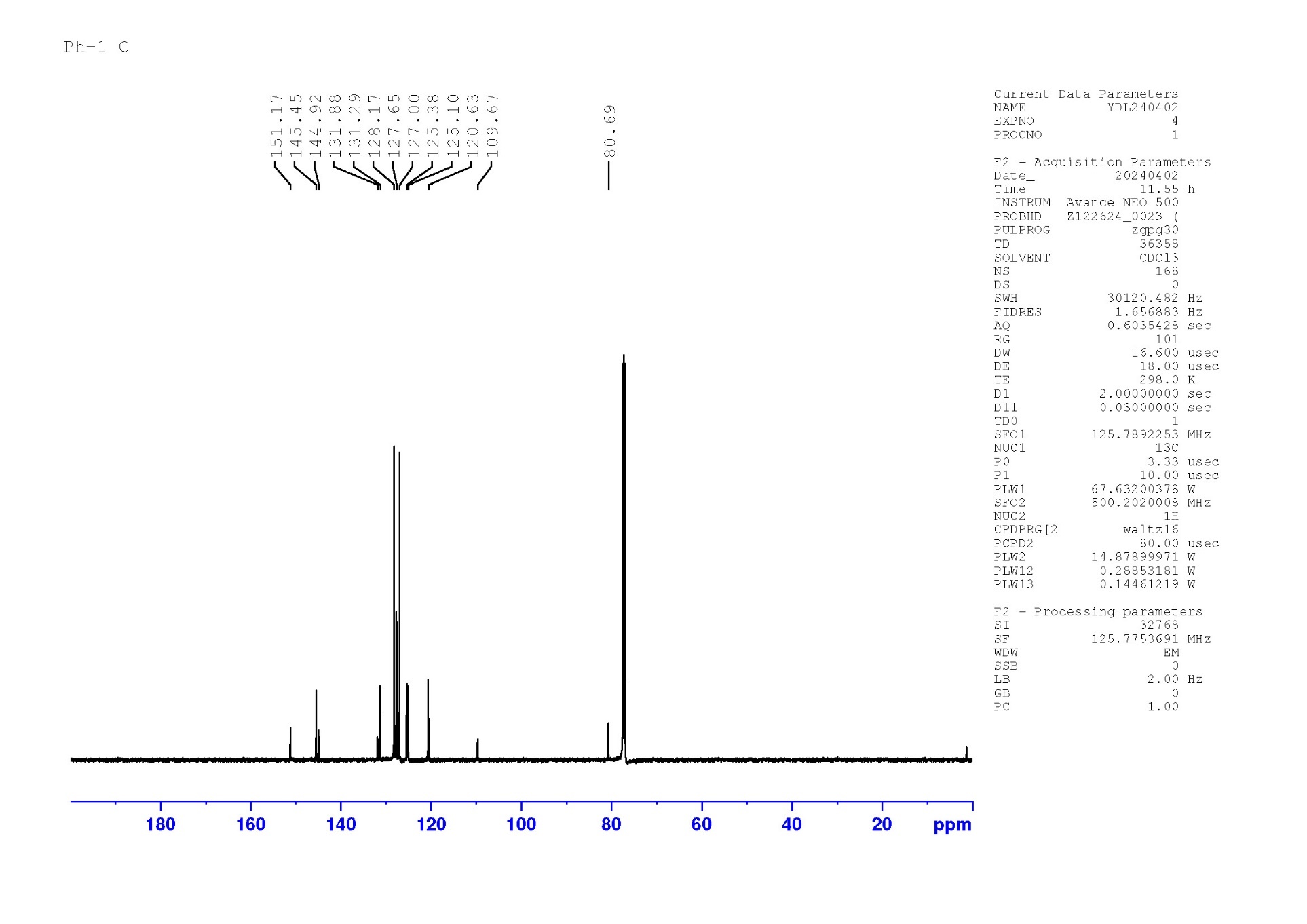


**Figure S4.** ^13^C NMR spectrum of compound **2b**.


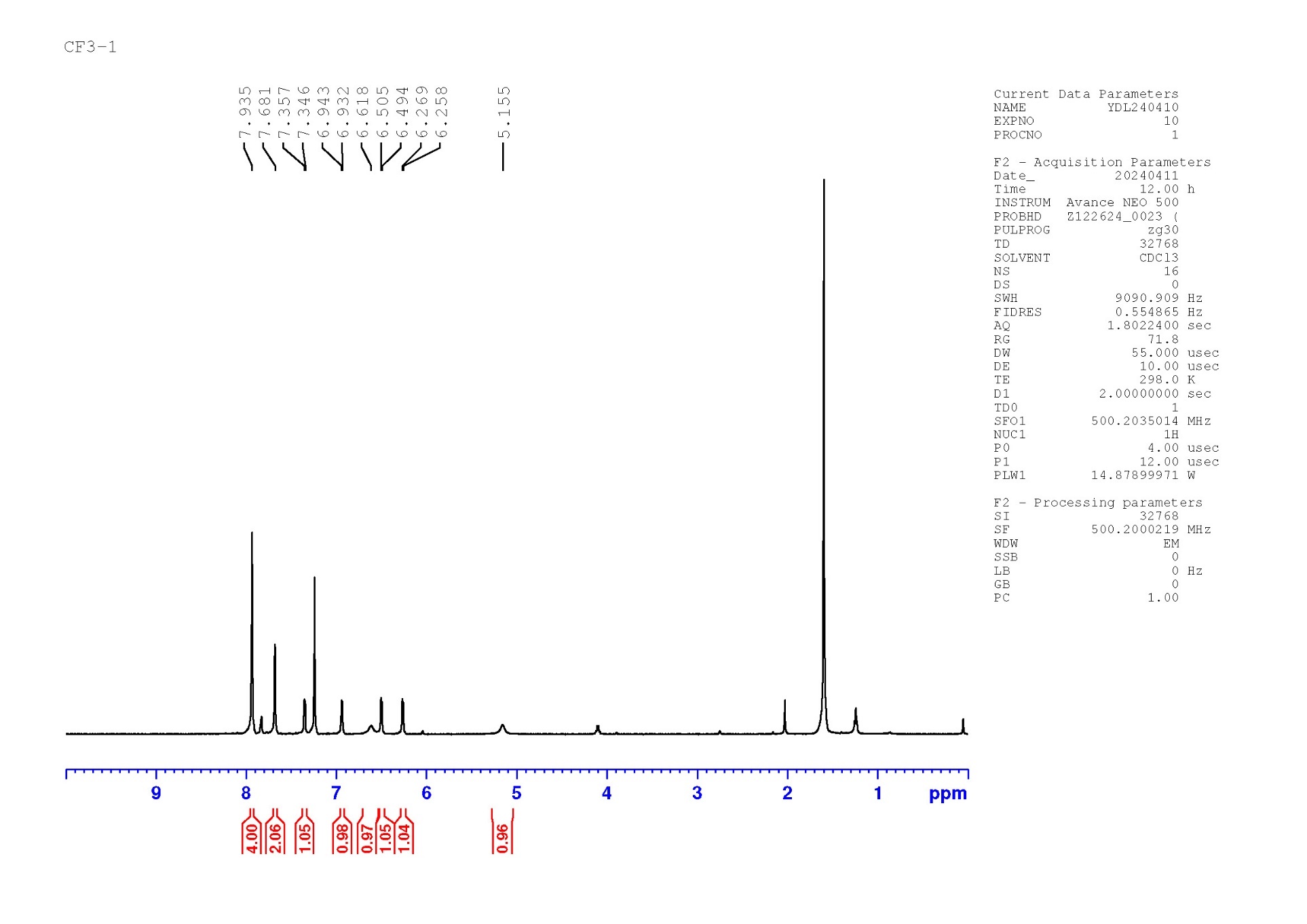


**Figure S5.** ^1^H NMR spectrum of compound **2c**.


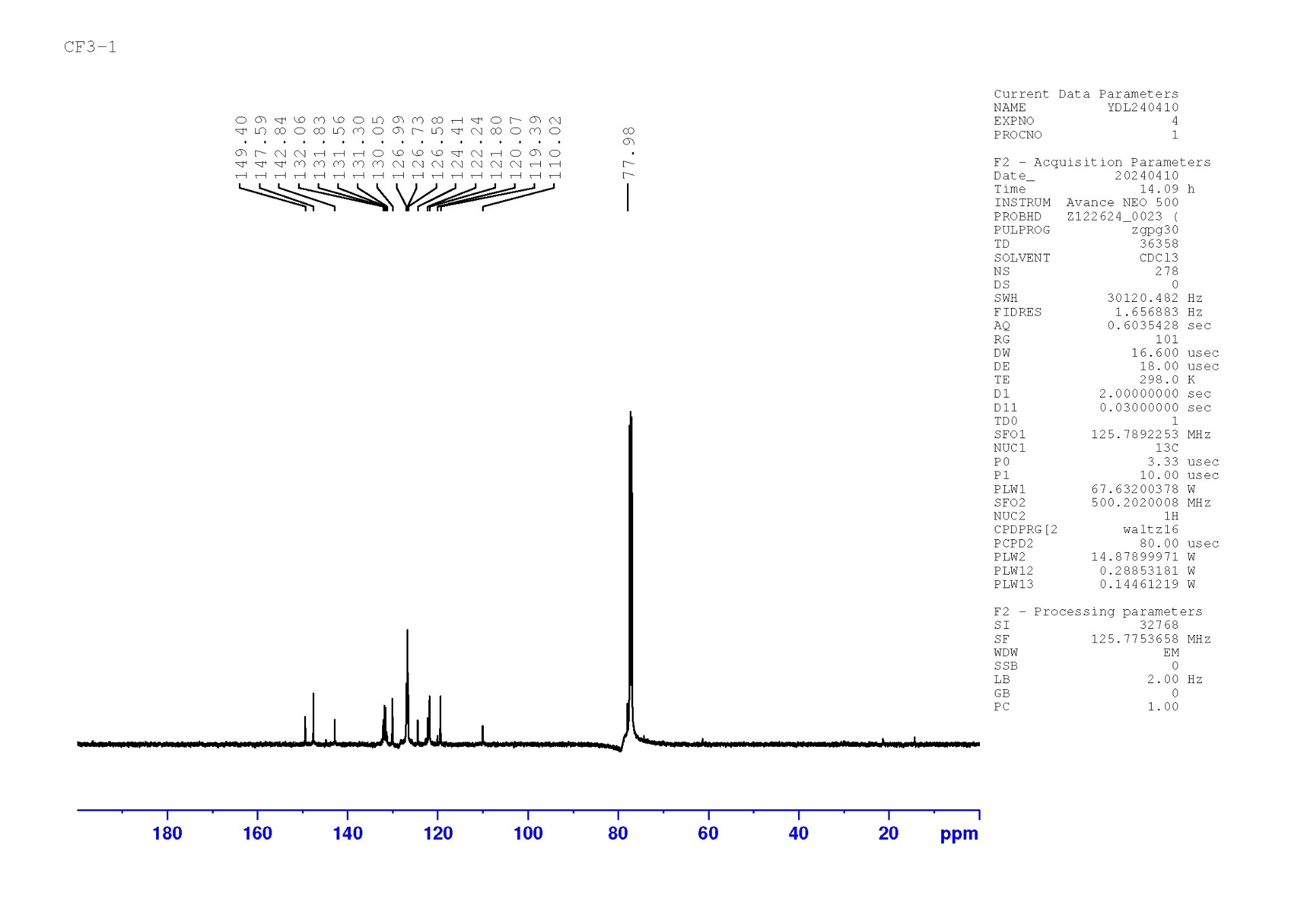


**Figure S6.** ^13^C NMR spectrum of compound **2c**.


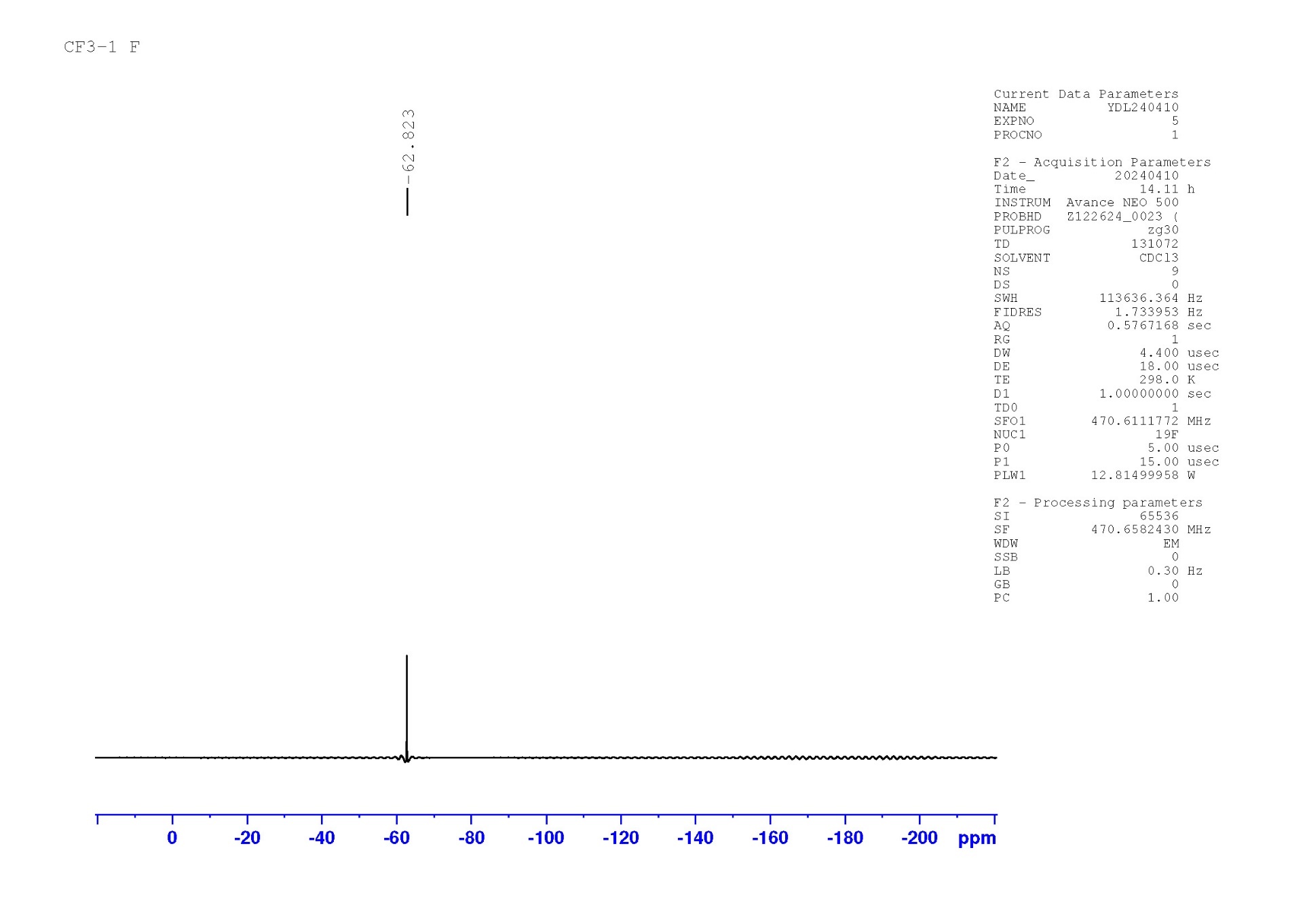


**Figure S7.** ^19^F NMR spectrum of compound **2c**.


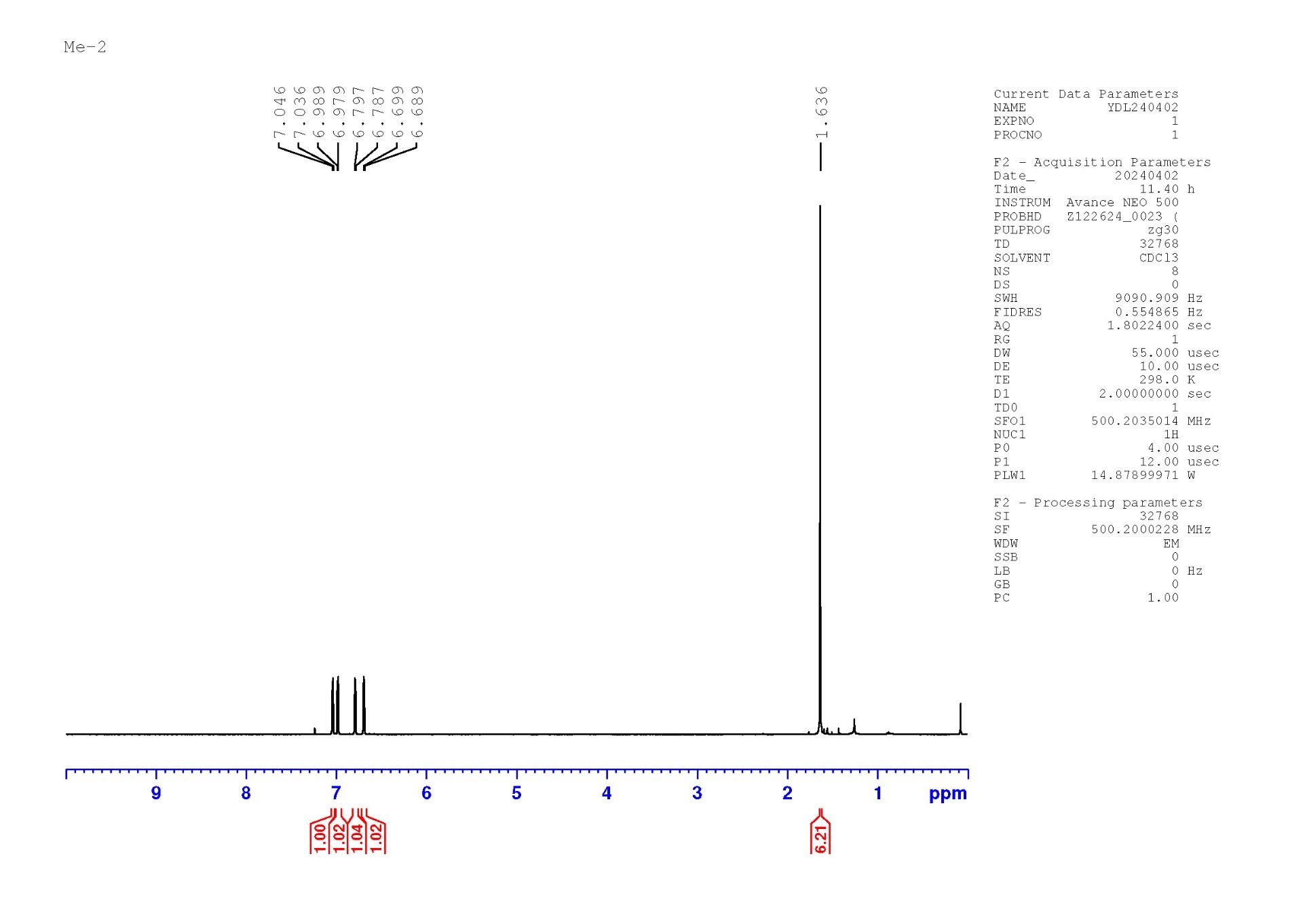


**Figure S8.** ^1^H NMR spectrum of compound **3a**.

**
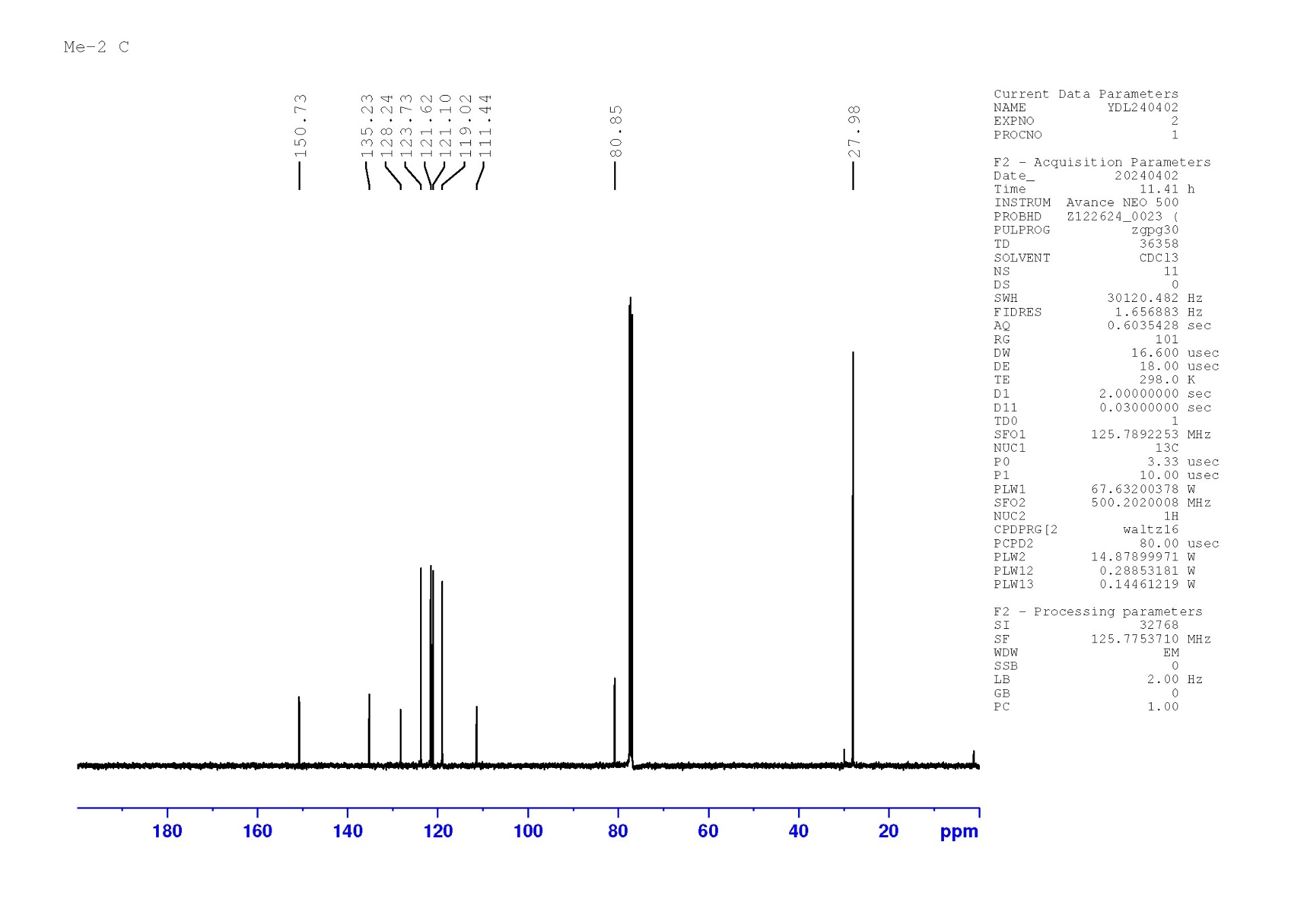
**

**Figure S9.** ^13^C NMR spectrum of compound **3a**.

**
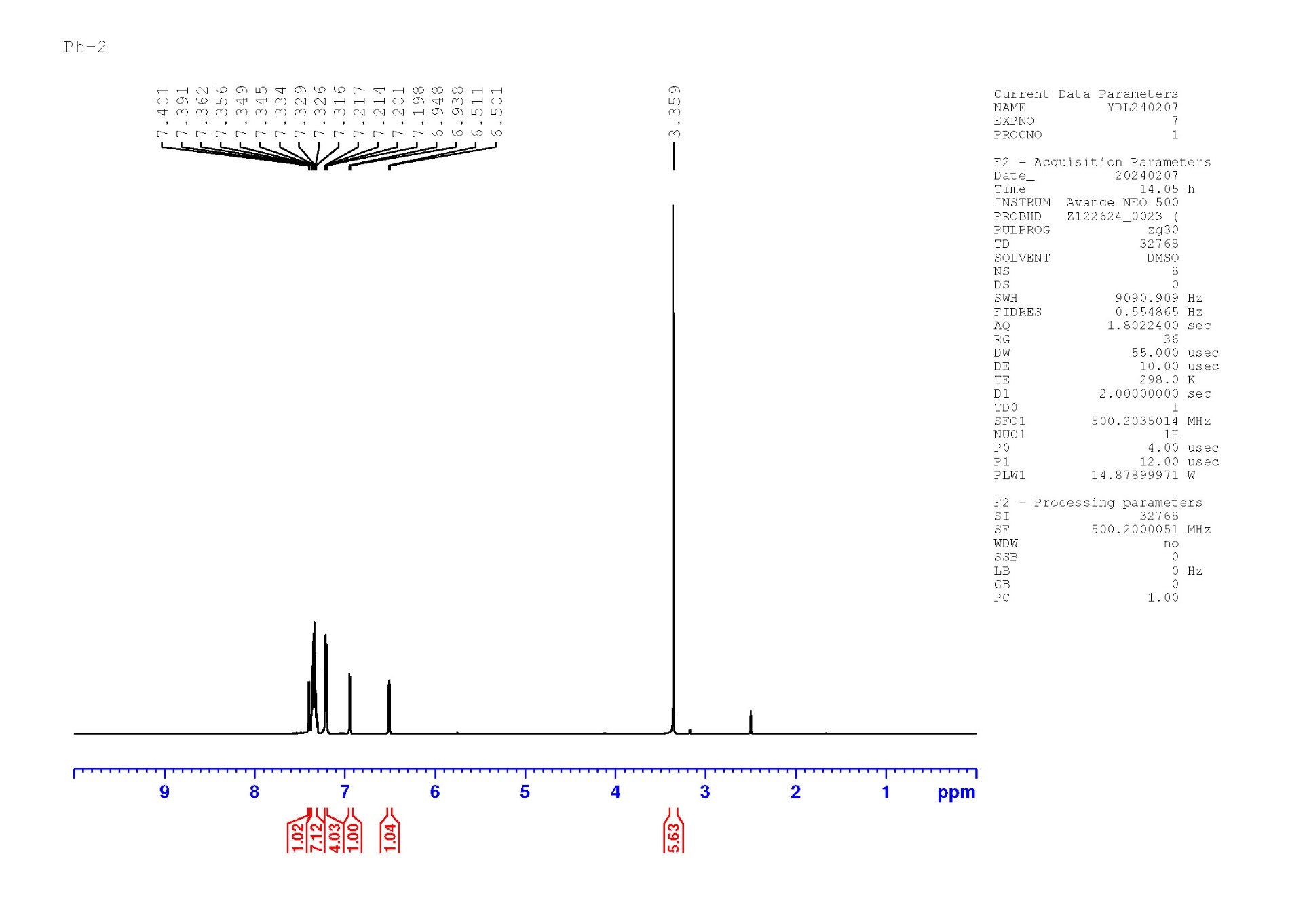
**

**Figure S10.** ^1^H NMR spectrum of compound **3b**.


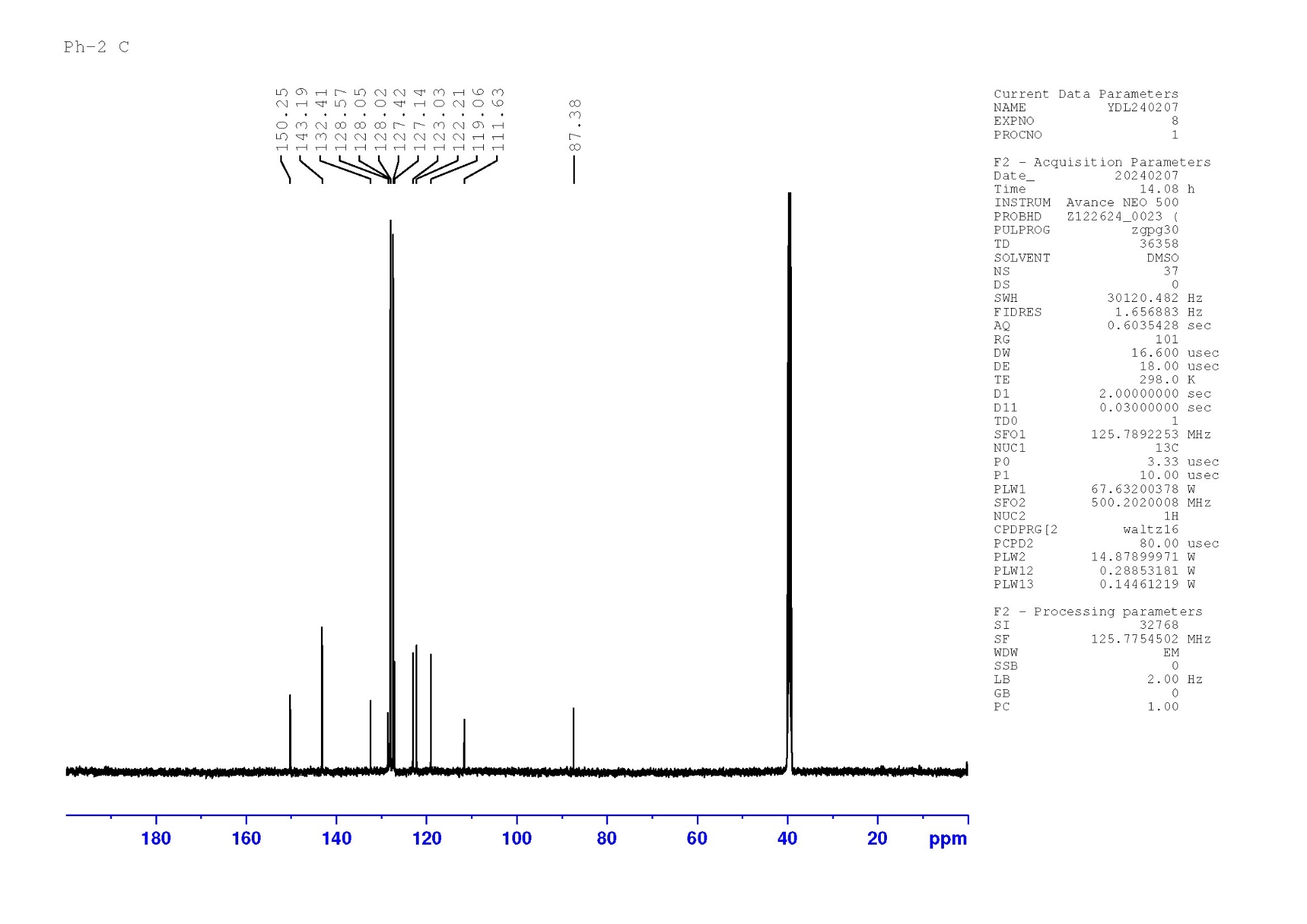


**Figure S11.** ^13^C NMR spectrum of compound **3b**.


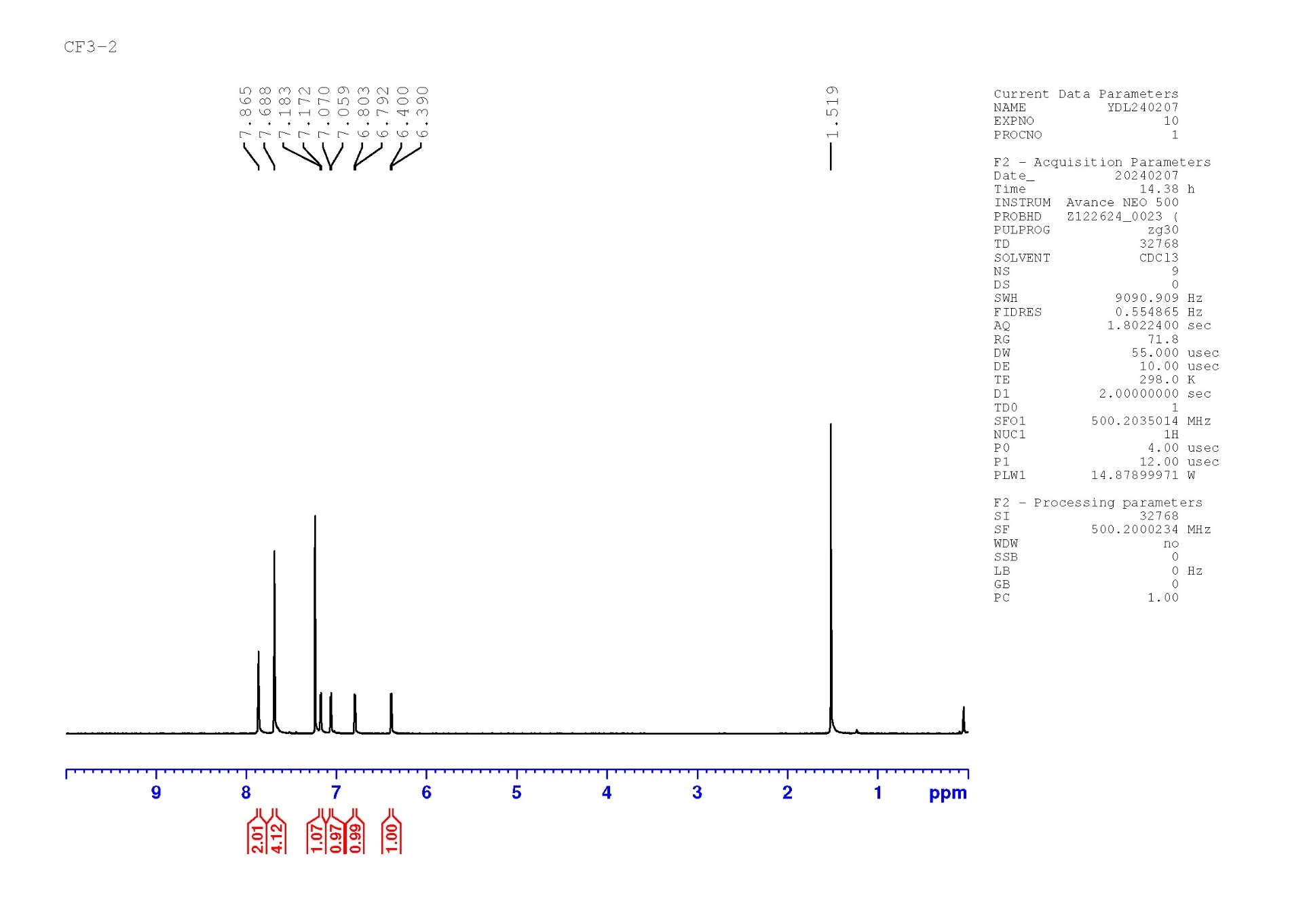


**Figure S12.** ^1^H NMR spectrum of compound **3c**.


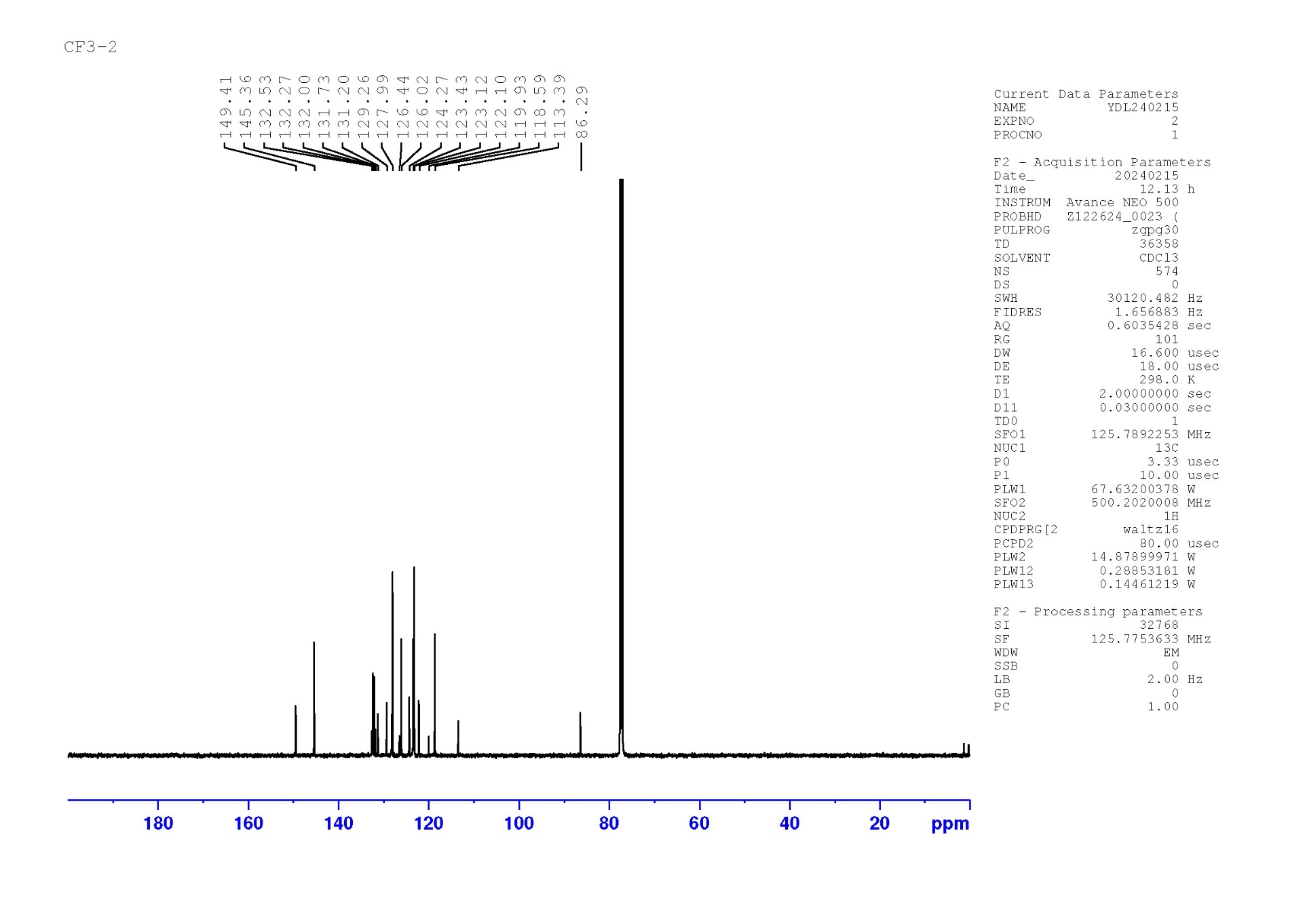


**Figure S13.** ^13^C NMR spectrum of compound **3c**.


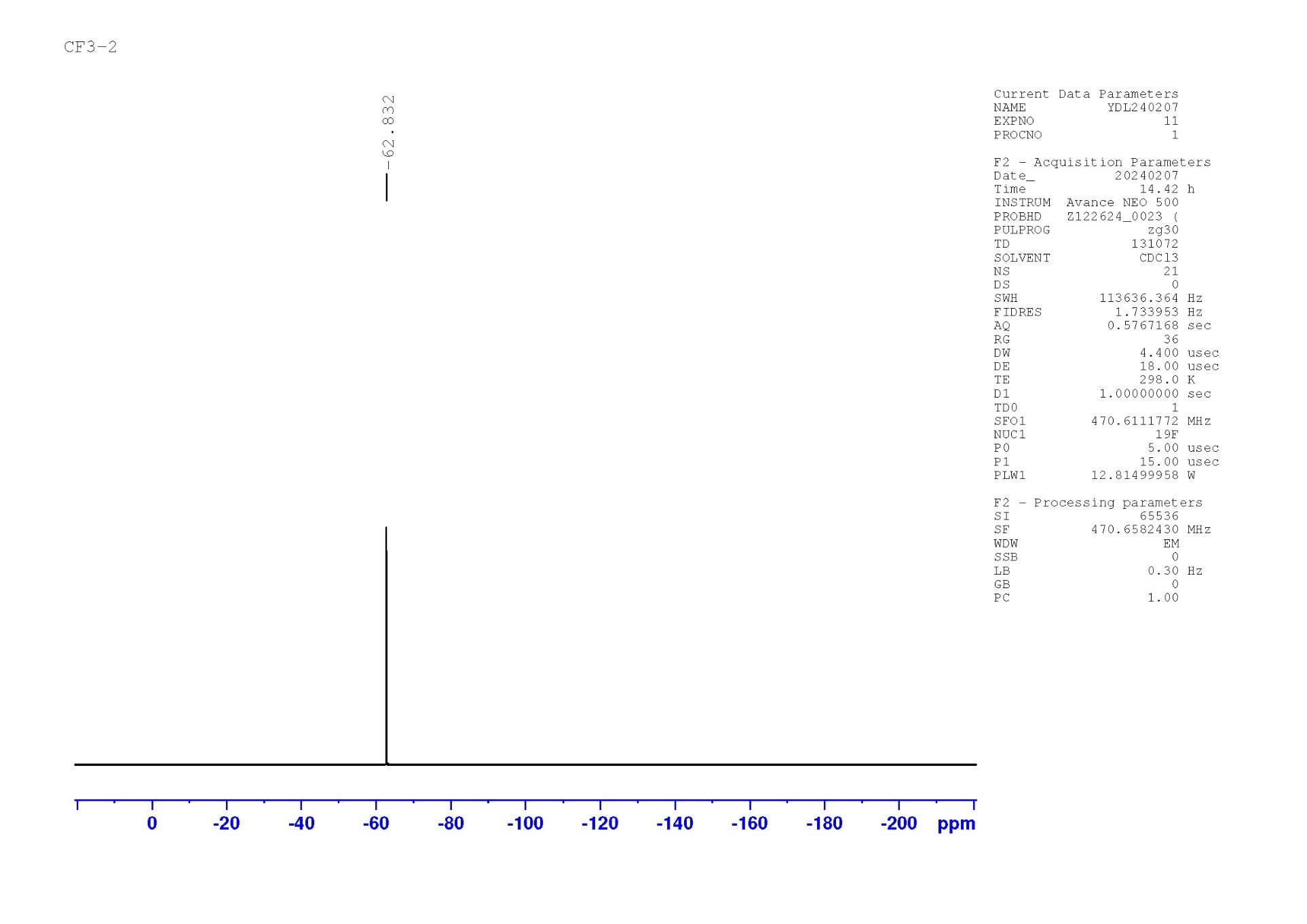


**Figure S14.** ^19^F NMR spectrum of compound **3c**.


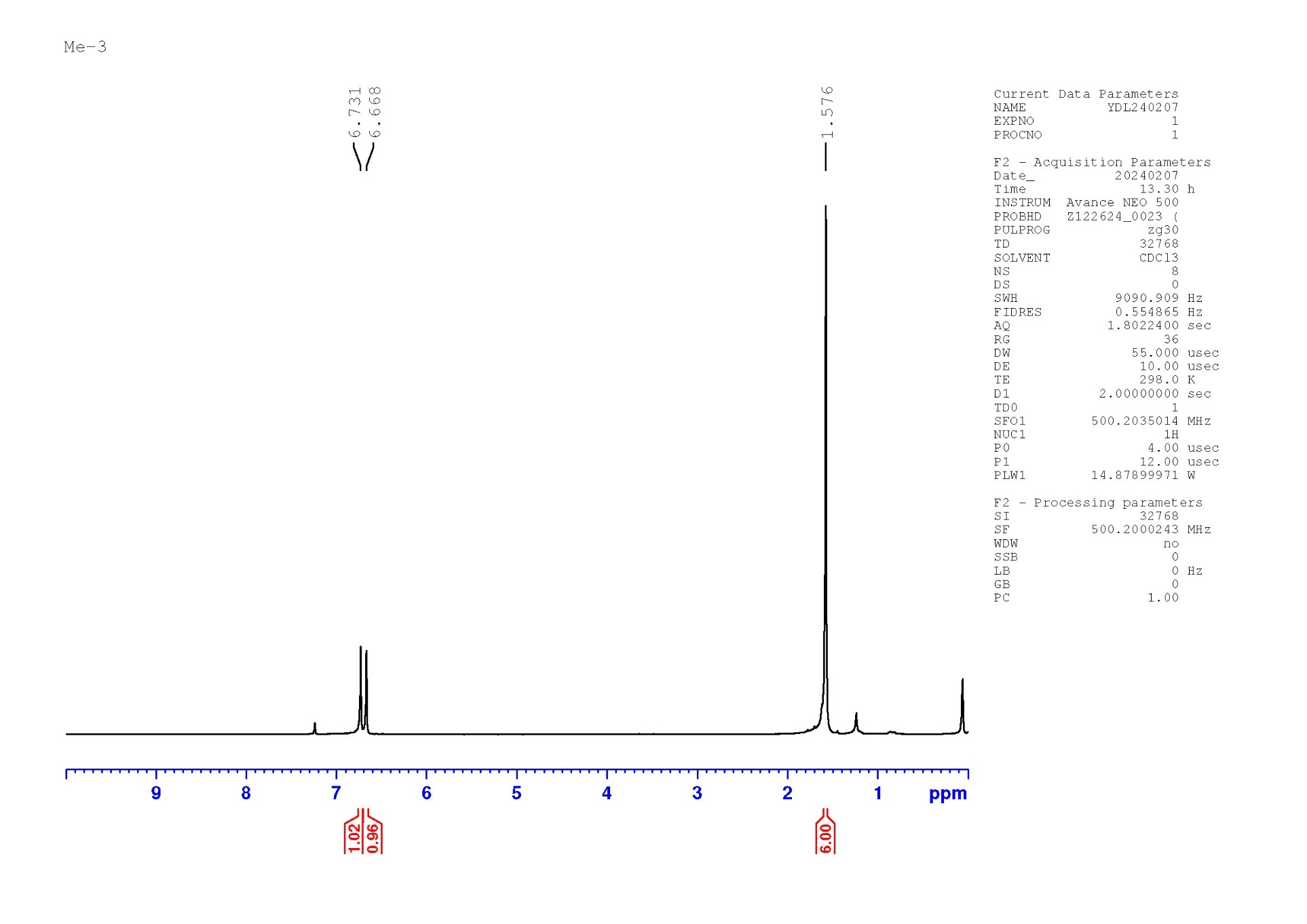


**Figure S15.** ^1^H NMR spectrum of compound **4a**.


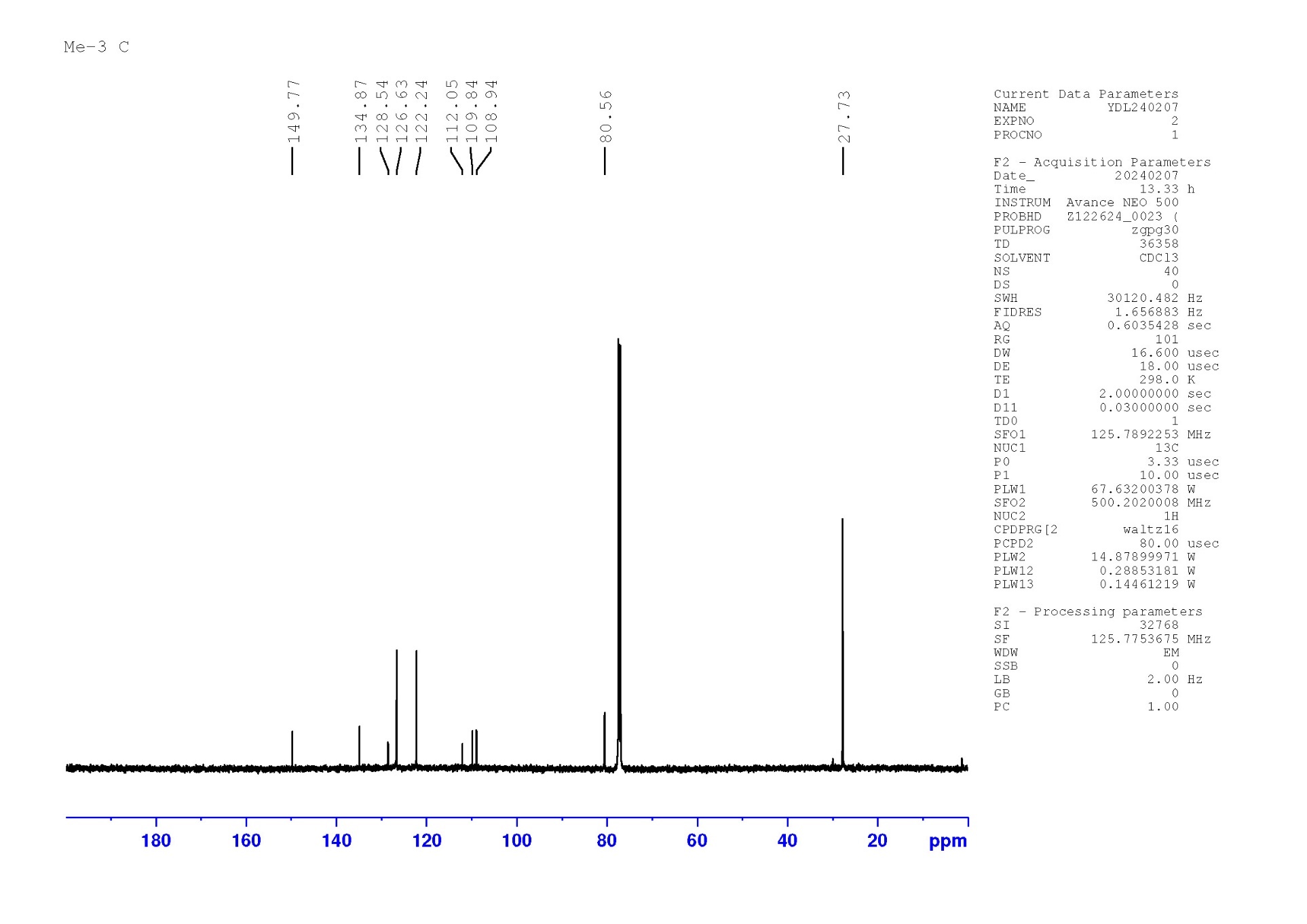


**Figure S16.** ^13^C NMR spectrum of compound **4a**.


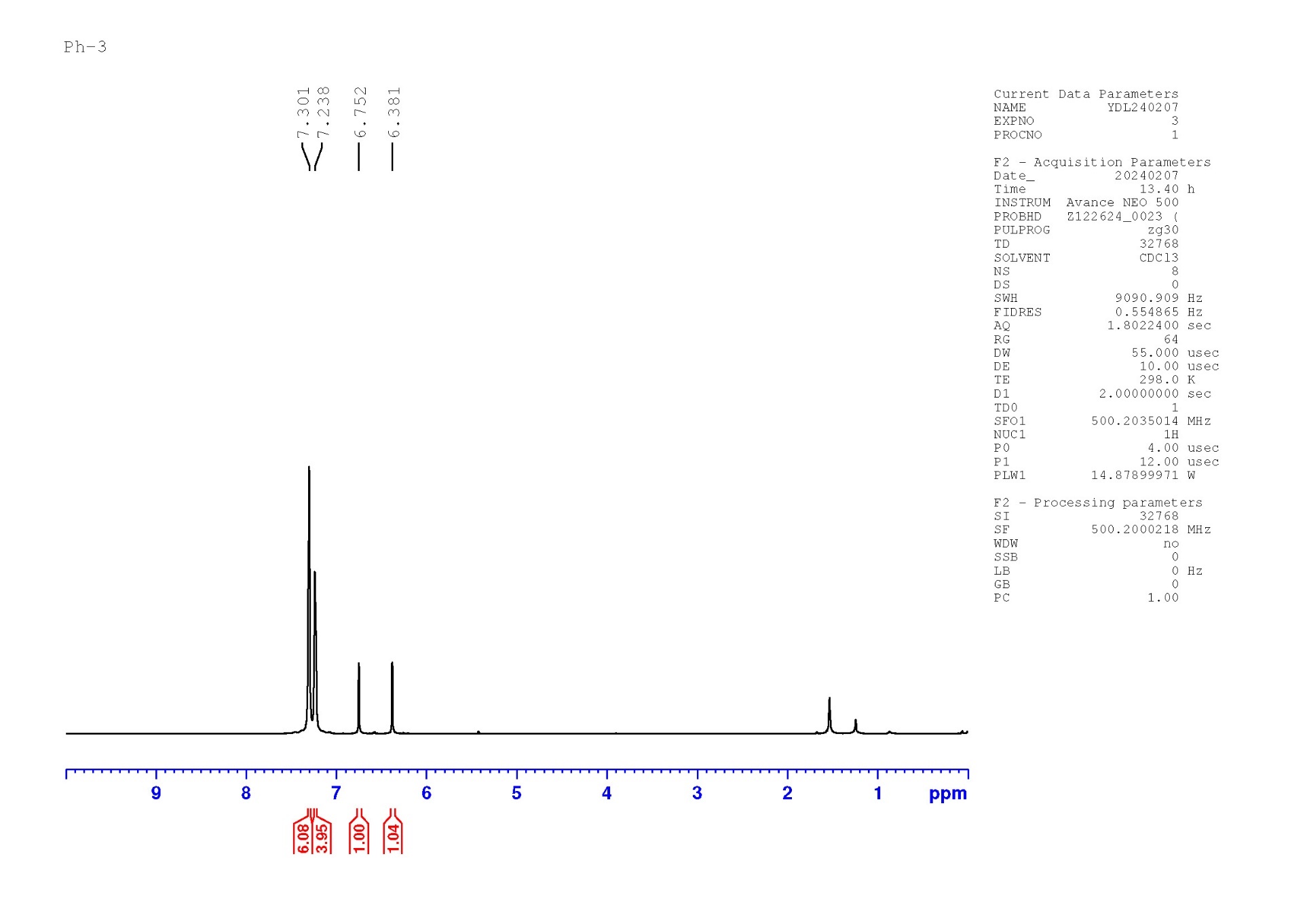


**Figure S17.** ^1^H NMR spectrum of compound **4b**.


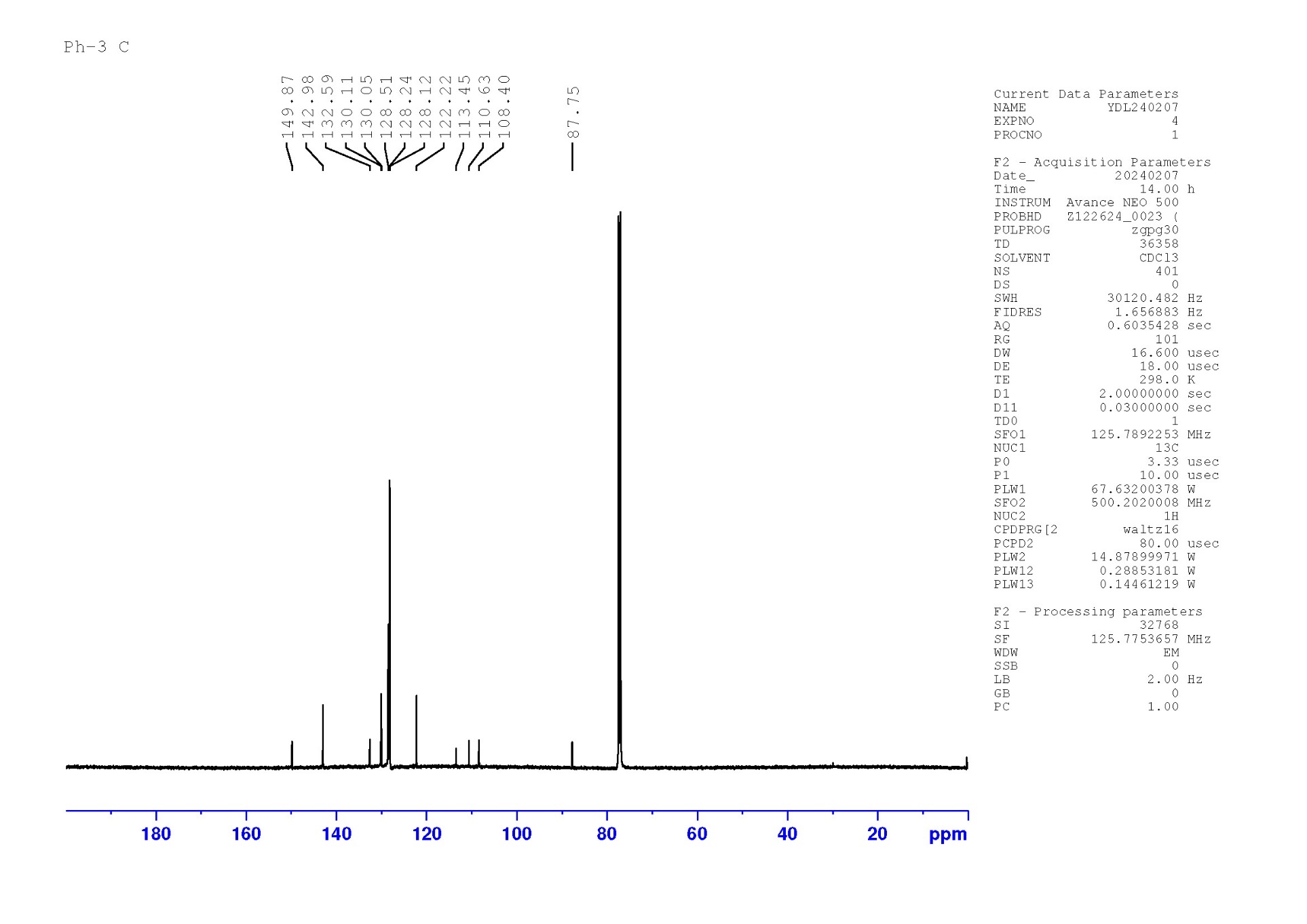


**Figure S18.** ^13^C NMR spectrum of compound **4b**.


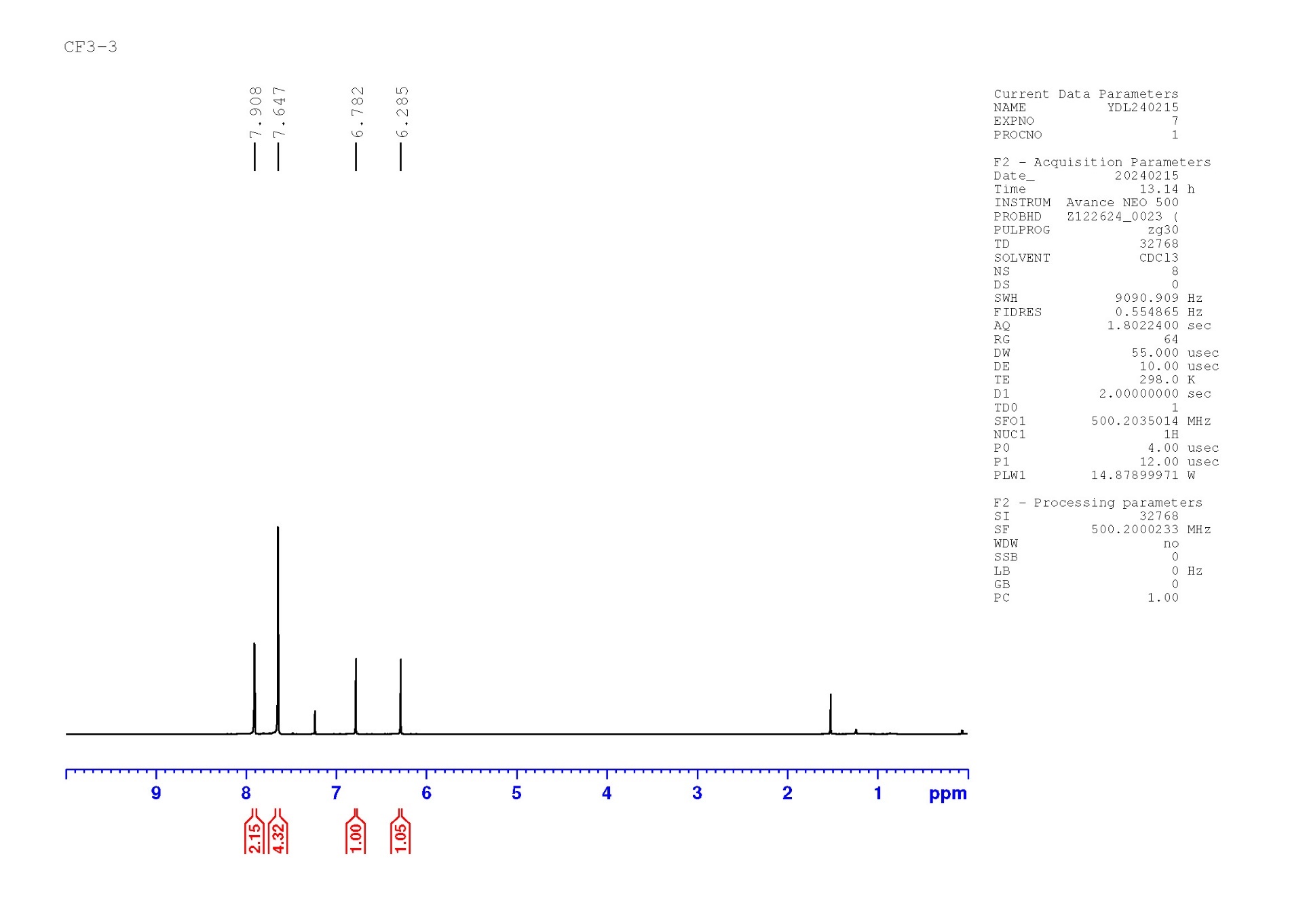


**Figure S19.** ^1^H NMR spectrum of compound **4c**.


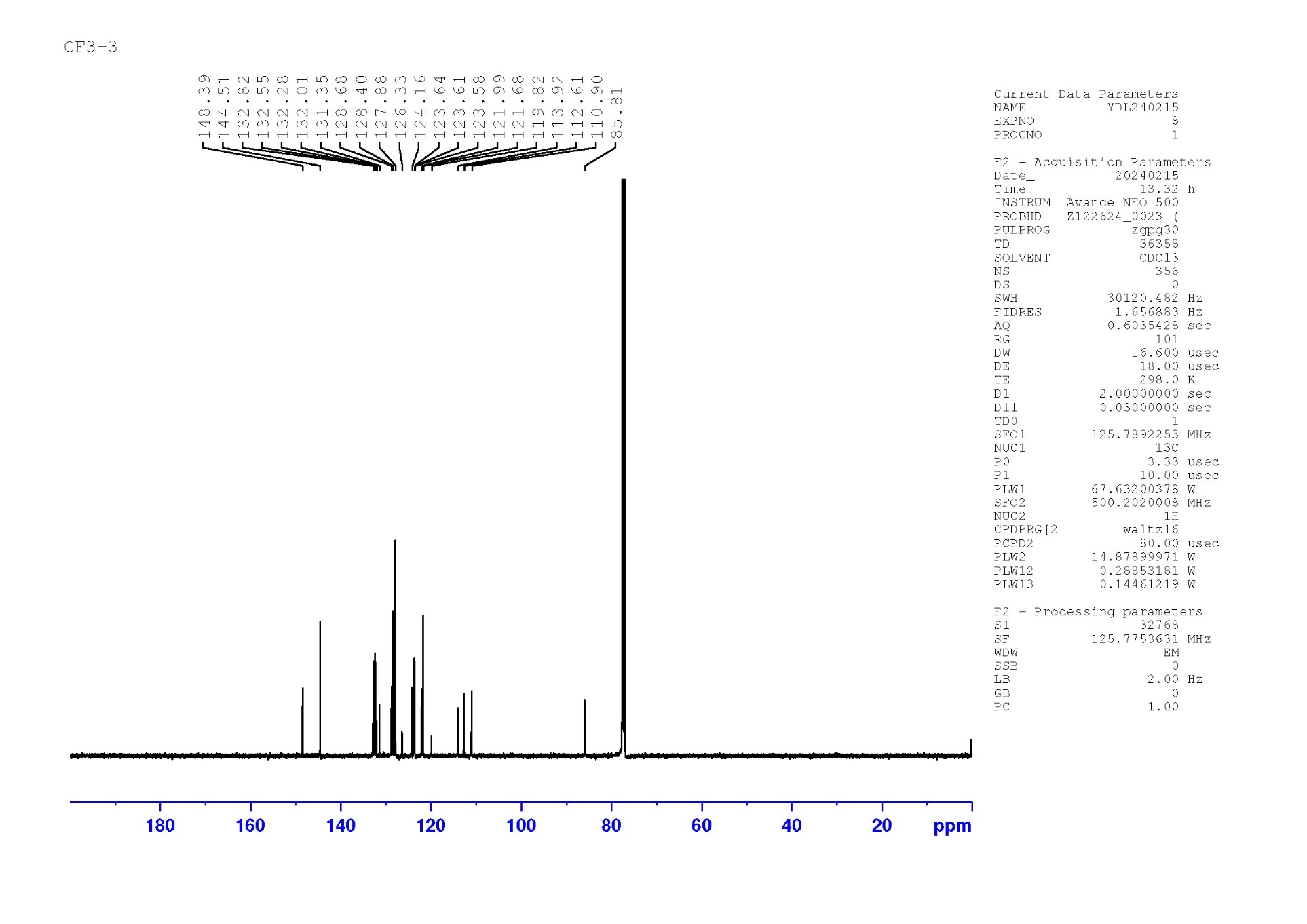


**Figure S20.** ^13^C NMR spectrum of compound **4c**.


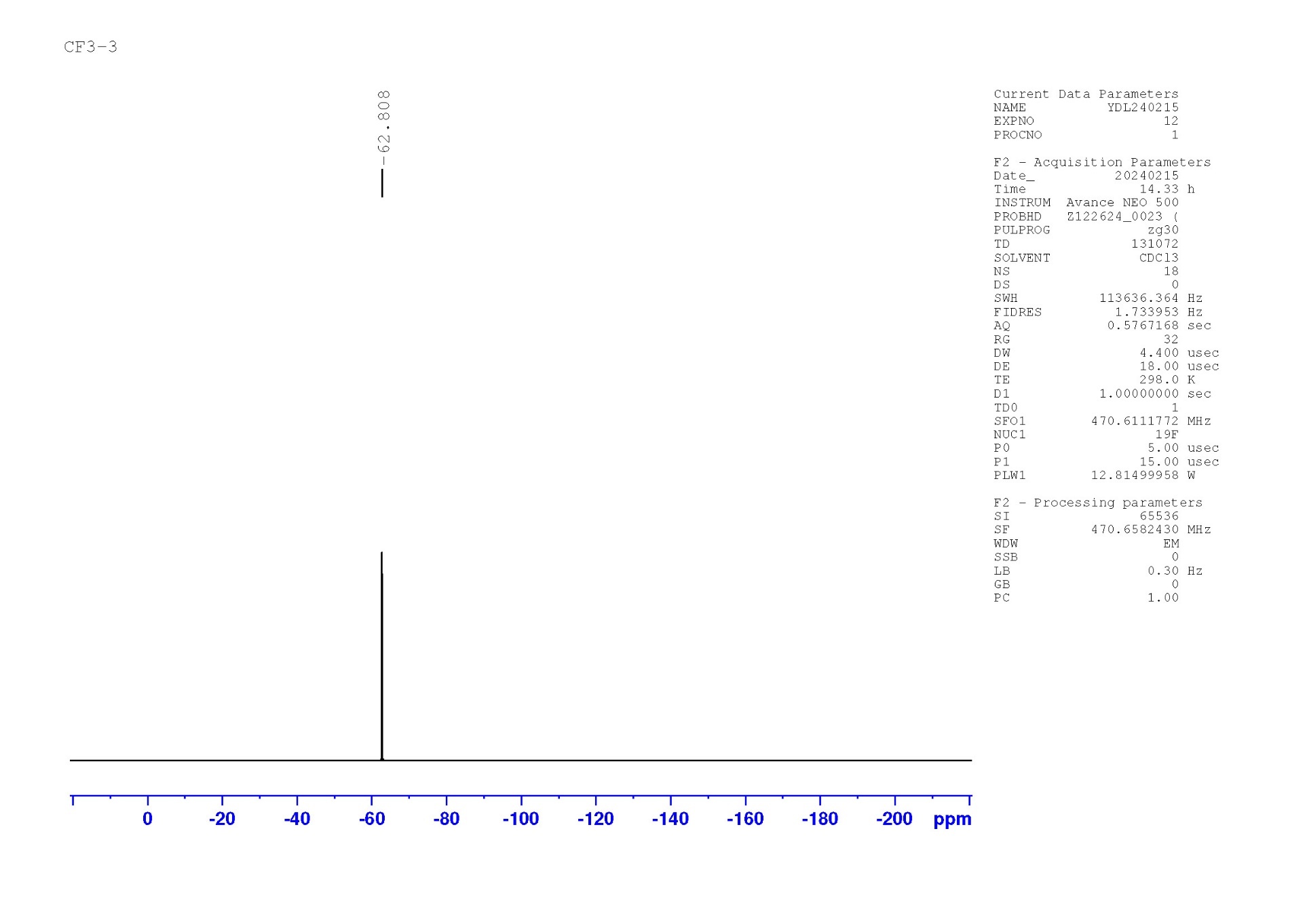


**Figure S21.** ^19^F NMR spectrum of compound **4c**.


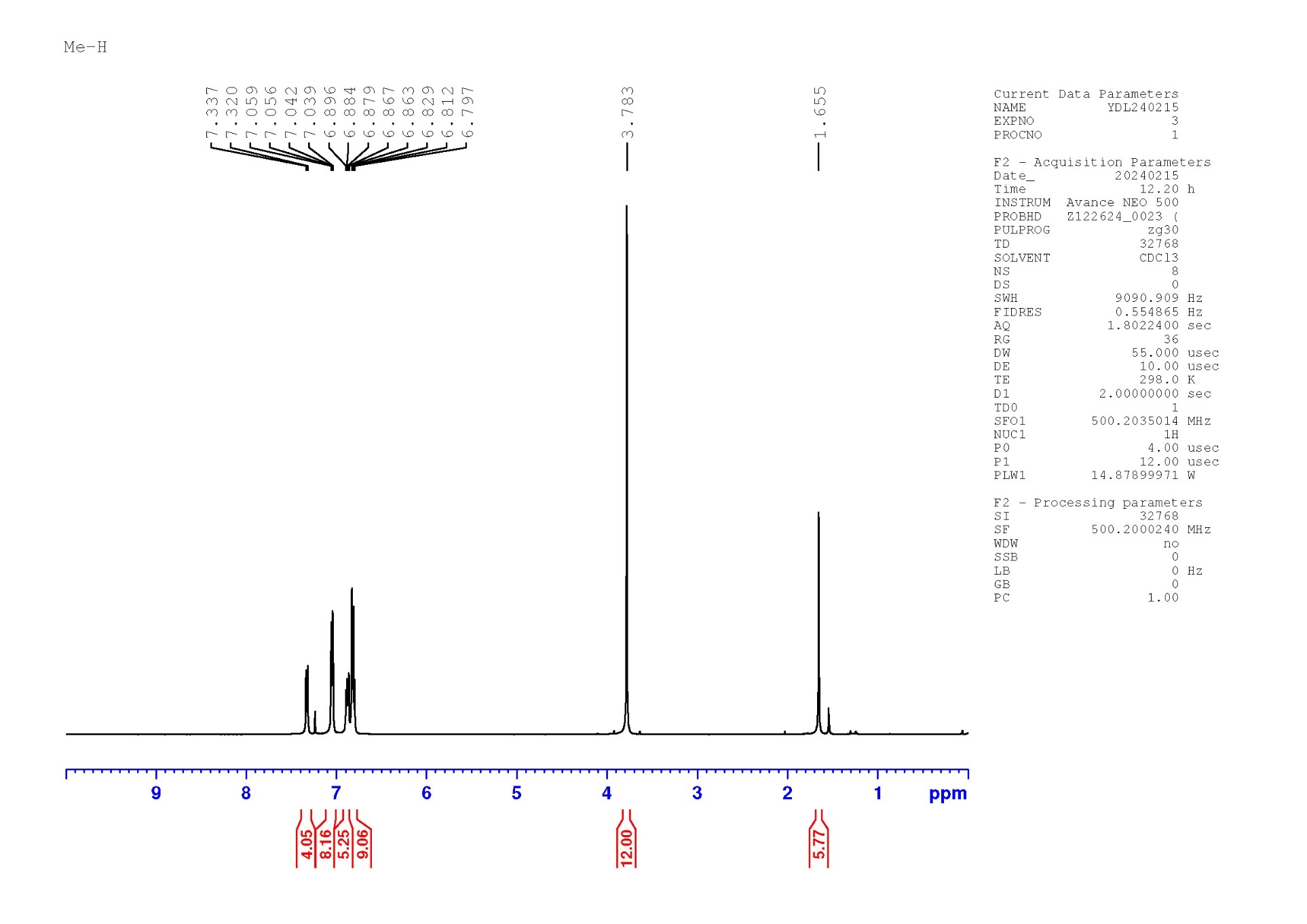


**Figure S22.** ^1^H NMR spectrum of compound **Me-H**.


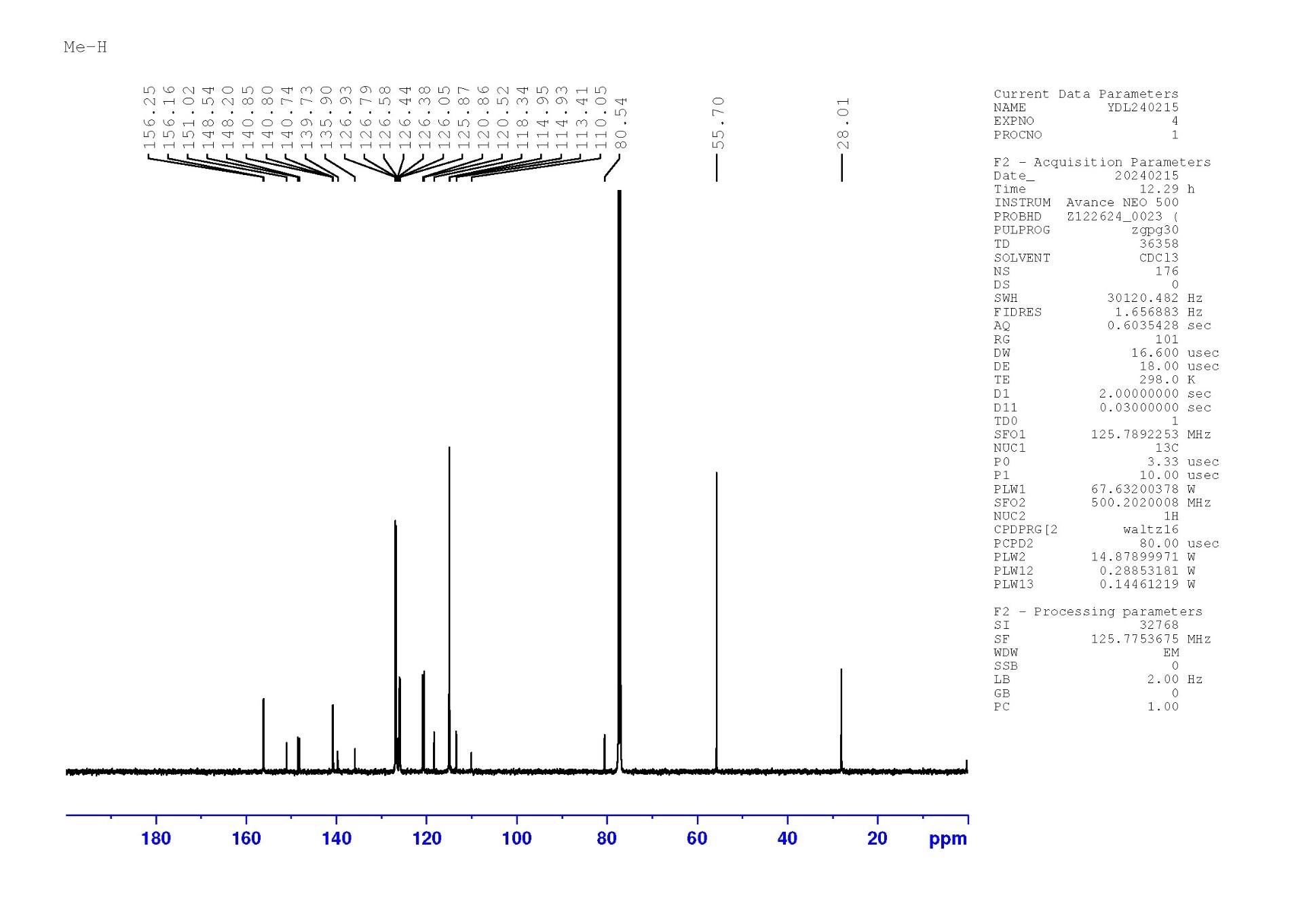


**Figure S23.** ^13^C NMR spectrum of compound **Me-H**.


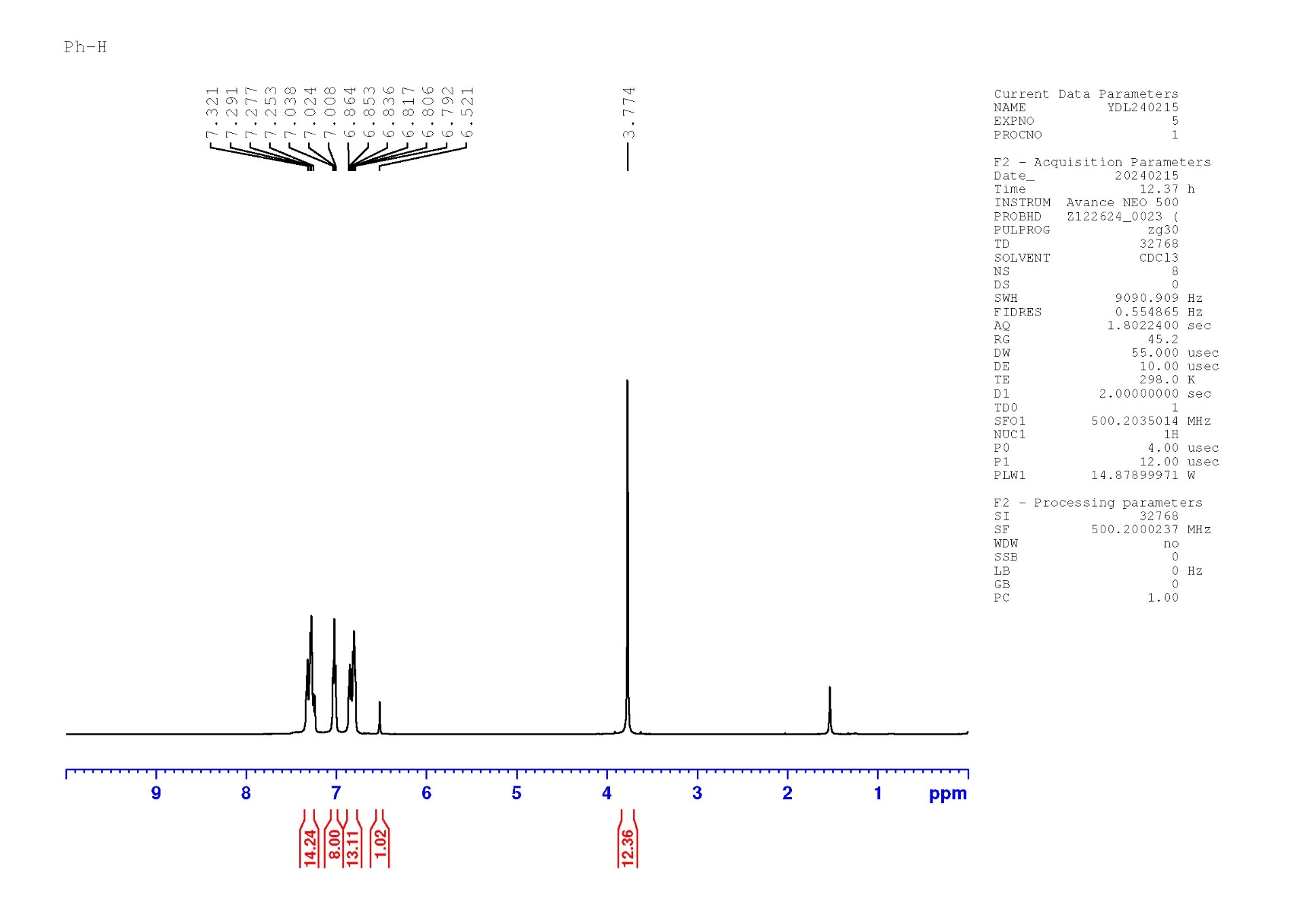


**Figure S24.** ^1^H NMR spectrum of compound **Ph-H**.


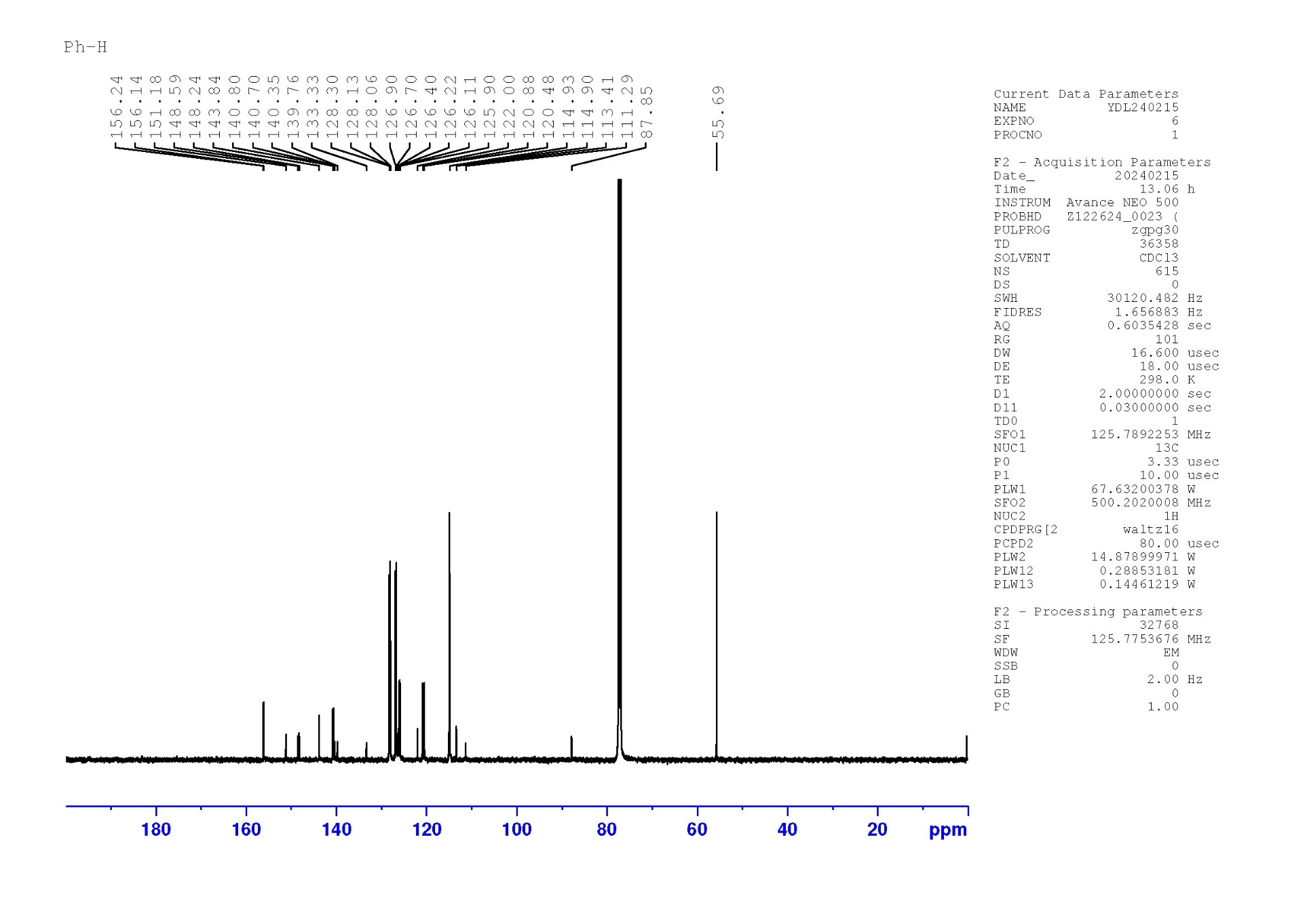


**Figure S25.** ^13^C NMR spectrum of compound **Ph-H**.

**
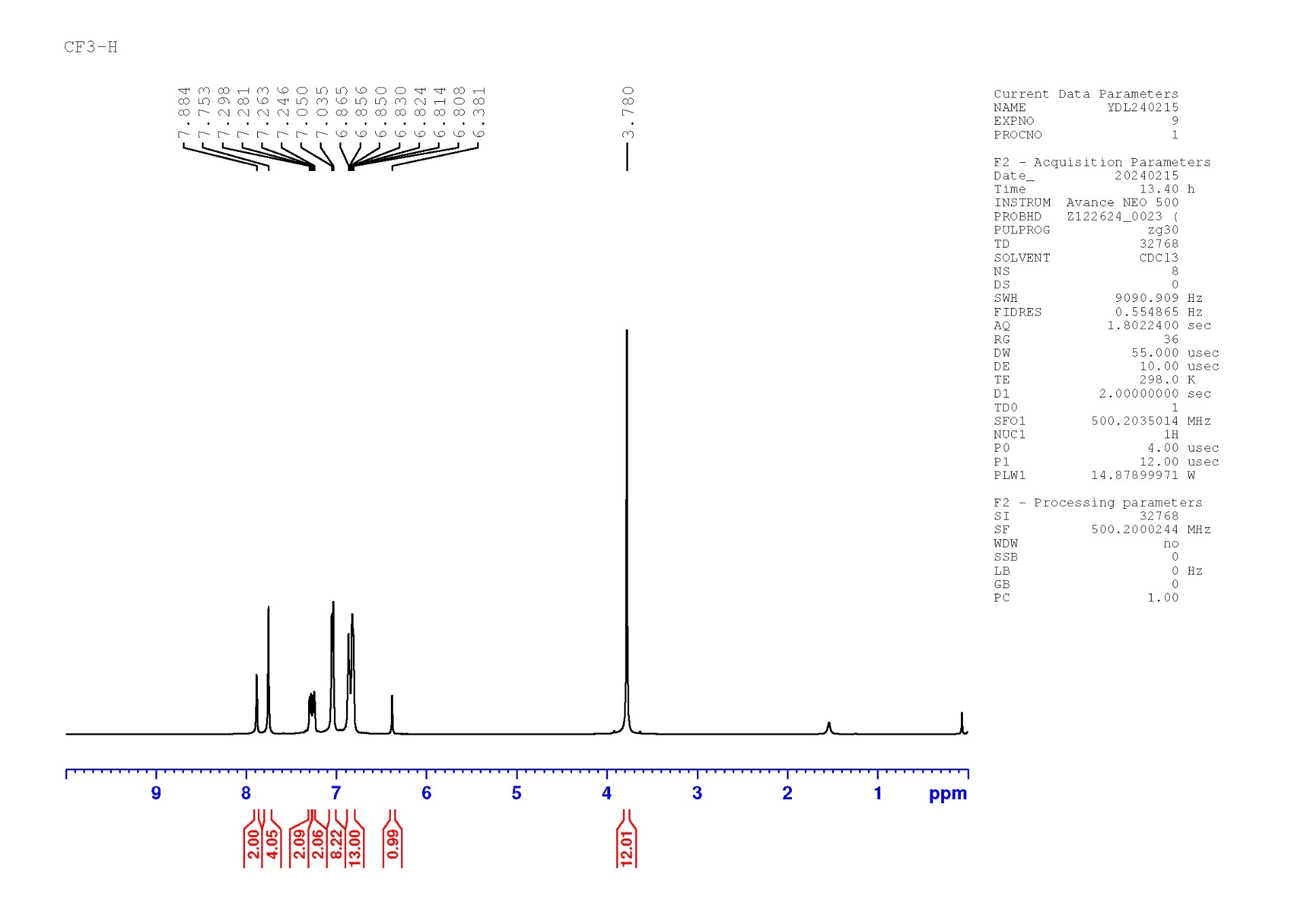
**

**Figure S26.** ^1^H NMR spectrum of compound **CF3-H**.


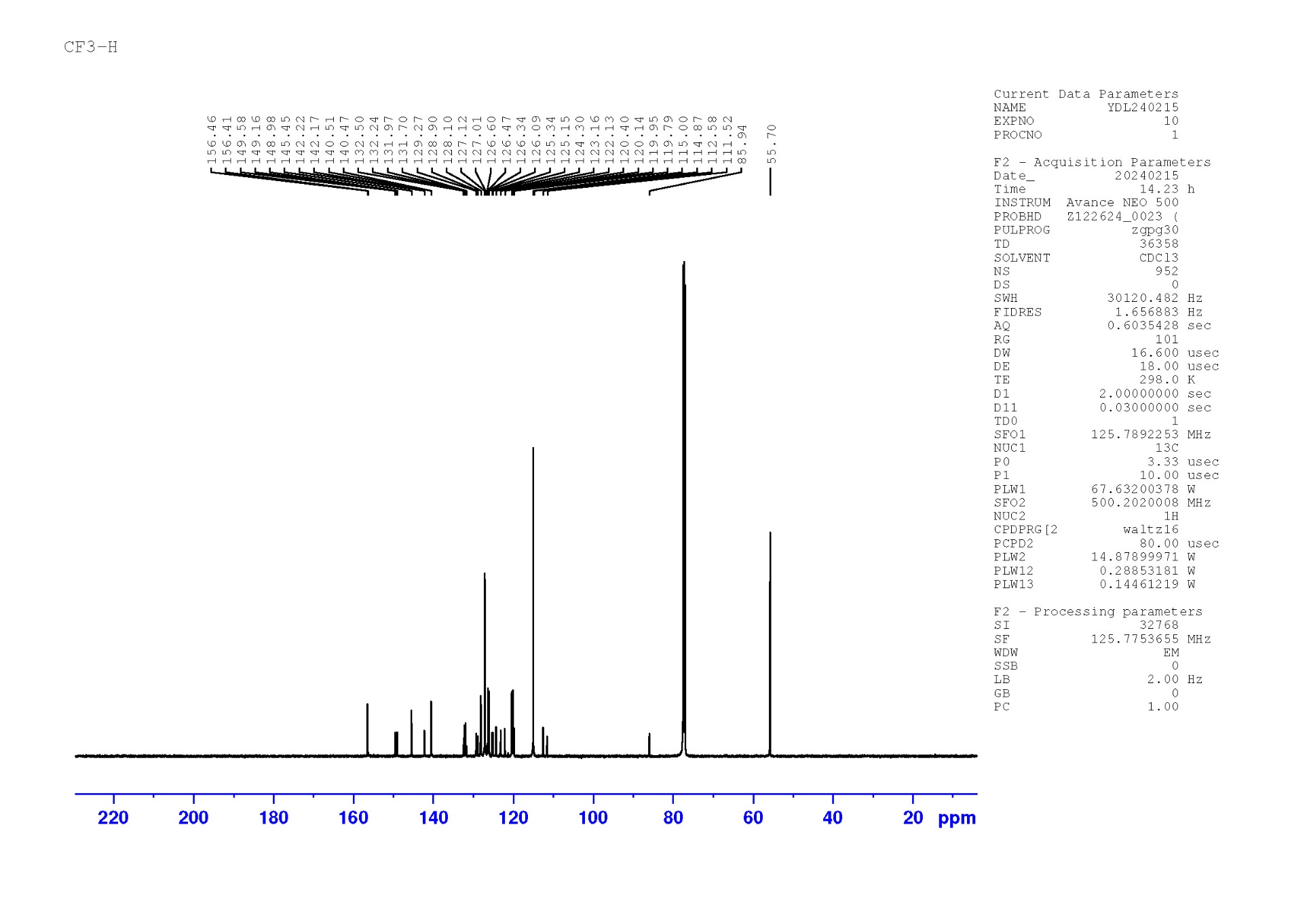


**Figure S27.** ^13^C NMR spectrum of compound **CF3-H**.


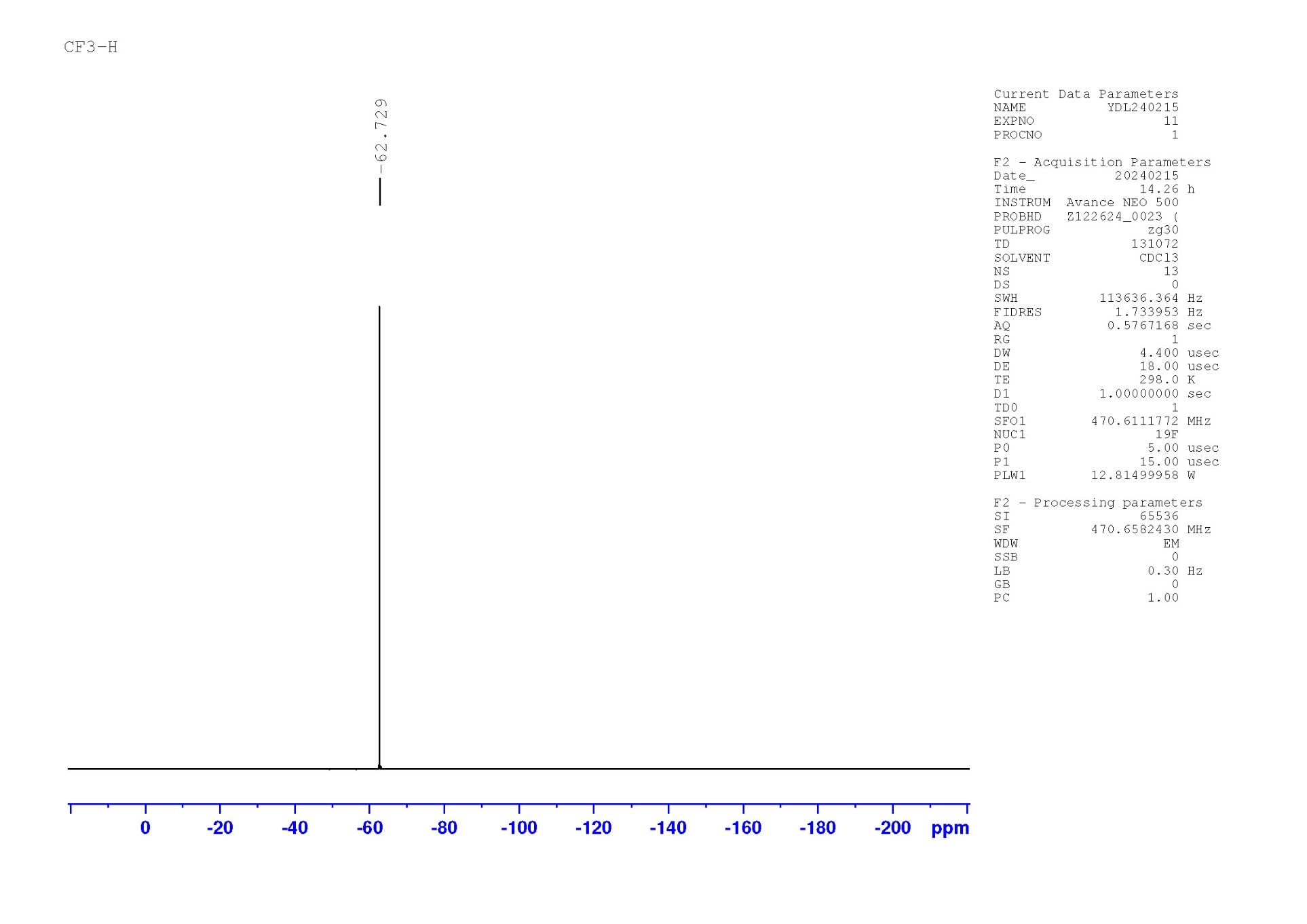


**Figure S28.** ^19^F NMR spectrum of compound **CF3-H**.


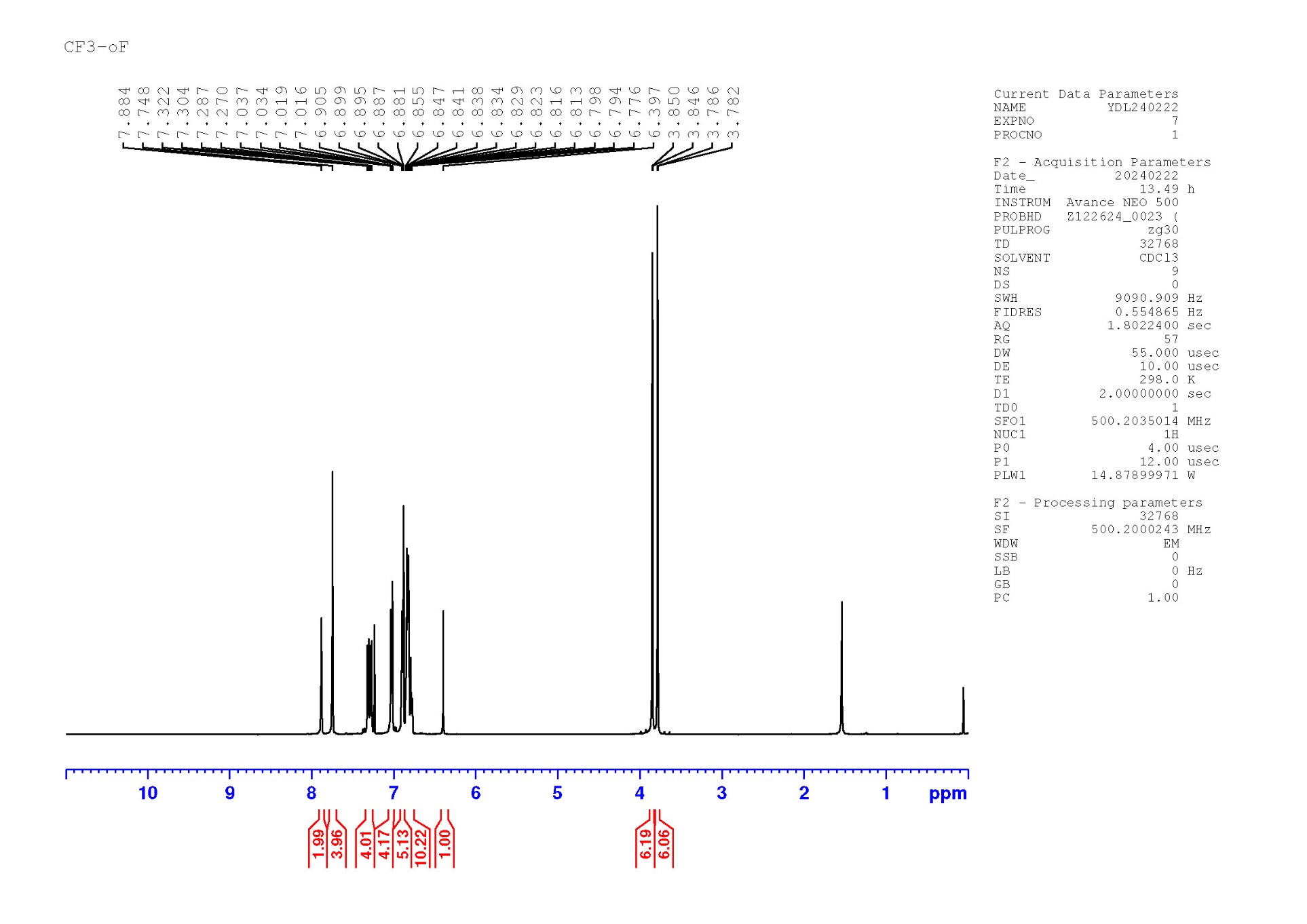


**Figure S29.** ^1^H NMR spectrum of compound **CF3-oF**.


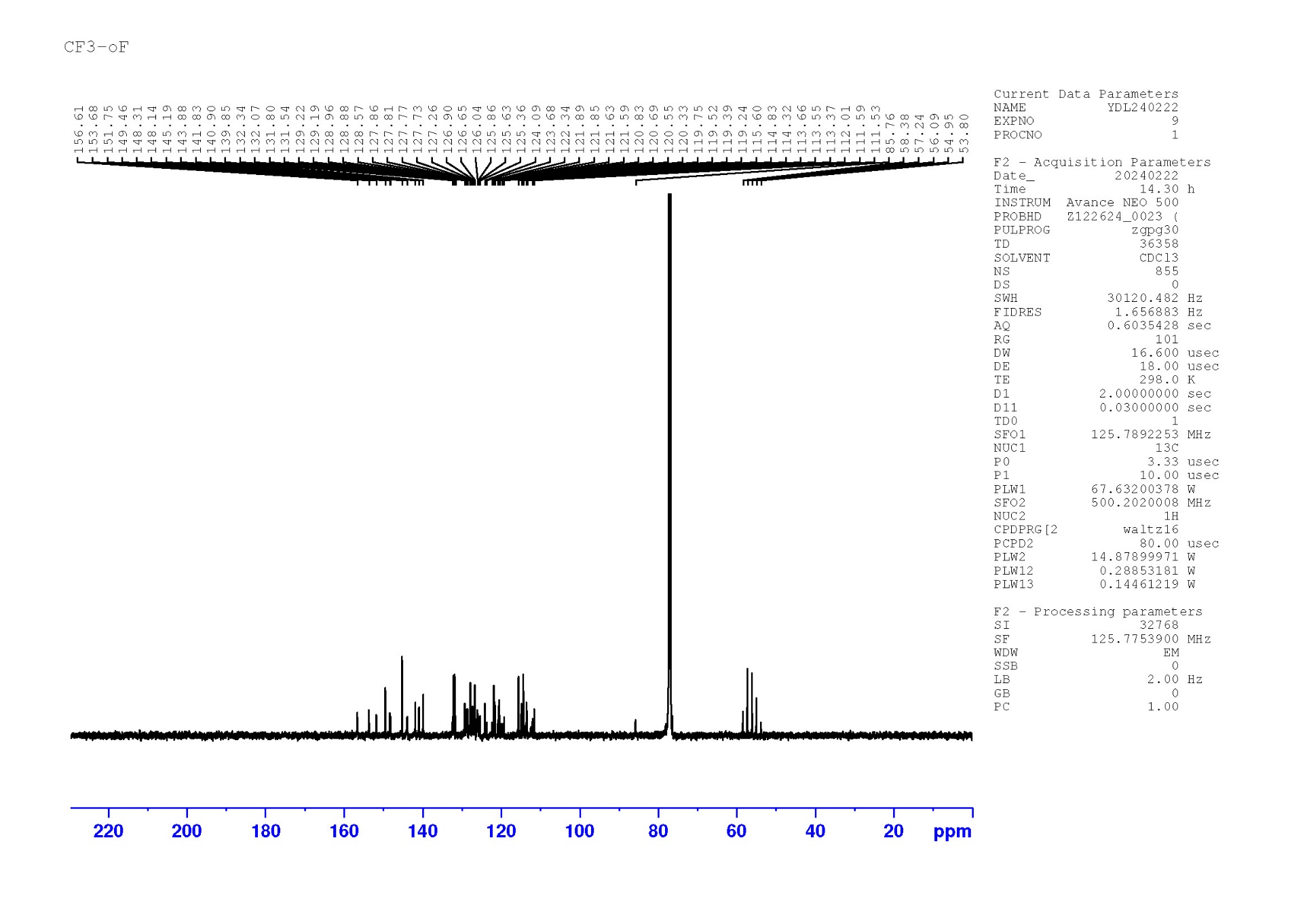


**Figure S30.** ^13^C NMR spectrum of compound **CF3-oF**.


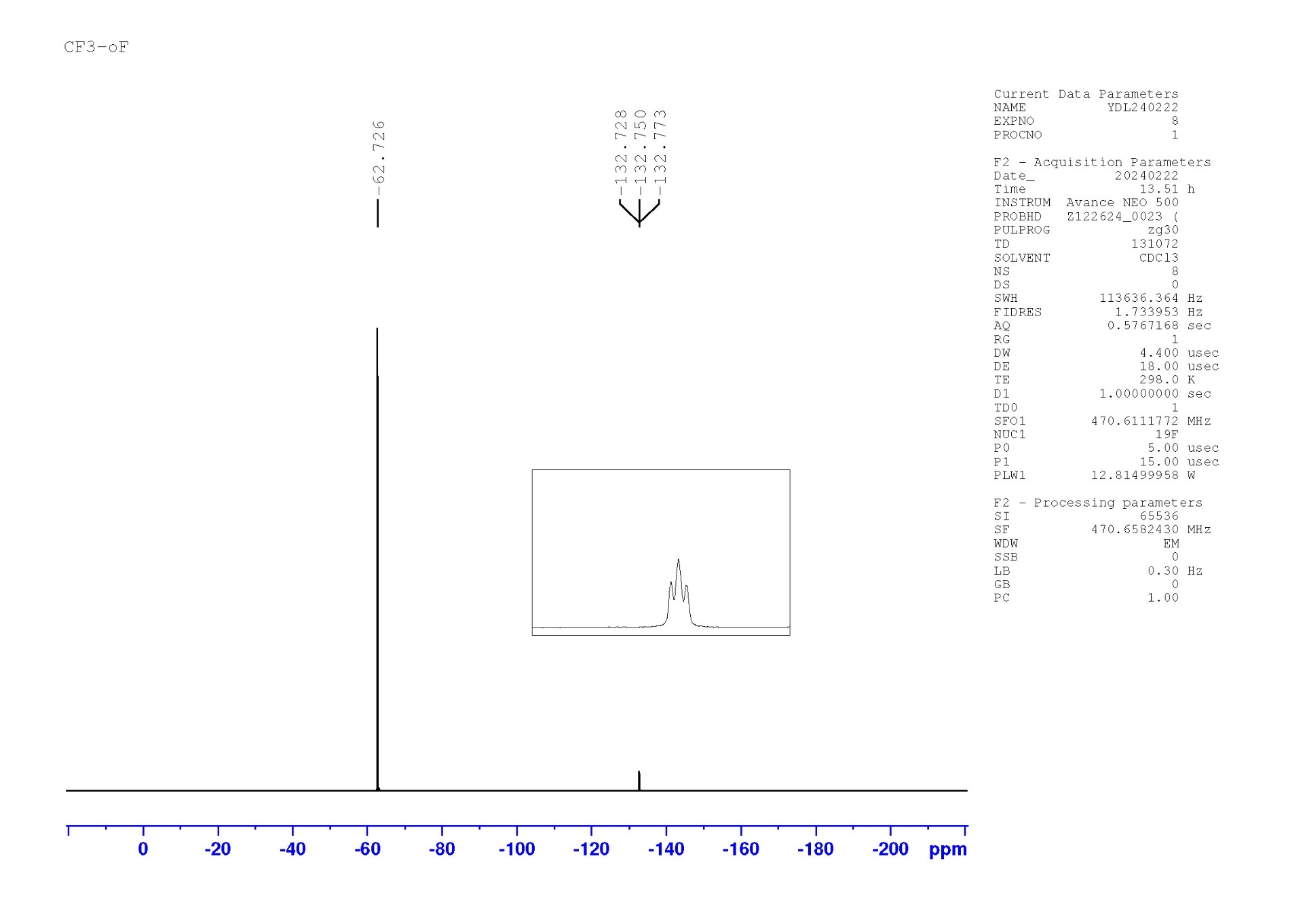


**Figure S31.** ^19^F NMR spectrum of compound **CF3-oF**.


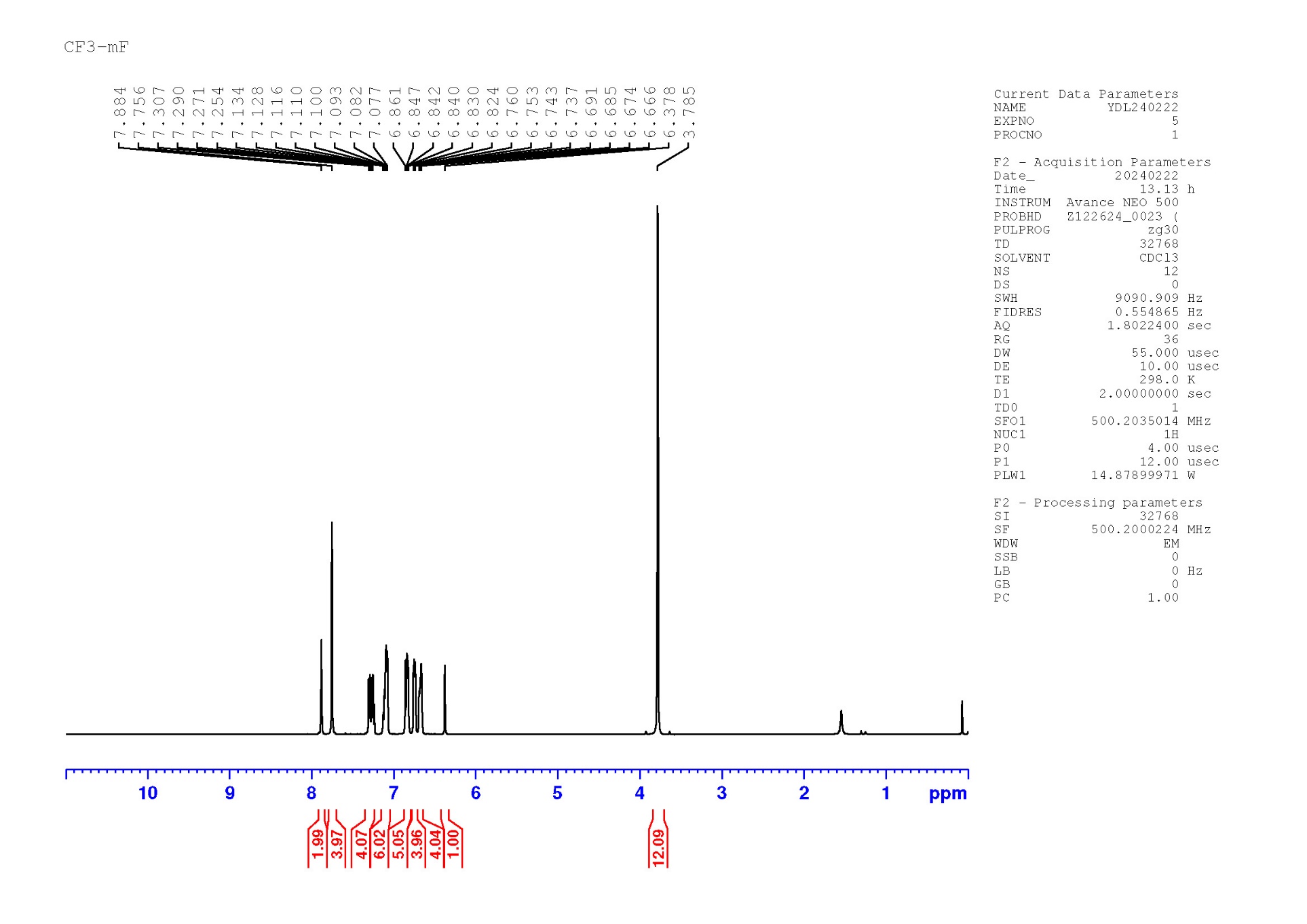


**Figure S32.** ^1^H NMR spectrum of compound **CF3-mF**.


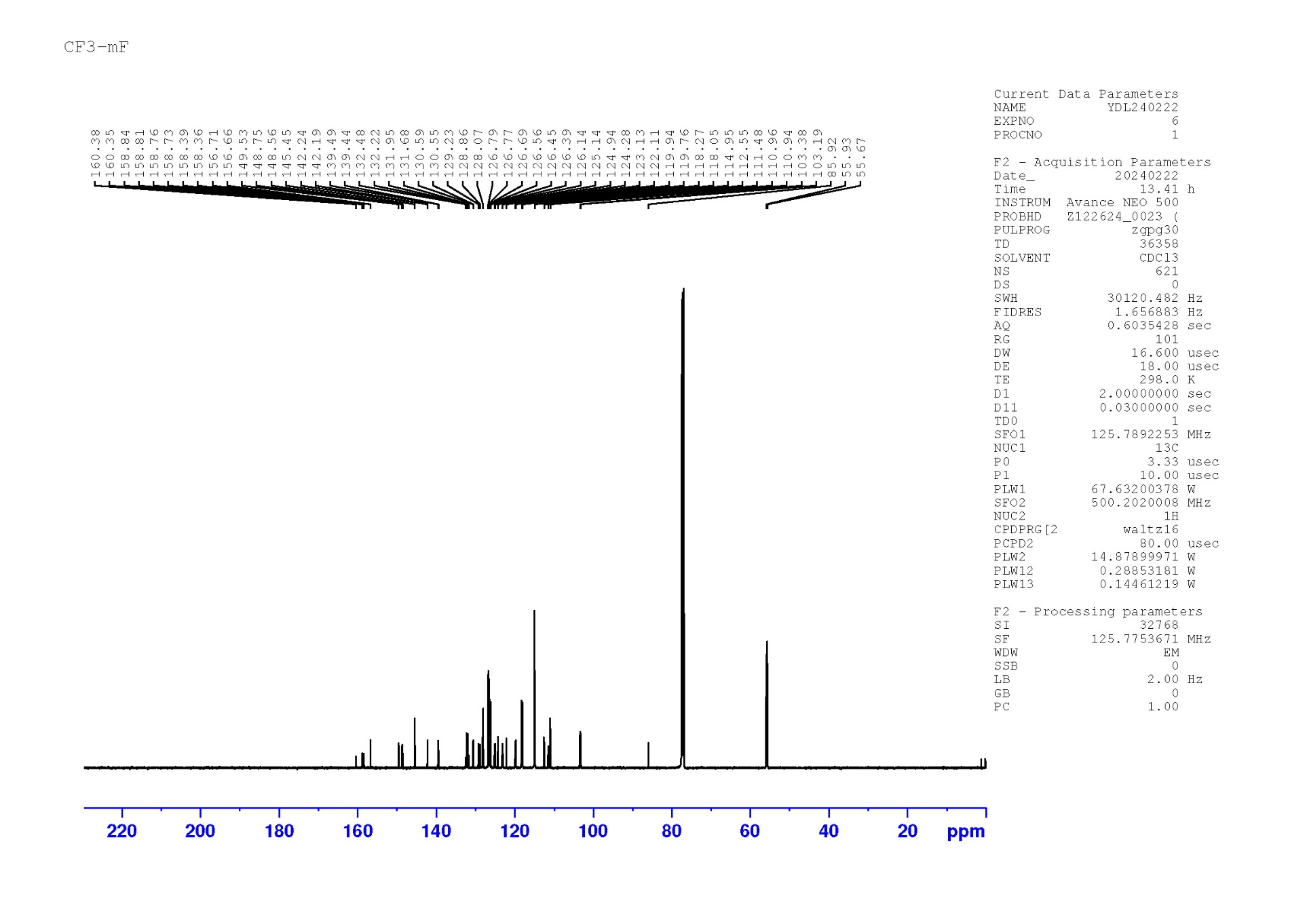


**Figure S33.** ^13^C NMR spectrum of compound **CF3-mF**.


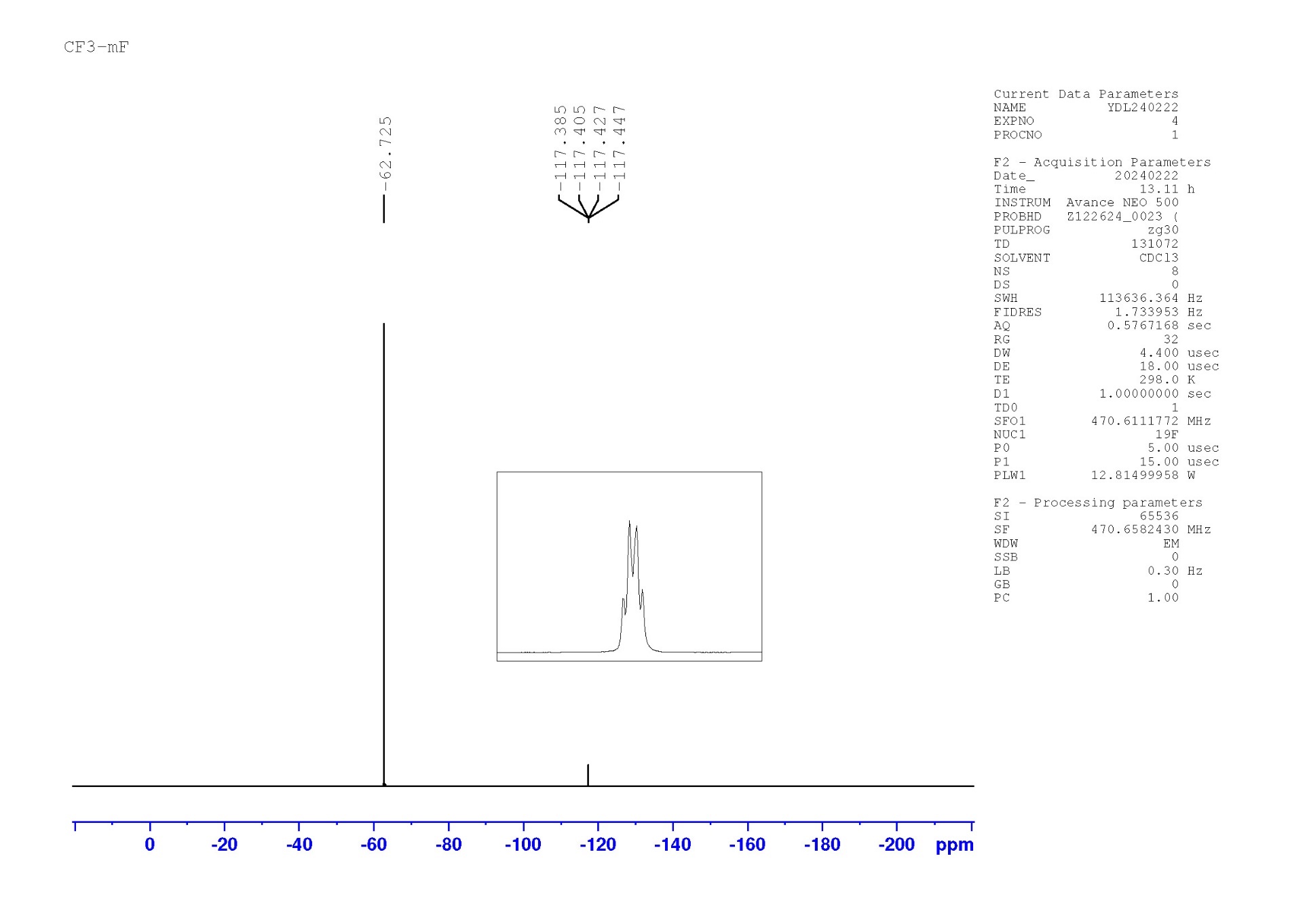


**Figure S34.** ^19^F NMR spectrum of compound **CF3-mF.**


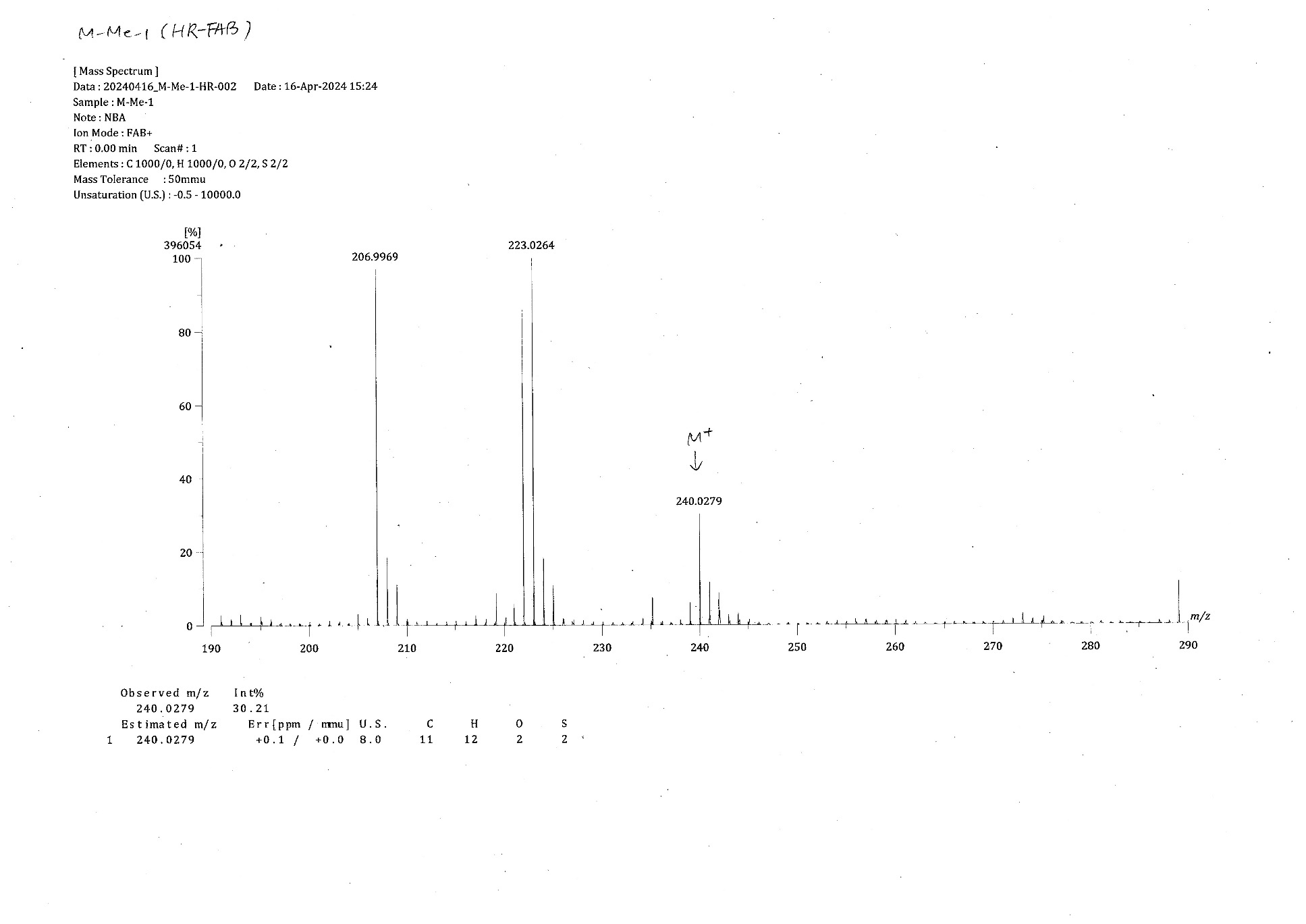


**Figure S35** Mass spectra of **2a**.


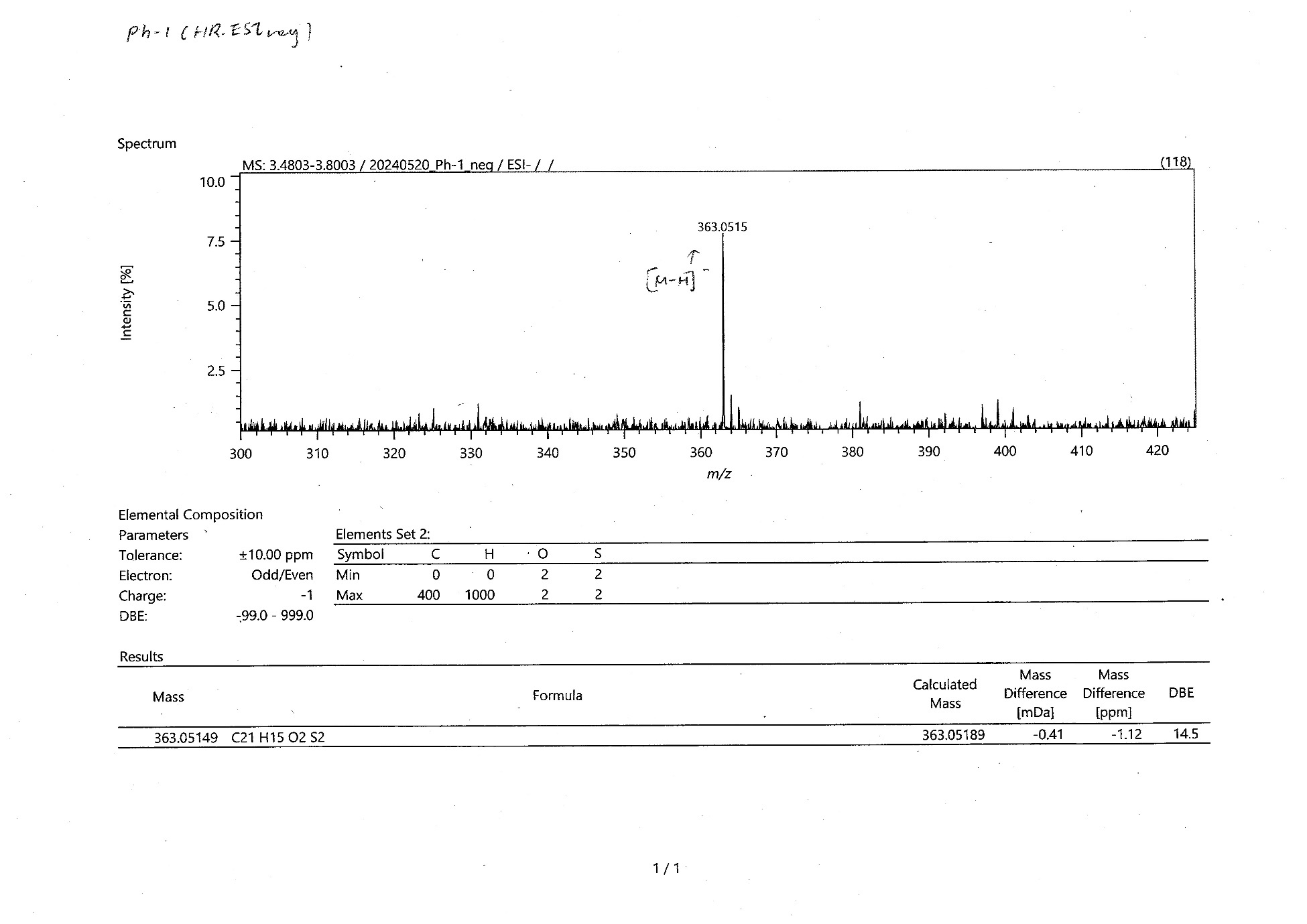


**Figure S36** Mass spectra of **2b**.


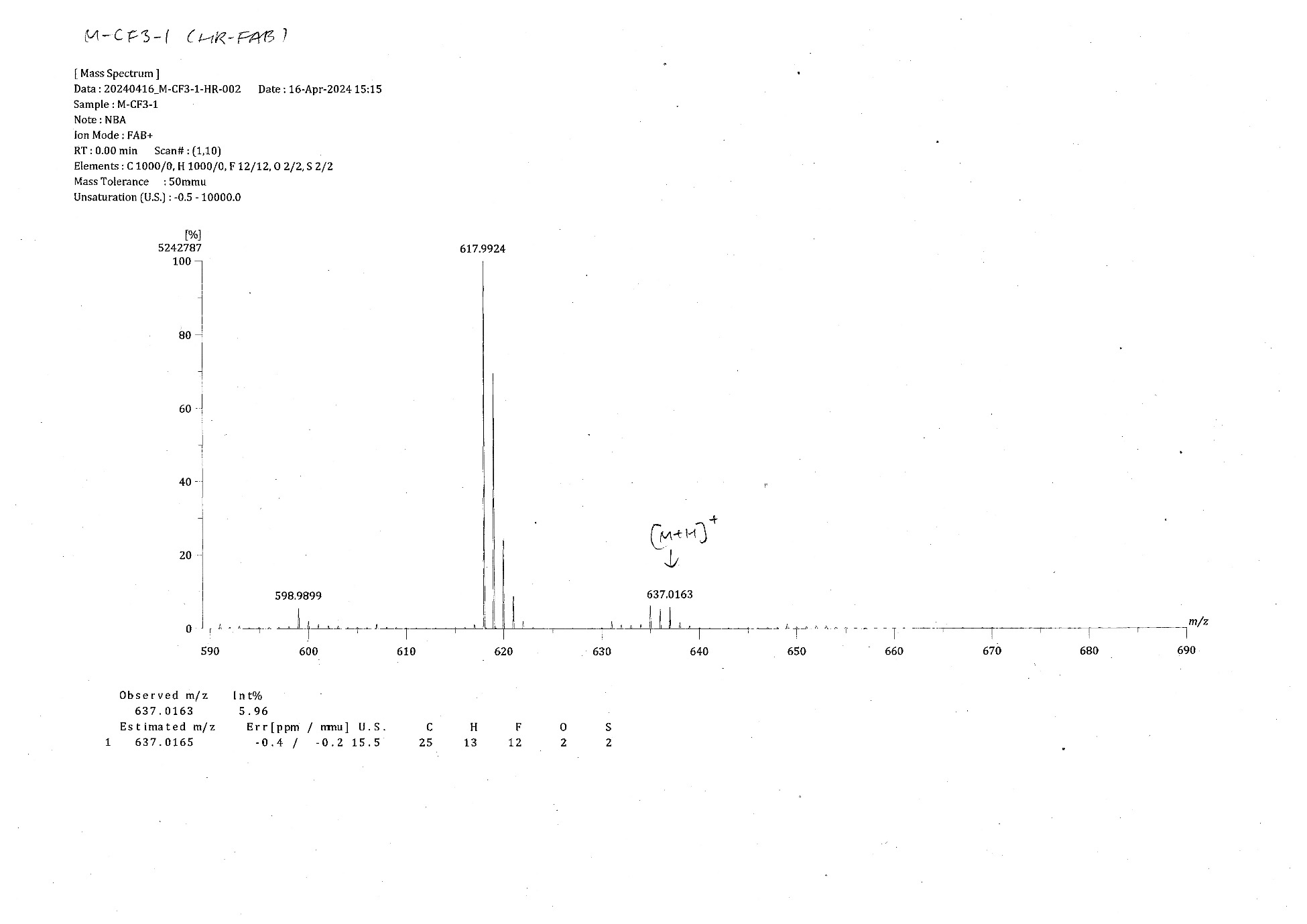


**Figure S37** Mass spectra of **2c**.


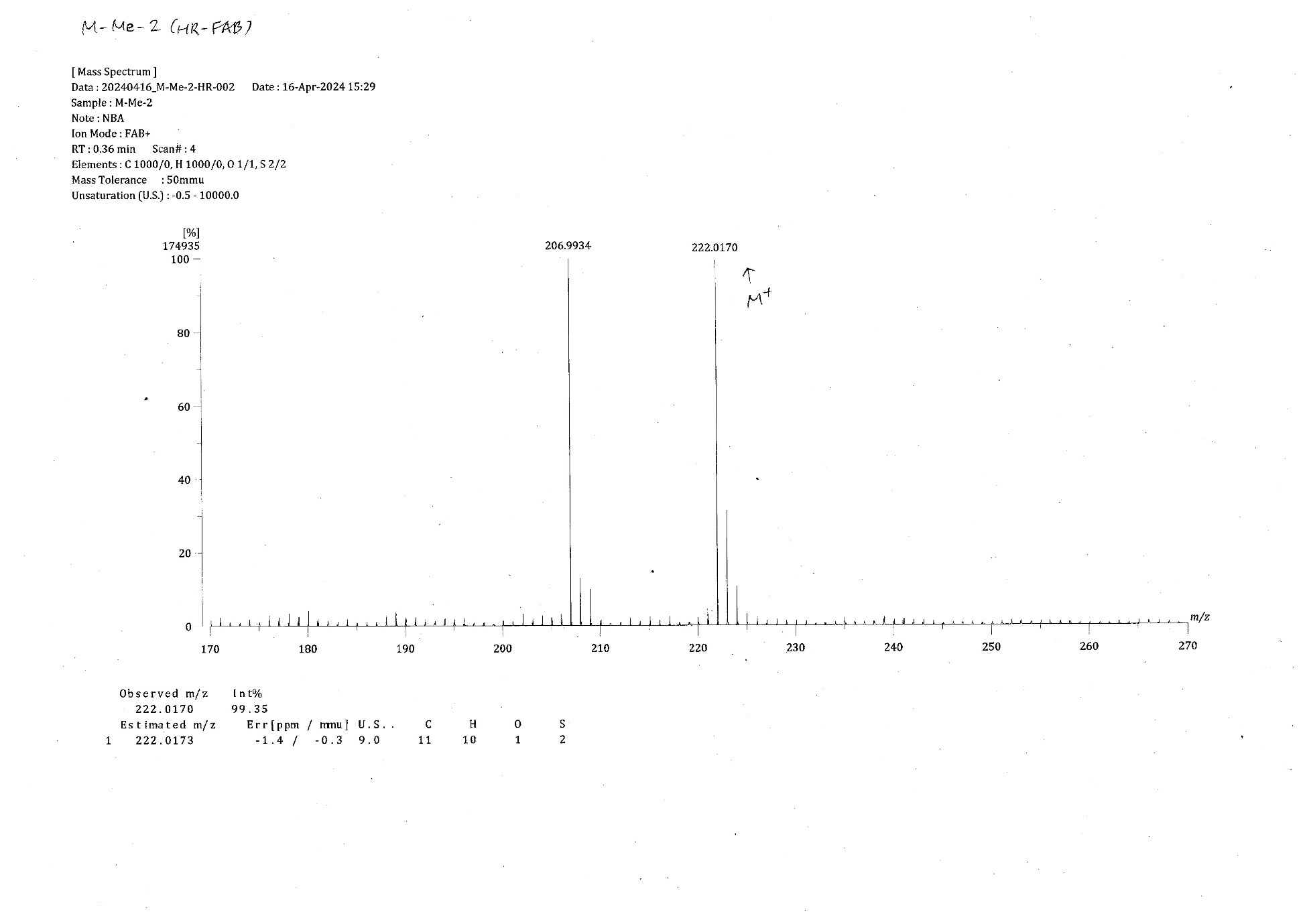


**Figure S38** Mass spectra of **3a**.


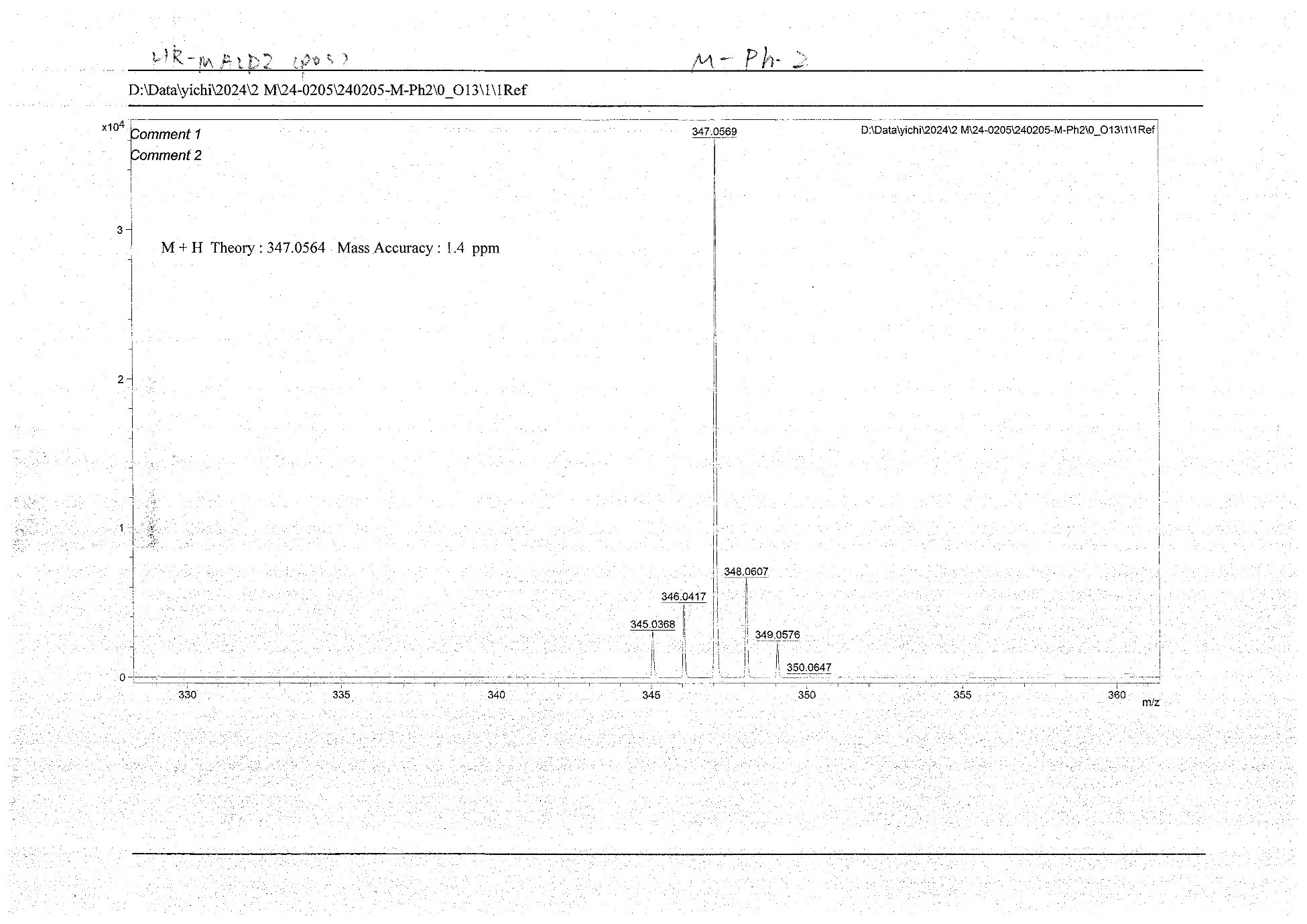


**Figure S39** Mass spectra of **3b**.


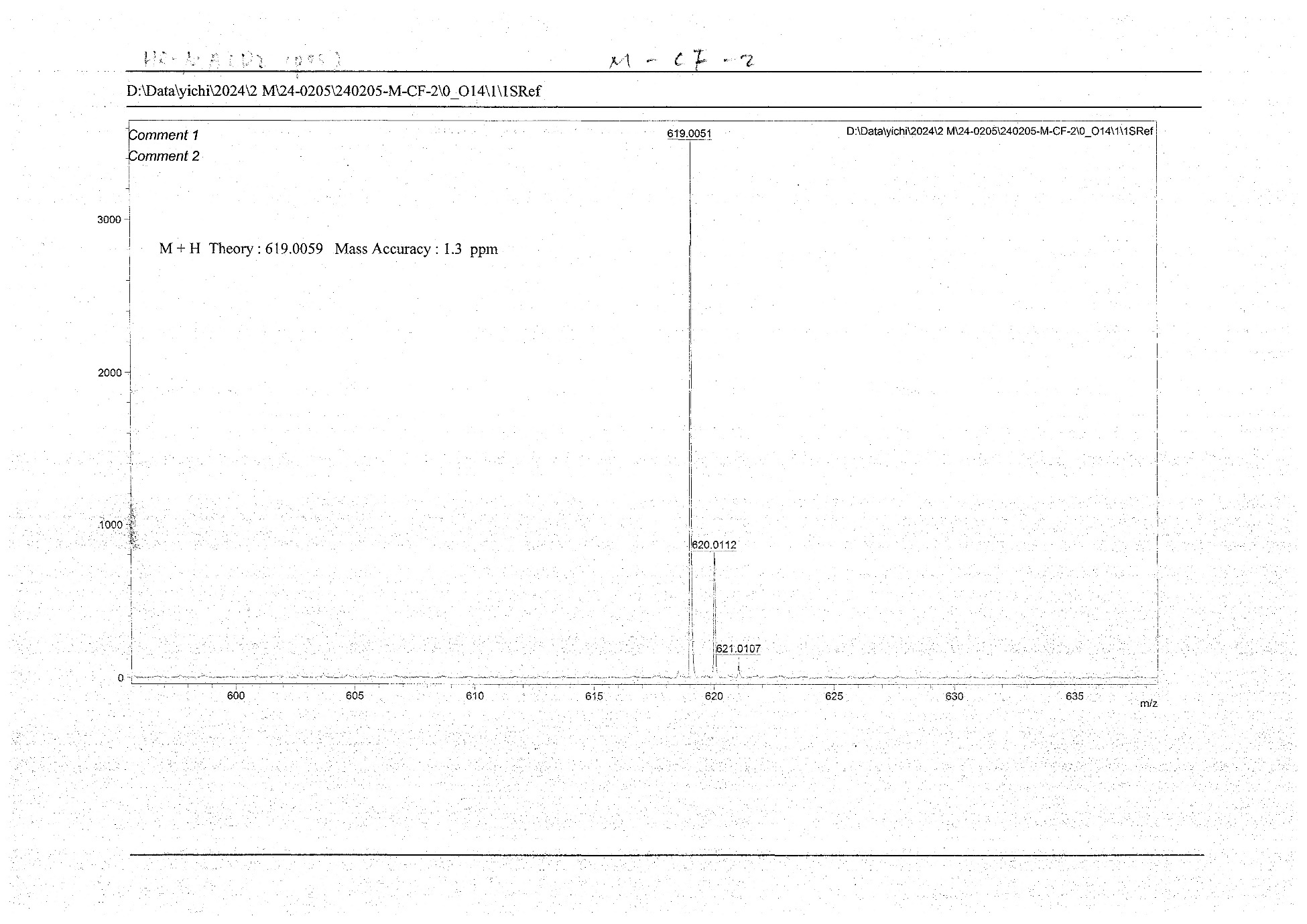


**Figure S40** Mass spectra of **3c**.


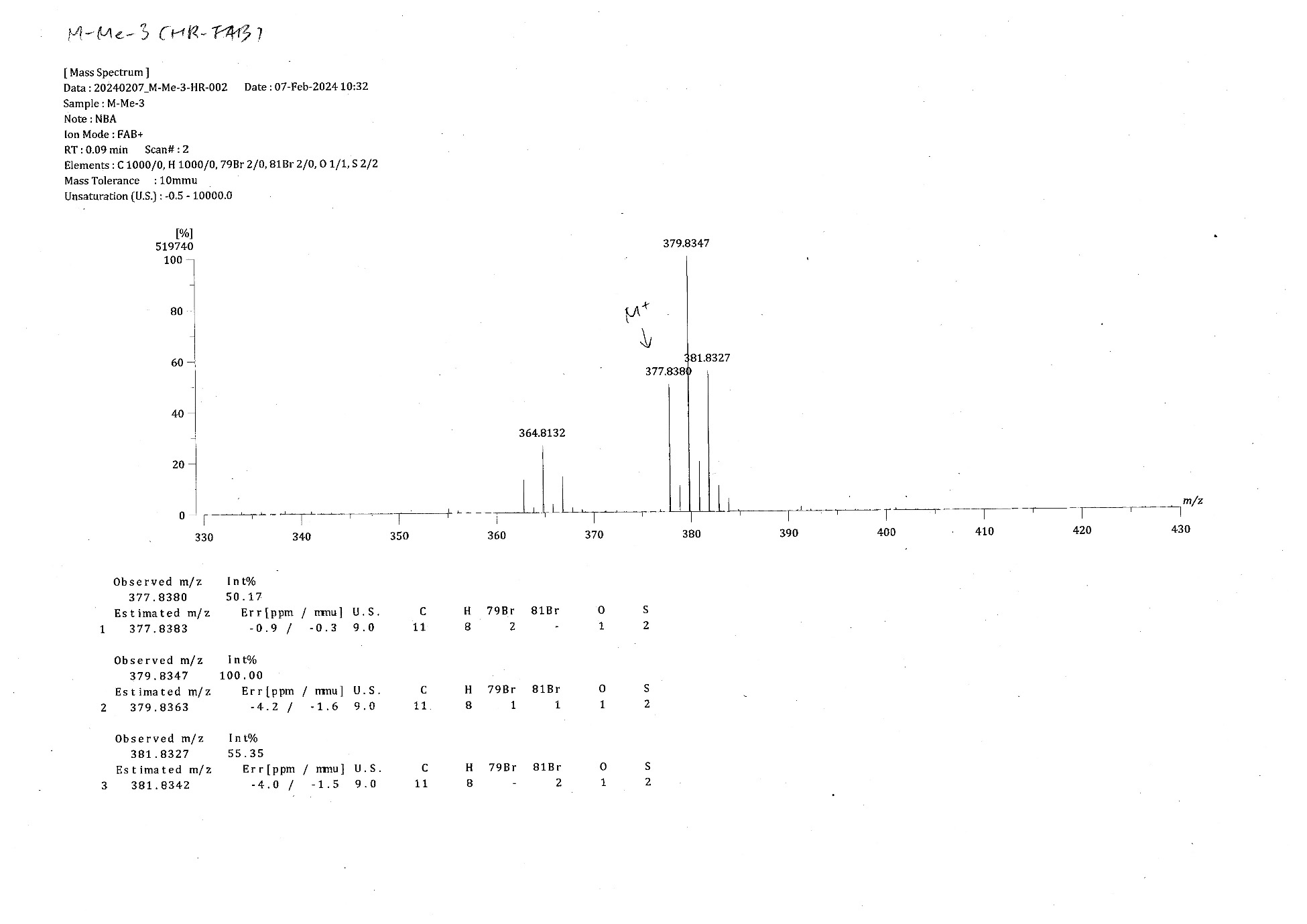


**Figure S41** Mass spectra of **4a**.


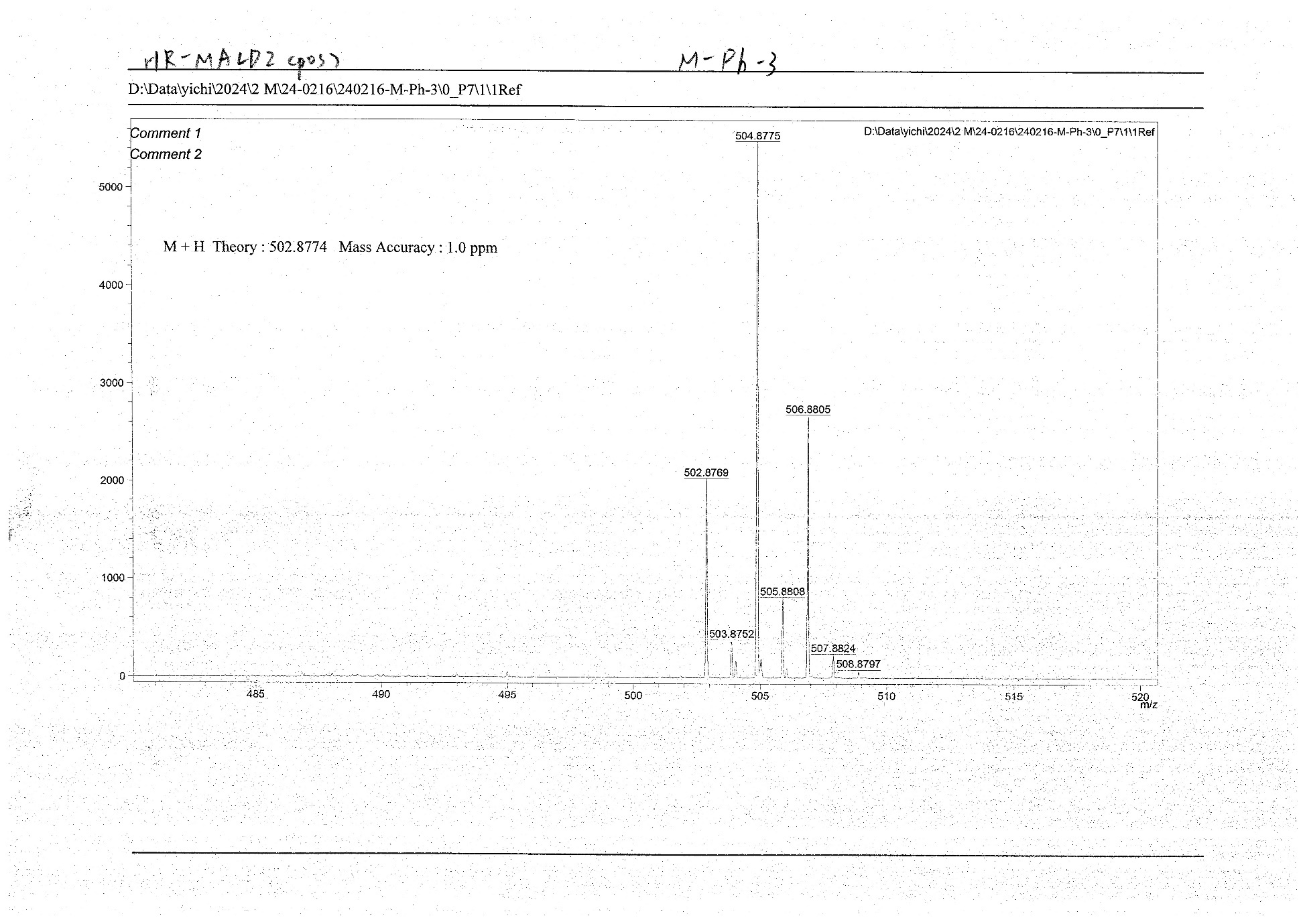


**Figure S42** Mass spectra of **4b**.


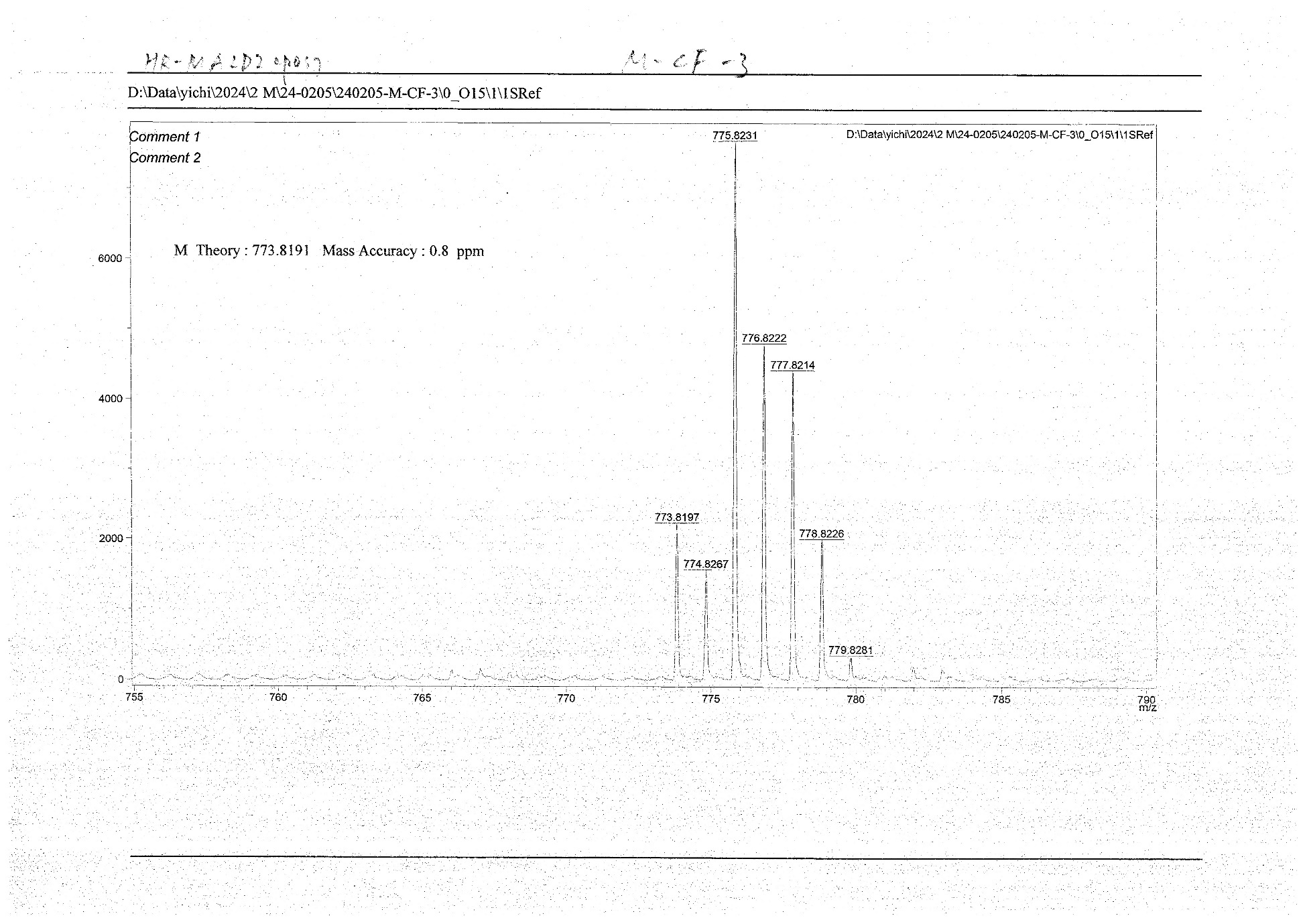


**Figure S43** Mass spectra of **4c**.


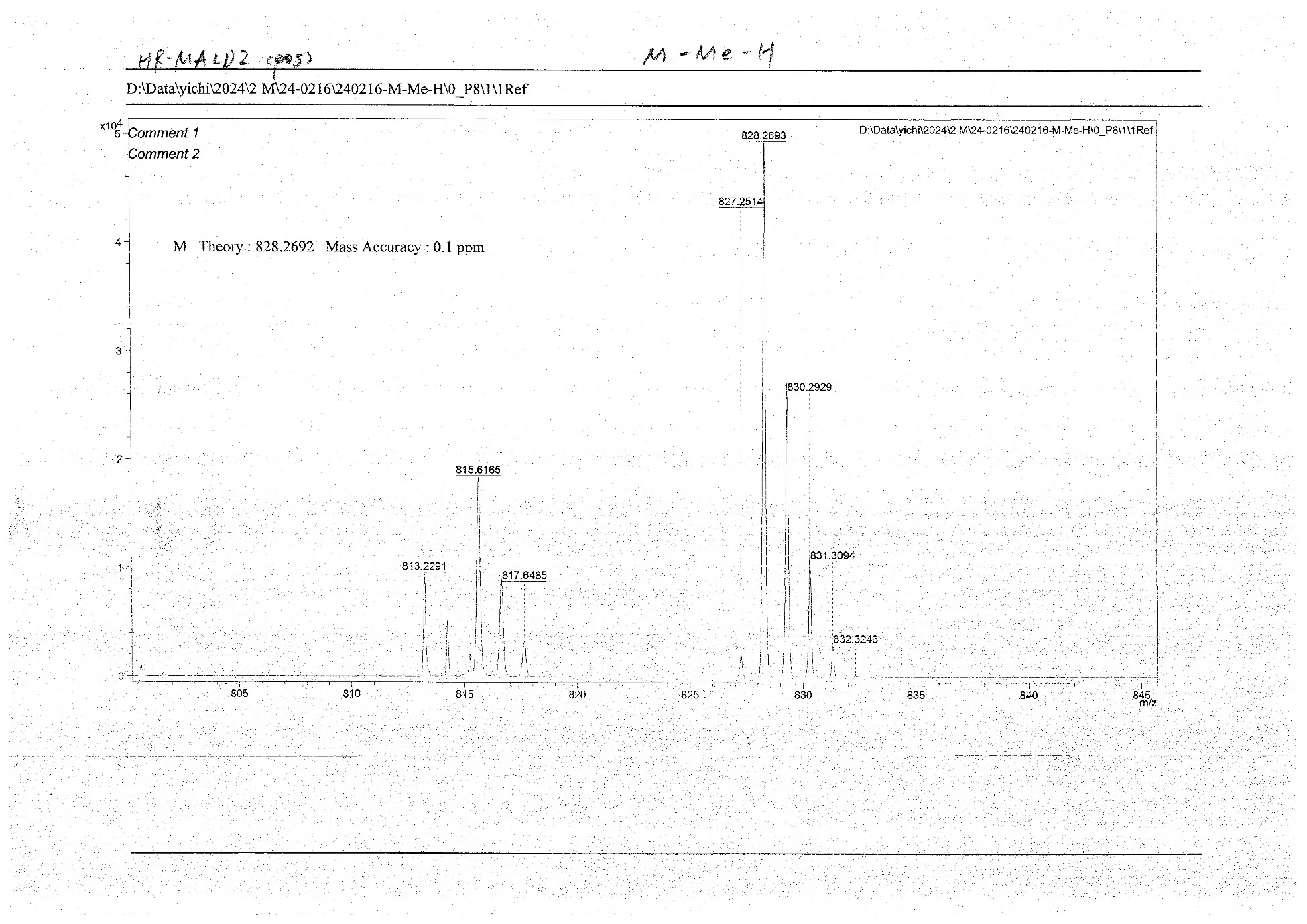


**Figure S44** Mass spectra of **Me-H**.


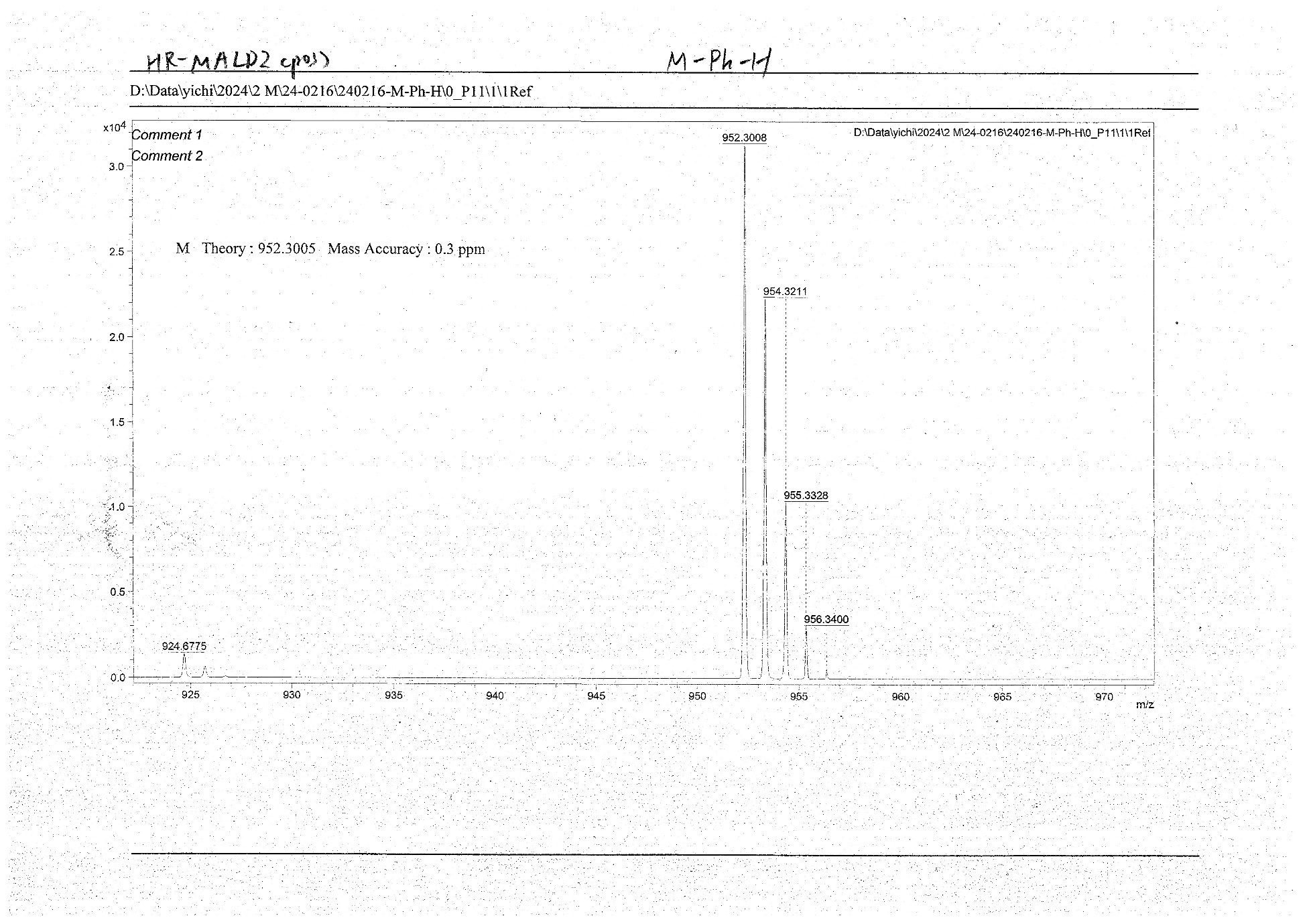


**Figure S45** Mass spectra of **Ph-H**.


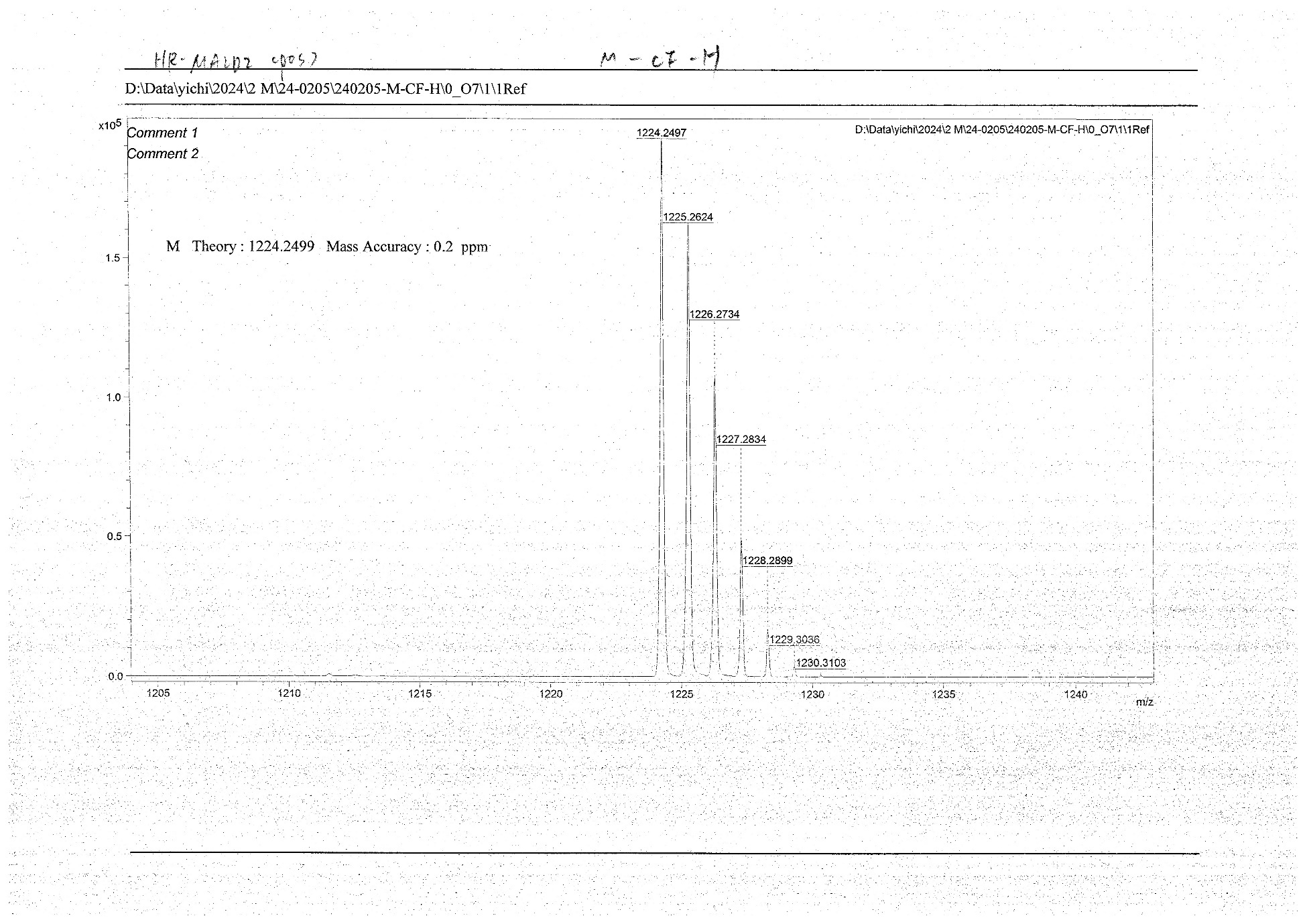


**Figure S46** Mass spectra of **CF3-H**.


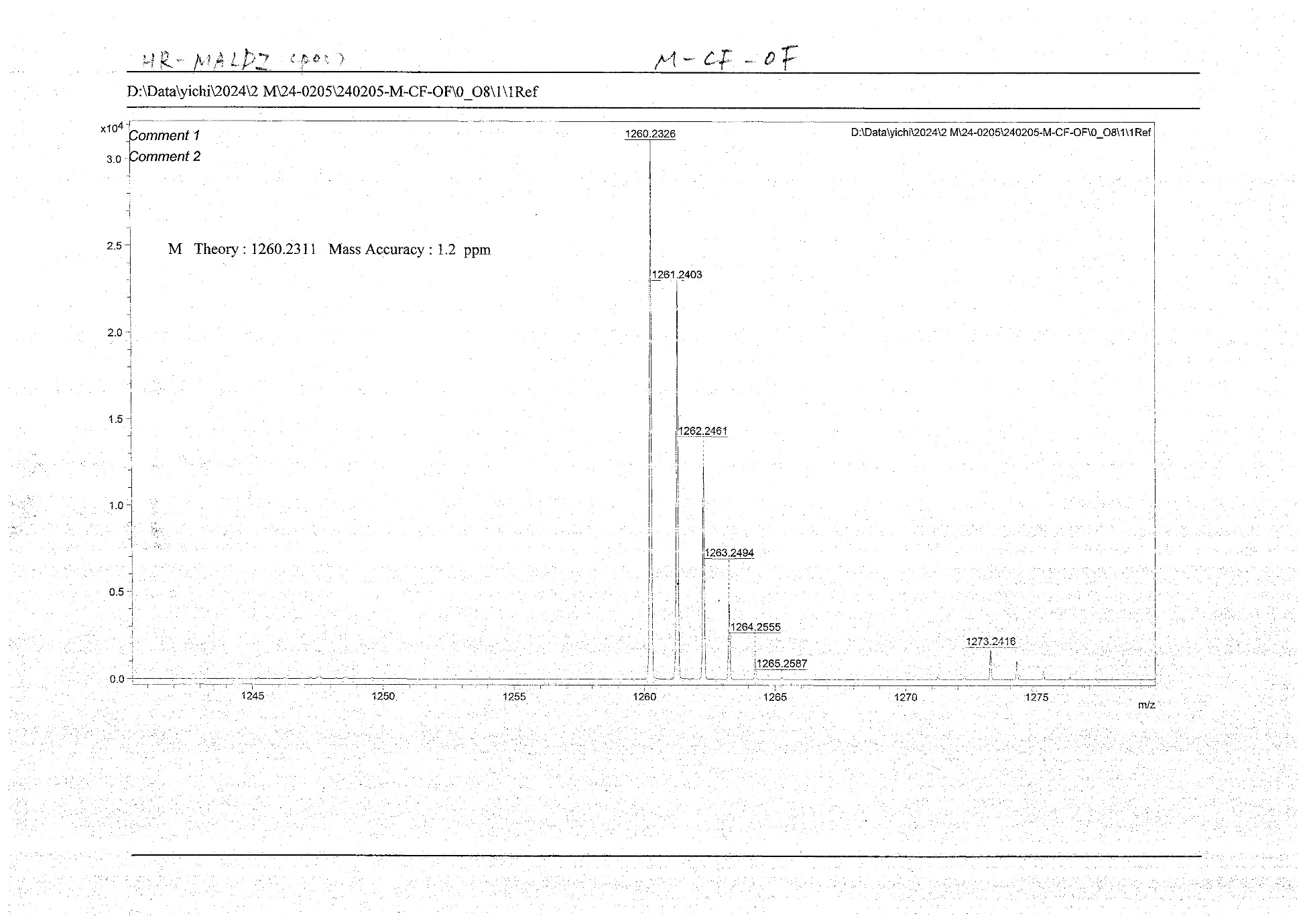


**Figure S47** Mass spectra of **CF3-oF**.


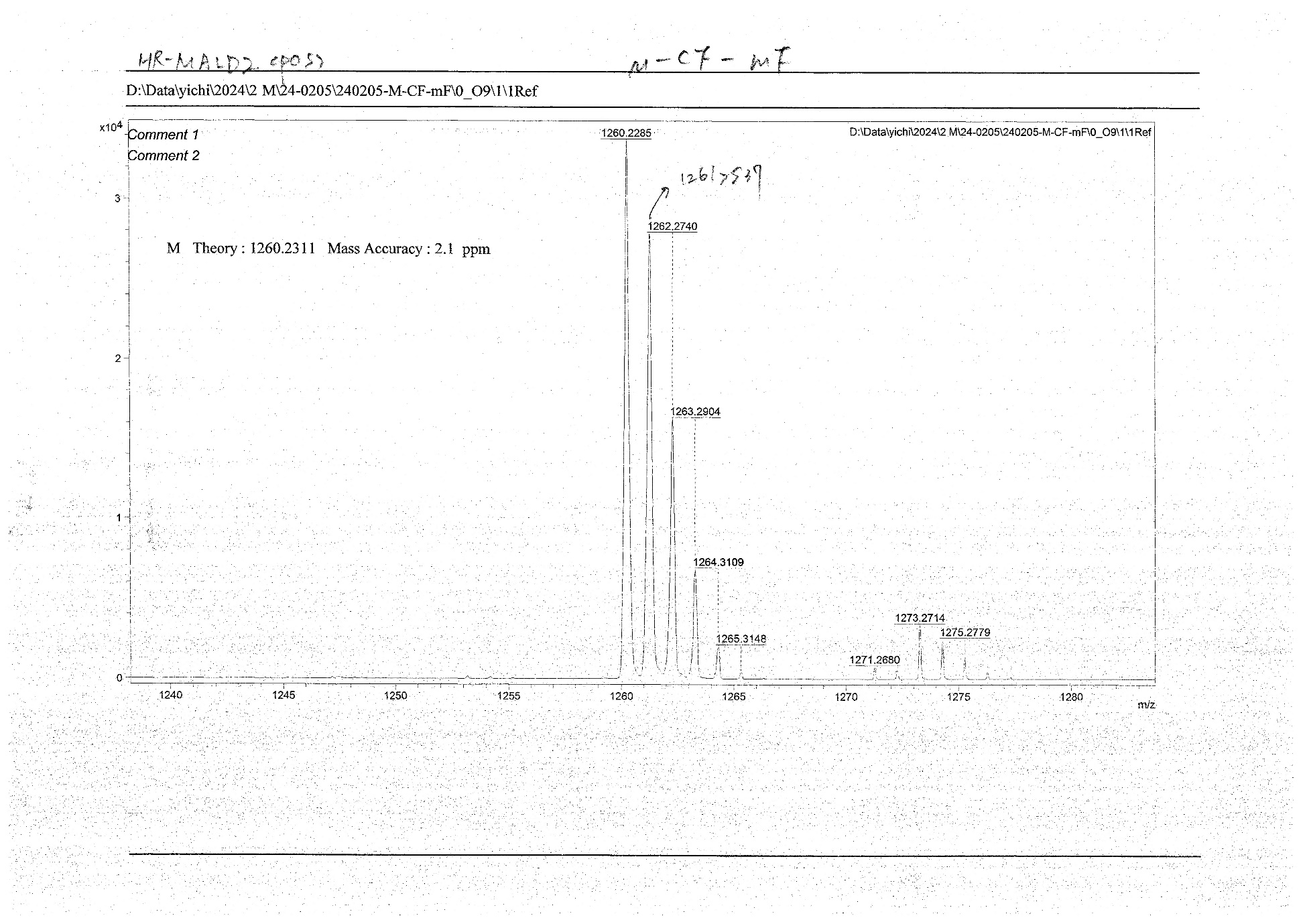


**Figure S48** Mass spectra of **CF3-mF**.

**Figure S49.** (a) TGA curves of the **Me-H**, **Ph-H**, and **CF3-**series. (b-g) DSC curves of the **Me-H**, **Ph-H**, **CF3-**series and spiro-OMeTAD.

**Figure S50.** PXRD powder patterns of (a) **Me-H**, (b) **Ph-H**, (c) **CF3-oF**, (d) **CF3-mF**, and (e) **CF3-H**.


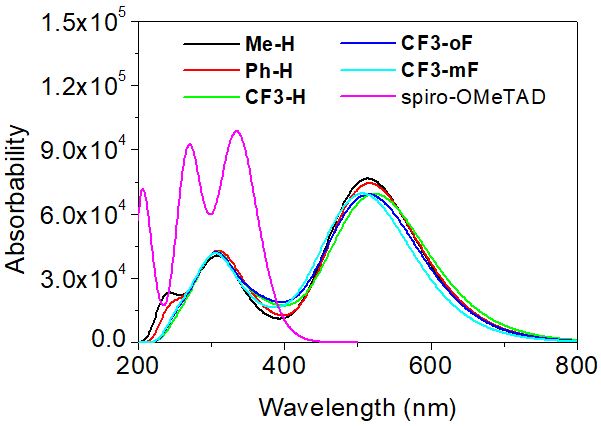


**Figure S51**. Calculated gas-phase absorption spectra of spiro-OMTAD, **Me-H**, **Ph-H**, and **CF3-**series HTMs.


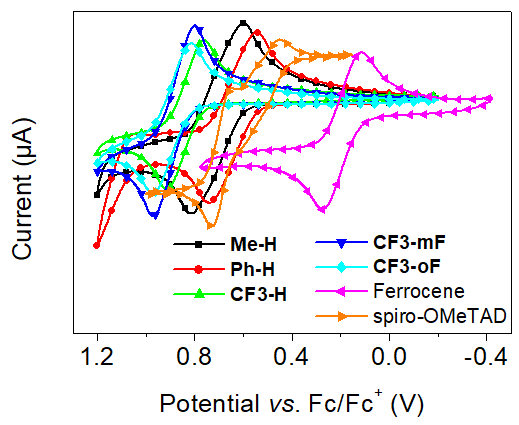


**Figure S52**. Cyclic voltammograms of ferrocene, spiro-OMeTAD, **Me-H**, **Ph-H**, and **CF3-**series with 0.1 M TBAPF_6_ in THF at a scan rate of 100 mV^−1^.

**Figure S53.** Space-charge-limited-current plots in the *J*-*V* characteristics of the devices with spiro-OMTAD, **Me-H**, **Ph-H**, and **CF3-**series as HTM, respectiv

**Figure S54.** XPS signals of Pb 4f, I 3d, and S 2p from a pristine **Me-H** film and a **Me-H** coated perovskite film

**Figure S55.** XPS signals of Pb 4f, I 3d, and S 2p from a pristine **Ph-H** film and a **Ph-H** coated perovskite film

**Figure S56.** XPS signals of Pb 4f, I 3d, F 1s and S 2p from a pristine **CF3-H** film and a **CF3-H** coated perovskite film.

**Figure S57.** XPS signals of Pb 4f, I 3d, F 1s and S 2p from a pristine **CF3-mF** film and a **CF3-mF** coated perovskite film.

**Figure S58.** Plots of *J-V* curves for the perovskite solar cells using spiro-OMTAD, **Me-H**, **Ph-H**, and **CF3-**series as HTM under forward scan (at illumination of 100 mW/cm^2^, AM1.5G).

**Figure S59.** Plots of *J-V* curves for the perovskite solar cells using spiro-OMTAD, **Me-H**, **Ph-H**, and **CF3-**series as HTM without dopants under (a) reverse scan and (b) forward scan (at illumination of 100 mW/cm^2^, AM1.5G).

**Figure S60.** The water contact angles on **Me-H**, **Ph-H**, **CF3-H**, **CF3-mF**, **CF3-oF**, and spiro-OMeTAD films.

**Table S1** Cost Calculation of Compound **1**

| **Reagent** | **Amount(g)** | **Amount(mL)** | **Price($/g or $/mL)** | **Total Price ($)** |
| --- | --- | --- | --- | --- |
| 4H-cyclopenta[2,1-b-3,4-b']dithiophen-4-one | 4 |  | 20 | 80 |
| Sodium perborate monohydrate | 7.8912 |  | 0.4933 | 3.893 |
| Chloroform |  | 35 | 0.0233 | 0.816 |
| Trifluoroacetic acid |  | 35 | 0.76 | 26.6 |
| MgSO_4_ | 50 |  | 0.06 | 3 |
| Water |  | 200 | 0.00 | 0 |
| Ethyl acetate |  | 500 | 0.0033 | 1.65 |
| Hexane |  | 1000 | 0.0011 | 1.1 |
| Silica gel | 120 |  | 0.0042 | 0.504 |
| Total Cost | 117.562 $ | | | |
| Total amount of compound **1** | 1.083 g | | | |
| Cost of compound **1** | 108.552 $/g | | | |

**Table S2** Cost Calculation of Compound **2a**

| **Reagent** | **Amount(g)** | **Amount(mL)** | **Price($/g or $/mL)** | **Total Price ($)** |
| --- | --- | --- | --- | --- |
| compound **1** | 0.5 |  | 108.552 | 54.276 |
| Methylmagnesium bromide, 1M solution in THF |  | 6 | 0.496 | 2.976 |
| THF |  | 12 | 0.034 | 0.408 |
| MgSO_4_ | 3 |  | 0.06 | 0.18 |
| Water |  | 20 | 0.00 | 0 |
| Ethyl acetate |  | 150 | 0.0033 | 0.495 |
| Hexane |  | 1000 | 0.0011 | 1.1 |
| Silica gel | 80 |  | 0.0042 | 0.336 |
| Total Cost | 59.771 $ | | | |
| Total amount of compound **2a** | 0.5366 g | | | |
| Cost of compound **2a** | 111.389 $/g | | | |

**Table S3** Cost Calculation of Compound **3a**

| **Reagent** | **Amount(g)** | **Amount(mL)** | **Price($/g or $/mL)** | **Total Price ($)** |
| --- | --- | --- | --- | --- |
| compound **2a** | 0.5 |  | 111.389 | 55.694 |
| *p*-Toluenesulfonic acid | 0.035 |  | 0.96 | 0.0336 |
| Toluene |  | 15 | 0.004 | 0.06 |
| MgSO_4_ | 3 |  | 0.06 | 0.18 |
| Water |  | 15 | 0.00 | 0 |
| Ethyl acetate |  | 60 | 0.0033 | 0.198 |
| Hexane |  | 600 | 0.0011 | 0.66 |
| Silica gel | 60 |  | 0.0042 | 0.252 |
| Total Cost | 57.078 $ | | | |
| Total amount of compound **3a** | 0.4348 g | | | |
| Cost of compound **3a** | 131.274 $/g | | | |

**Table S4** Cost Calculation of Compound **4a**

| **Reagent** | **Amount(g)** | **Amount(mL)** | **Price($/g or $/mL)** | **Total Price ($)** |
| --- | --- | --- | --- | --- |
| compound **3a** | 0.5 |  | 131.274 | 65.637 |
| NBS | 0.84 |  | 0.094 | 0.07896 |
| Dichloromethane |  | 40 | 0.0046 | 0.184 |
| MgSO_4_ | 3 |  | 0.06 | 0.18 |
| Water |  | 15 | 0.00 | 0 |
| Hexane |  | 600 | 0.0011 | 0.66 |
| Silica gel | 50 |  | 0.0042 | 0.21 |
| Total Cost | 66.950 $ | | | |
| Total amount of compound **4a** | 0.8549 g | | | |
| Cost of compound **4a** | 78.313 $/g | | | |

**Table S5** Cost Calculation of Compound **Me-H**

| **Reagent** | **Amount(g)** | **Amount(mL)** | **Price($/g or $/mL)** | **Total Price ($)** |
| --- | --- | --- | --- | --- |
| compound **4a** | 0.5 |  | 78.313 | 39.156 |
| compound **5** | 1.418 |  | 11.47 | 16.264 |
| Pd(PPh_3_)_4_ | 0.105 |  | 22.24 | 2.335 |
| K_2_CO_3_ | 1.9088 |  | 0.03 | 0.057 |
| Water |  | 40 | 0.00 | 0 |
| THF |  | 13.15 | 0.034 | 0.447 |
| MgSO_4_ | 15 |  | 0.06 | 0.9 |
| Ethyl acetate |  | 160 | 0.0033 | 0.528 |
| Hexane |  | 1000 | 0.0011 | 1.1 |
| Silica gel | 80 |  | 0.0042 | 0.336 |
| Total Cost | 61.125 $ | | | |
| Total amount of compound **Me-H** | 0.8833 g | | | |
| Cost of compound **Me-H** | 69.200 $/g | | | |

**Table S6** Cost Calculation of Compound **2b**

| **Reagent** | **Amount(g)** | **Amount(mL)** | **Price($/g or $/mL)** | **Total Price ($)** |
| --- | --- | --- | --- | --- |
| compound **1** | 0.5 |  | 108.552 | 54.276 |
| Phenylmagnesium bromide solution 1.0 M in THF |  | 6 | 1.3667 | 8.200 |
| THF |  | 12 | 0.034 | 0.408 |
| MgSO_4_ | 3 |  | 0.06 | 0.18 |
| Water |  | 20 | 0.00 | 0 |
| Ethyl acetate |  | 130 | 0.0033 | 0.429 |
| Hexane |  | 1000 | 0.0011 | 1.1 |
| Silica gel | 80 |  | 0.0042 | 0.336 |
| Total Cost | 64.929 $ | | | |
| Total amount of compound **2b** | 0.8225 g | | | |
| Cost of compound **2b** | 78.942 $/g | | | |

**Table S7** Cost Calculation of Compound **3b**

| **Reagent** | **Amount(g)** | **Amount(mL)** | **Price($/g or $/mL)** | **Total Price ($)** |
| --- | --- | --- | --- | --- |
| compound **2b** | 0.5 |  | 78.942 | 39.471 |
| *p*-Toluenesulfonic acid | 0.024 |  | 0.96 | 0.023 |
| Toluene |  | 15 | 0.004 | 0.06 |
| MgSO4 | 3 |  | 0.06 | 0.18 |
| Water |  | 15 | 0.00 | 0 |
| Ethyl acetate |  | 60 | 0.0033 | 0.198 |
| Hexane |  | 600 | 0.0011 | 0.66 |
| Silica gel | 60 |  | 0.0042 | 0.252 |
| Total Cost | 40.844 $ | | | |
| Total amount of compound **3b** | 0.4563 g | | | |
| Cost of compound **3b** | 89.511 $/g | | | |

**Table S8** Cost Calculation of Compound **4b**

| **Reagent** | **Amount(g)** | **Amount(mL)** | **Price($/g or $/mL)** | **Total Price ($)** |
| --- | --- | --- | --- | --- |
| compound **3b** | 0.5 |  | 89.511 | 44.755 |
| NBS | 0.54 |  | 0.094 | 0.051 |
| Dichloromethane |  | 40 | 0.0046 | 0.184 |
| MgSO_4_ | 3 |  | 0.06 | 0.18 |
| Water |  | 15 | 0.00 | 0 |
| Hexane |  | 600 | 0.0011 | 0.66 |
| Silica gel | 50 |  | 0.0042 | 0.21 |
| Total Cost | 46.040 $ | | | |
| Total amount of compound **4b** | 0.6549 g | | | |
| Cost of compound **4b** | 70.301 $/g | | | |

**Table S9** Cost Calculation of Compound **Ph-H**

| **Reagent** | **Amount(g)** | **Amount(mL)** | **Price($/g or $/mL)** | **Total Price ($)** |
| --- | --- | --- | --- | --- |
| compound **4b** | 0.5 |  | 70.301 | 35.151 |
| compound **5** | 1.07 |  | 11.47 | 12.273 |
| Pd(PPh_3_)_4_ | 0.092 |  | 22.24 | 2.046 |
| K_2_CO_3_ | 1.4389 |  | 0.03 | 0.043 |
| Water |  | 40 | 0.00 | 0 |
| THF |  | 9.9 | 0.034 | 0.337 |
| MgSO_4_ | 15 |  | 0.06 | 0.9 |
| Ethyl acetate |  | 160 | 0.0033 | 0.528 |
| Hexane |  | 1000 | 0.0011 | 1.1 |
| Silica gel | 80 |  | 0.0042 | 0.336 |
| Total Cost | 52.713 $ | | | |
| Total amount of compound **Ph-H** | 0.8033 g | | | |
| Cost of compound **Ph-H** | 65.621 $/g | | | |

**Table S10** Cost Calculation of Compound **2c**

| **Reagent** | **Amount(g)** | **Amount(mL)** | **Price($/g or $/mL)** | **Total Price ($)** |
| --- | --- | --- | --- | --- |
| compound **1** | 0.5 |  | 108.552 | 54.276 |
| 0.5 M 3,5-Bis(trifluoromethyl)phenylmagnesium bromide solution in THF |  | 12 | 2.767 | 33.200 |
| THF |  | 12 | 0.034 | 0.408 |
| MgSO_4_ | 3 |  | 0.06 | 0.18 |
| Water |  | 20 | 0.00 | 0 |
| Ethyl acetate |  | 110 | 0.0033 | 0.363 |
| Hexane |  | 1000 | 0.0011 | 1.1 |
| Silica gel | 80 |  | 0.0042 | 0.336 |
| Total Cost | 89.864 $ | | | |
| Total amount of compound **2c** | 1.4975 g | | | |
| Cost of compound **2c** | 60.009 $/g | | | |

**Table S11** Cost Calculation of Compound **3c**

| **Reagent** | **Amount(g)** | **Amount(mL)** | **Price($/g or $/mL)** | **Total Price ($)** |
| --- | --- | --- | --- | --- |
| compound **2c** | 0.5 |  | 60.009 | 30.005 |
| *p*-Toluenesulfonic acid | 0.0014 |  | 0.96 | 0.001 |
| Toluene |  | 15 | 0.004 | 0.06 |
| MgSO_4_ | 3 |  | 0.06 | 0.18 |
| Water |  | 15 | 0.00 | 0 |
| Ethyl acetate |  | 60 | 0.0033 | 0.198 |
| Hexane |  | 600 | 0.0011 | 0.66 |
| Silica gel | 60 |  | 0.0042 | 0.252 |
| Total Cost | 31.356 $ | | | |
| Total amount of compound **3c** | 0.4712 g | | | |
| Cost of compound **3c** | 66.545 $/g | | | |

**Table S12** Cost Calculation of Compound **4c**

| **Reagent** | **Amount(g)** | **Amount(mL)** | **Price($/g or $/mL)** | **Total Price ($)** |
| --- | --- | --- | --- | --- |
| compound **3c** | 0.5 |  | 66.545 | 33.272 |
| NBS | 0.3 |  | 0.094 | 0.0282 |
| Dichloromethane |  | 40 | 0.0046 | 0.184 |
| MgSO_4_ | 3 |  | 0.06 | 0.18 |
| Water |  | 15 | 0.00 | 0 |
| Hexane |  | 600 | 0.0011 | 0.66 |
| Silica gel | 50 |  | 0.0042 | 0.21 |
| Total Cost | 34.535 $ | | | |
| Total amount of compound **4c** | 0.59 g | | | |
| Cost of compound **4c** | 58.533 $/g | | | |

**Table S13** Cost Calculation of Compound **CF3-H**

| **Reagent** | **Amount(g)** | **Amount(mL)** | **Price($/g or $/mL)** | **Total Price ($)** |
| --- | --- | --- | --- | --- |
| compound **4c** | 0.5 |  | 58.533 | 29.267 |
| compound **5** | 0.695 |  | 11.47 | 7.972 |
| Pd(PPh_3_)_4_ | 0.06 |  | 22.24 | 1.334 |
| K_2_CO_3_ | 0.9347 |  | 0.03 | 0.028 |
| Water |  | 30 | 0.00 | 0 |
| THF |  | 6.45 | 0.034 | 0.219 |
| MgSO_4_ | 15 |  | 0.06 | 0.9 |
| Ethyl acetate |  | 120 | 0.0033 | 0.396 |
| Hexane |  | 1000 | 0.0011 | 1.1 |
| Silica gel | 80 |  | 0.0042 | 0.336 |
| Total Cost | 41.552 $ | | | |
| Total amount of compound **CF3-H** | 0.8833 g | | | |
| Cost of compound **CF3-H** | 47.042 $/g | | | |

**Table S14** Cost Calculation of Compound **CF3-oF**

| **Reagent** | **Amount(g)** | **Amount(mL)** | **Price($/g or $/mL)** | **Total Price ($)** |
| --- | --- | --- | --- | --- |
| compound **4c** | 0.5 |  | 58.533 | 29.267 |
| compound **6** | 0.724 |  | 36.57 | 26.47668 |
| Pd(PPh_3_)_4_ | 0.105 |  | 22.24 | 2.3352 |
| K_2_CO_3_ | 0.9347 |  | 0.03 | 0.028041 |
| Water |  | 40 | 0.00 | 0 |
| THF |  | 13.15 | 0.034 | 0.4471 |
| MgSO_4_ | 15 |  | 0.06 | 0.9 |
| Ethyl acetate |  | 100 | 0.0033 | 0.33 |
| Hexane |  | 1000 | 0.0011 | 1.1 |
| Silica gel | 80 |  | 0.0042 | 0.336 |
| Total Cost | 61.220 $ | | | |
| Total amount of compound **CF3-oF** | 0.8306 g | | | |
| Cost of compound **CF3-oF** | 73.705 $/g | | | |

**Table S15** Cost Calculation of Compound **CF3-mF**

| **Reagent** | **Amount(g)** | **Amount(mL)** | **Price($/g or $/mL)** | **Total Price ($)** |
| --- | --- | --- | --- | --- |
| compound **4c** | 0.5 |  | 58.533 | 29.267 |
| compound **7** | 0.724 |  | 35.19 | 25.478 |
| Pd(PPh_3_)_4_ | 0.105 |  | 22.24 | 2.335 |
| K_2_CO_3_ | 0.9347 |  | 0.03 | 0.028 |
| Water |  | 40 | 0.00 | 0 |
| THF |  | 13.15 | 0.034 | 0.447 |
| MgSO_4_ | 15 |  | 0.06 | 0.9 |
| Ethyl acetate |  | 100 | 0.0033 | 0.33 |
| Hexane |  | 1000 | 0.0011 | 1.1 |
| Silica gel | 80 |  | 0.0042 | 0.336 |
| Total Cost | 60.220 $ | | | |
| Total amount of compound **CF3-mF** | 0.8079 g | | | |
| Cost of compound **CF3-mF** | 74.540 $/g | | | |

**Table S16** Calculated TDDFT excitation energies (*E*), oscillator strengths (*f*), MO compositions and characters for **Me-H**.

| **HTM** | **n** | **E(eV, nm)** | **f** | **Composition** | **Character** |
| --- | --- | --- | --- | --- | --- |
| **Me-H** | 1 | 2.4095(514.56) | 1.8736 | HOMO → LUMO | CT |
|  | 2 | 2.9001(427.51) | 0.0657 | HOMO-1 → LUMO | π→π* |
|  | 3 | 3.3225(373.16) | 0.0322 | HOMO-2 → LUMO | π→π* |
|  | 4 | 3.3828(366.51) | 0.1225 | HOMO → LUMO+1 | π→π* |
|  | 5 | 3.4509(359.28) | 0.0443 | 4% HOMO-1 → LUMO+2 | π→π* |
|  |  |  |  | 9% HOMO-1 → LUMO+3 | π→π* |
|  |  |  |  | 3% HOMO → LUMO+1 | π→π* |
|  |  |  |  | 55% HOMO → LUMO+2 | π→π* |
|  |  |  |  | 29% HOMO → LUMO+3 | π→π* |
|  | 6 | 3.4628(358.05) | 0.0068 | 11% HOMO-1 → LUMO+2 | π→π* |
|  |  |  |  | 4% HOMO-1 → LUMO+3 | π→π* |
|  |  |  |  | 28% HOMO → LUMO+2 | π→π* |
|  |  |  |  | 56% HOMO → LUMO+3 | π→π* |
|  | 7 | 3.7628(329.50) | 0.0251 | 6% HOMO-1 → LUMO+3 | π→π* |
|  |  |  |  | 34% HOMO → LUMO+4 | π→π* |
|  |  |  |  | 12% HOMO → LUMO+5 | π→π* |
|  |  |  |  | 2% HOMO → LUMO+6 | π→π* |
|  |  |  |  | 33% HOMO → LUMO+7 | π→π* |
|  |  |  |  | 3% HOMO → LUMO+9 | π→π* |
|  |  |  |  | 10% HOMO → LUMO+11 | π→π* |
|  | 8 | 3.8206(324.52) | 0.2735 | HOMO-1 → LUMO+1 | π→π* |
|  | 9 | 3.9052(317.49) | 0.1138 | 5% HOMO-1 → LUMO+2 | π→π* |
|  |  |  |  | 13% HOMO-1 → LUMO+4 | π→π* |
|  |  |  |  | 48% HOMO → LUMO+4 | π→π* |
|  |  |  |  | 22% HOMO → LUMO+5 | π→π* |
|  |  |  |  | 9% HOMO → LUMO+7 | π→π* |
|  |  |  |  | 3% HOMO → LUMO+10 | π→π* |
|  | 10 | 3.9279(315.65) | 0.0026 | 7% HOMO-2 → LUMO+2 | π→π* |
|  |  |  |  | 67% HOMO-1 → LUMO+2 | π→π* |
|  |  |  |  | 13% HOMO → LUMO+2 | π→π* |
|  |  |  |  | 2% HOMO → LUMO+3 | π→π* |
|  |  |  |  | 6% HOMO → LUMO+5 | π→π* |
|  |  |  |  | 5% HOMO → LUMO+9 | π→π* |
|  | 11 | 3.9561(313.40) | 0.1620 | 2% HOMO-2 → LUMO+3 | π→π* |
|  |  |  |  | 17% HOMO-1 → LUMO+3 | π→π* |
|  |  |  |  | 10% HOMO-1 → LUMO+5 | π→π* |
|  |  |  |  | 2% HOMO-1 → LUMO+7 | π→π* |
|  |  |  |  | 3% HOMO → LUMO+3 | π→π* |
|  |  |  |  | 7% HOMO → LUMO+4 | π→π* |
|  |  |  |  | 38% HOMO → LUMO+5 | π→π* |
|  |  |  |  | 19% HOMO → LUMO+7 | π→π* |
|  | 12 | 3.9638(312.79) | 0.0544 | 4% HOMO-2 → LUMO+3 | π→π* |
|  |  |  |  | 35% HOMO-1 → LUMO+3 | π→π* |
|  |  |  |  | 5% HOMO-1 → LUMO+6 | π→π* |
|  |  |  |  | 6% HOMO → LUMO+3 | π→π* |
|  |  |  |  | 6% HOMO → LUMO+5 | π→π* |
|  |  |  |  | 45% HOMO → LUMO+6 | π→π* |

**Table S17** Calculated TDDFT excitation energies (*E*), oscillator strengths (*f*), MO compositions and characters for **Ph-H**.

| **HTM** | **n** | **E(eV, nm)** | **f** | **Composition** | **Character** |
| --- | --- | --- | --- | --- | --- |
| **Ph-H** | 1 | 2.3961(517.45) | 1.8118 | HOMO → LUMO | CT |
|  | 2 | 2.8509(434.89) | 0.0829 | HOMO-1 → LUMO | π→π* |
|  | 3 | 3.3235(373.06) | 0.0561 | HOMO-2 → LUMO | π→π* |
|  | 4 | 3.3898(365.76) | 0.1425 | HOMO → LUMO+1 | π→π* |
|  | 5 | 3.4742(356.87) | 0.0453 | 12% HOMO-1 → LUMO+3 | π→π* |
|  |  |  |  | 3% HOMO → LUMO+1 | π→π* |
|  |  |  |  | 46% HOMO → LUMO+2 | π→π* |
|  |  |  |  | 39% HOMO → LUMO+3 | π→π* |
|  | 6 | 3.4880(355.45) | 0.0104 | 16% HOMO-1 → LUMO+2 | π→π* |
|  |  |  |  | 37% HOMO → LUMO+2 | π→π* |
|  |  |  |  | 47% HOMO → LUMO+3 | π→π* |
|  | 7 | 3.6200(342.49) | 0.0370 | 3% HOMO-2 → LUMO+4 | π→π* |
|  |  |  |  | 3% HOMO-1 → LUMO+3 | π→π* |
|  |  |  |  | 94% HOMO → LUMO+4 | π→π* |
|  | 8 | 3.7727(328.63) | 0.0123 | 3% HOMO-2 → LUMO+5 | π→π* |
|  |  |  |  | 88% HOMO → LUMO+5 | π→π* |
|  |  |  |  | 9% HOMO → LUMO+6 | π→π* |
|  | 9 | 3.7838(327.67) | 0.2483 | 51% HOMO-1 → LUMO+1 | π→π* |
|  |  |  |  | 9% HOMO → LUMO+5 | π→π* |
|  |  |  |  | 38% HOMO → LUMO+6 | π→π* |
|  |  |  |  | 2% HOMO → LUMO+11 | π→π* |
|  | 10 | 3.8193(324.63) | 0.0591 | 54% HOMO-1 → LUMO+1 | π→π* |
|  |  |  |  | 4% HOMO → LUMO+4 | π→π* |
|  |  |  |  | 35HOMO → LUMO+6 | π→π* |
|  |  |  |  | 2% HOMO → LUMO+9 | π→π* |
|  |  |  |  | 4% HOMO → LUMO+11 | π→π* |
|  | 11 | 3.9184(316.42) | 0.0713 | 4% HOMO-1 → LUMO+3 | π→π* |
|  |  |  |  | 9% HOMO-1 → LUMO+7 | π→π* |
|  |  |  |  | 12% HOMO → LUMO+6 | π→π* |
|  |  |  |  | 68% HOMO → LUMO+7 | π→π* |
|  |  |  |  | 7% HOMO → LUMO+11 | π→π* |
|  | 12 | 3.9382(314.82) | 0.0722 | 2% HOMO-2 → LUMO+2 | π→π* |
|  |  |  |  | 25% HOMO-1 → LUMO+2 | π→π* |
|  |  |  |  | 4% HOMO-1 → LUMO+3 | π→π* |
|  |  |  |  | 6% HOMO-1 → LUMO+7 | π→π* |
|  |  |  |  | 5% HOMO → LUMO+2 | π→π* |
|  |  |  |  | 7% HOMO → LUMO+6 | π→π* |
|  |  |  |  | 13% HOMO → LUMO+7 | π→π* |
|  |  |  |  | 12% HOMO → LUMO+8 | π→π* |
|  |  |  |  | 3% HOMO → LUMO+9 | π→π* |
|  |  |  |  | 21% HOMO → LUMO+11 | π→π* |

**Table S18** Calculated TDDFT excitation energies (*E*), oscillator strengths (*f*), MO compositions and characters for **CF3-H**.

| **HTM** | **n** | **E(eV, nm)** | **f** | **Composition** | **Character** |
| --- | --- | --- | --- | --- | --- |
| **CF_3_-H** | 1 | 2.3512(527.32) | 1.6358 | HOMO → LUMO | CT |
|  | 2 | 2.7435(451.91) | 0.1229 | 2% HOMO-2 → LUMO+1 | π→π* |
|  |  |  |  | 5% HOMO-1 → LUMO | π→π* |
|  |  |  |  | 93% HOMO → LUMO+1 | π→π* |
|  | 3 | 2.7655(448.32) | 0.0712 | 95% HOMO-1 → LUMO | π→π* |
|  |  |  |  | 5% HOMO → LUMO+1 | π→π* |
|  | 4 | 2.9046(426.86) | 0.0013 | 3% HOMO-2 → LUMO+2 | π→π* |
|  |  |  |  | 97% HOMO → LUMO+2 | π→π* |
|  | 5 | 3.0377(408.16) | 0.0493 | 3% HOMO-2 → LUMO+3 | π→π* |
|  |  |  |  | 97% HOMO → LUMO+3 | π→π* |
|  | 6 | 3.1101(398.65) | 0.0115 | 3% HOMO-2 → LUMO+4 | π→π* |
|  |  |  |  | 97% HOMO → LUMO+4 | π→π* |
|  | 7 | 3.2005(387.39) | 0.0065 | HOMO-1 → LUMO+1 | π→π* |
|  | 8 | 3.2979(375.95) | 0.1153 | HOMO-2 → LUMO | π→π* |
|  | 9 | 3.3607(368.92) | 0.0025 | HOMO-1 → LUMO+2 | π→π* |
|  | 10 | 3.4097(363.62) | 0.1710 | HOMO → LUMO+5 | π→π* |
|  | 11 | 3.4986(354.39) | 0.0009 | HOMO-1 → LUMO+3 | π→π* |
|  | 12 | 3.5166(352.56) | 0.0151 | 15% HOMO-1 → LUMO+6 | π→π* |
|  |  |  |  | 5% HOMO-1 → LUMO+7 | π→π* |
|  |  |  |  | 77% HOMO → LUMO+6 | π→π* |
|  |  |  |  | 4% HOMO → LUMO+7 | π→π* |

**Table S19** Calculated TDDFT excitation energies (*E*), oscillator strengths (*f*), MO compositions and characters for **CF3-mF**.

| **HTM** | **n** | **E(eV, nm)** | **f** | **Composition** | **Character** |
| --- | --- | --- | --- | --- | --- |
| **CF_3_-mF** | 1 | 2.4259(511.08) | 1.6270 | HOMO → LUMO | CT |
|  | 2 | 2.7874(444.80) | 0.1374 | 2% HOMO-2 → LUMO+1 | π→π* |
|  |  |  |  | 98% HOMO → LUMO+1 | π→π* |
|  | 3 | 2.8995(427.61) | 0.0835 | HOMO-1 → LUMO | π→π* |
|  | 4 | 2.9517(420.04) | 0.0004 | 2% HOMO-2 → LUMO+2 | π→π* |
|  |  |  |  | 98% HOMO → LUMO+2 | π→π* |
|  | 5 | 3.0825(402.22) | 0.0548 | 2% HOMO-2 → LUMO+3 | π→π* |
|  |  |  |  | 98% HOMO → LUMO+3 | π→π* |
|  | 6 | 3.1489(393.74) | 0.0125 | 2% HOMO-2 → LUMO+4 | π→π* |
|  |  |  |  | 98% HOMO → LUMO+4 | π→π* |
|  | 7 | 3.3201(373.44) | 0.0061 | HOMO-1 → LUMO+1 | π→π* |
|  | 8 | 3.3896(365.78) | 0.0569 | HOMO-2 → LUMO | π→π* |
|  | 9 | 3.4769(356.60) | 0.1731 | 14% HOMO-1 → LUMO+2 | π→π* |
|  |  |  |  | 86% HOMO → LUMO+5 | π→π* |
|  | 10 | 3.4888(355.38) | 0.0228 | 88% HOMO-1 → LUMO+2 | π→π* |
|  |  |  |  | 12% HOMO → LUMO+5 | π→π* |
|  | 11 | 3.6217(342.34) | 0.0005 | HOMO-1 → LUMO+3 | π→π* |
|  | 12 | 3.6557(339.15) | 0.0131 | 7% HOMO-1 → LUMO+6 | π→π* |
|  |  |  |  | 7% HOMO-1 → LUMO+7 | π→π* |
|  |  |  |  | 74% HOMO → LUMO+6 | π→π* |
|  |  |  |  | 12% HOMO → LUMO+7 | π→π* |

**Table S20** Calculated TDDFT excitation energies (*E*), oscillator strengths (*f*), MO compositions and characters for **CF3-oF**.

| **HTM** | **n** | **E(eV, nm)** | **f** | **Composition** | **Character** |
| --- | --- | --- | --- | --- | --- |
| **CF_3_-oF** | 1 | 2.3895(518.86) | 1.6338 | HOMO → LUMO | CT |
|  | 2 | 2.8085(441.46) | 0.1379 | 14% HOMO-1 → LUMO | π→π* |
|  |  |  |  | 86% HOMO → LUMO+1 | π→π* |
|  | 3 | 2.8199(439.67) | 0.0676 | 86% HOMO-1 → LUMO | π→π* |
|  |  |  |  | 14% HOMO → LUMO+1 | π→π* |
|  | 4 | 2.9837(415.53) | 0.0016 | 3% HOMO-2 → LUMO+2 | π→π* |
|  |  |  |  | 97% HOMO → LUMO+2 | π→π* |
|  | 5 | 3.1084(398.87) | 0.0429 | 3% HOMO-2 → LUMO+3 | π→π* |
|  |  |  |  | 97% HOMO → LUMO+3 | π→π* |
|  | 6 | 3.1717(390.91) | 0.0127 | 3% HOMO-2 → LUMO+4 | π→π* |
|  |  |  |  | 97% HOMO → LUMO+4 | π→π* |
|  | 7 | 3.2924(376.58) | 0.0027 | 9% HOMO-2 → LUMO | π→π* |
|  |  |  |  | 91% HOMO-1 → LUMO+1 | π→π* |
|  | 8 | 3.3119(374.36) | 0.1044 | 91% HOMO-2 → LUMO | π→π* |
|  |  |  |  | 9% HOMO-1 → LUMO+1 | π→π* |
|  | 9 | 3.4206(362.46) | 0.2178 | HOMO → LUMO+5 | π→π* |
|  | 10 | 3.4708(357.22) | 0.0017 | HOMO-1 → LUMO+2 | π→π* |
|  | 11 | 3.5670(347.59) | 0.0030 | 3% HOMO-1 → LUMO+6 | π→π* |
|  |  |  |  | 10% HOMO-1 → LUMO+7 | π→π* |
|  |  |  |  | 20% HOMO → LUMO+6 | π→π* |
|  |  |  |  | 66% HOMO → LUMO+7 | π→π* |
|  | 12 | 3.5844(345.90) | 0.0348 | 4% HOMO-1 → LUMO+3 | π→π* |
|  |  |  |  | 19% HOMO-1 → LUMO+6 | π→π* |
|  |  |  |  | 57% HOMO → LUMO+6 | π→π* |
|  |  |  |  | 20% HOMO → LUMO+7 | π→π* |

**Table S21** Summary of the fitting results and corresponding dynamic parameters derived from TRPL decay traces.

| HTM | A_1_ (%) | τ_1_ (ns) | A_2_ (%) | τ_2_ (ns) | τ_avg._ (ns) |
| --- | --- | --- | --- | --- | --- |
| PVSK | 26.57 | 4.60 | 73.42 | 35.30 | 24.21 |
| **Me-H** | 45.96 | 4.05 | 54.03 | 21.95 | 11.65 |
| **Ph-H** | 46.16 | 2.38 | 53.84 | 15.92 | 8.76 |
| **CF3-H** | 45.29 | 2.14 | 54.70 | 15.19 | 8.15 |
| **CF3-mF** | 60.05 | 1.34 | 39.95 | 14.08 | 5.49 |
| **CF3-oF** | 50.46 | 1.14 | 49.53 | 11.11 | 5.41 |
| Spiro-OMeTAD | 47.81 | 1.43 | 52.18 | 11.97 | 6.34 |

**Table S22.** Photovoltaic parameters extracted from *J*-*V* measurements of PSCs based on the various HTMs without dopnats.

| HTM | Scan direction | *V*_oc_ [V] | *J*_sc_ [mA cm^-2^] | FF (%) | PCE_max_ [%] |
| --- | --- | --- | --- | --- | --- |
| **Me-H** | Reverse | 1.03 | 23.28 | 44.36 | 10.64 |
|  | Forward | 1.00 | 23.26 | 44.36 | 10.32 |
| **Ph-H** | Reverse | 0.93 | 23.57 | 51.50 | 11.29 |
|  | Forward | 0.95 | 23.64 | 48.16 | 10.82 |
| **CF-H** | Reverse | 1.01 | 23.99 | 50.50 | 12.24 |
|  | Forward | 0.99 | 23.89 | 51.72 | 12.23 |
| **CF-mF** | Reverse | 1.05 | 23.53 | 51.65 | 12.76 |
|  | Forward | 1.03 | 23.51 | 52.42 | 12.69 |
| **CF-oF** | Reverse | 1.03 | 23.51 | 56.62 | 13.71 |
|  | Forward | 1.00 | 23.48 | 57.37 | 13.47 |
| **Spiro-OMeTAD** | Reverse | 1.03 | 23.80 | 50.48 | 12.37 |
|  | Forward | 1.04 | 23.89 | 49.73 | 12.36 |

References

[1] H. R. Tseng, H. Phan, C. Luo, M. Wang, L. A. Perez, S. N. Patel, L. Ying, E. J. Kramer, T. Q. Nguyen, G. C. Bazan, A. J. Heeger, Adv. Mater. 2014, 26, 2993−2998.

[2] Gaussian 16, Revision A.03, M. J. Frisch, G. W. Trucks, H. B. Schlegel, G. E. Scuseria, M. A. Robb, J. R. Cheeseman, G. Scalmani, V. Barone, G. A. Petersson, H. Nakatsuji, X. Li, M. Caricato, A. V. Marenich, J. Bloino, B. G. Janesko, R. Gomperts, B. Mennucci, H. P. Hratchian, J. V. Ortiz, A. F. Izmaylov, J. L. Sonnenberg, D. Williams-Young, F. Ding, F. Lipparini, F. Egidi, J. Goings, B. Peng, A. Petrone, T. Henderson, D. Ranasinghe, V. G. Zakrzewski, J. Gao, N. Rega, G. Zheng, W. Liang, M. Hada, M. Ehara, K. Toyota, R. Fukuda, J. Hasegawa, M. Ishida, T. Nakajima, Y. Honda, O. Kitao, H. Nakai, T. Vreven, K. Throssell, J. A. Montgomery, Jr., J. E. Peralta, F. Ogliaro, M. J. Bearpark, J. J. Heyd, E. N. Brothers, K. N. Kudin, V. N. Staroverov, T. A. Keith, R. Kobayashi, J. Normand, K. Raghavachari, A. P. Rendell, J. C. Burant, S. S. Iyengar, J. Tomasi, M. Cossi, J. M. Millam, M. Klene, C. Adamo, R. Cammi, J. W. Ochterski, R. L. Martin, K. Morokuma, O. Farkas, J. B. Foresman, and D. J. Fox, Gaussian, Inc., Wallingford CT, 2016.

[3] S. Grimme, C. Bannwarth, P. Shushkov, A Robust and Accurate Tight-Binding Quantum Chemical Method for Structures, Vibrational Frequencies, and Noncovalent Interactions of Large Molecular Systems Parametrized for All spd-Block Elements (Z = 1-86), J. Chem. Theory Comput., 2017, 13 (5), pp 1989–2009

[4] AMS DFTB 2022.1, SCM, Theoretical Chemistry, Vrije Universiteit, Amsterdam, The Netherlands, http://www.scm.com. Optionally, you may add the following list of authors and contributors: R. Rüger, A. Yakovlev, P. Philipsen, S. Borini, P. Melix, A.F. Oliveira, M. Franchini, T. van Vuren, T. Soini, M. de Reus, M. Ghorbani Asl, T. Q. Teodoro, D. McCormack, S. Patchkovskii, T. Heine.

[5] AMS 2022.1, SCM, Theoretical Chemistry, Vrije Universiteit, Amsterdam, The Netherlands, <http://www.scm.com>.

[6] B.o. Xu, J. Zhang, Y. Hua, P. Liu, L. Wang, C. Ruan, Y. Li, G. Boschloo, E.M. J. Johansson, L. Kloo, A. Hagfeldt, A.-Y. Jen, L. Sun, Chem. 2017, 2, 676–687.
